# Comparative effectiveness of various combined interventions for type 2 diabetes and obesity: a systematic review and network meta-analysis

**DOI:** 10.3389/fendo.2025.1462104

**Published:** 2025-08-06

**Authors:** Li Cui, Donglei Lu, Sijie Tan, Liquan Cao

**Affiliations:** Tianjin Institute of Physical Education, Tianjin Key Laboratory of Sports and Health Integration and Health Promotion, Tianjin, China

**Keywords:** type 2 diabetes mellitus, obesity, insulin resistance, aerobic training, resistance training, combined aerobic and resistance training

## Abstract

**Background:**

Type 2 diabetes mellitus (T2DM) is a leading cause of severe complications, projected to affect 693 million adults globally by 2045. Addressing obesity, a key factor in T2DM, through exercise can improve metabolic health and reduce inflammation. This study conducts a Bayesian network meta-analysis to evaluate the long-term effects of various combined interventions on inflammatory markers and metabolic health in overweight or obese individuals with T2DM.

**Methods:**

We included randomized controlled trials (RCTs) from January 2000 to April 2023 that examined the effects of aerobic training (AT), resistance training (RT), combined aerobic and resistance training (ART), physical-mental training (PMT), whole-body vibration training (WBT), and acupuncture (ACT) on BMI, lipid profiles, fasting blood glucose (FBG), HbA1c, HOMA-IR, IL-6, and TNF-α. A comprehensive literature search was performed in PubMed, Web of Science, CNKI, MEDLINE, EMBASE, and the Cochrane Central Register of Controlled Trials. Data extraction and quality assessment were independently conducted by two researchers, and Bayesian network meta-analysis was performed using R software.

**Results:**

A total of 128 RCTs were included. ART showed the most significant improvements in BMI, IL-6, and TNF-α levels. PMT was the most effective in improving lipid profiles (TG, TC, HDL-C, LDL-C) and insulin sensitivity markers (HbA1c, HOMA-IR). The SUCRA rankings indicated ART and PMT as the most beneficial interventions. Meta-regression analysis highlighted that VO_2max_ improvements were closely associated with reductions in BMI and HbA1c.

**Conclusion:**

ART and PMT demonstrated comprehensive benefits across multiple metabolic and inflammatory outcomes. ART effectively reduced BMI, improved glycemic control, and decreased inflammatory markers through mechanisms involving AMPK and mTOR pathways. PMT improved lipid metabolism and insulin sensitivity by reducing stress hormone levels and modulating endocrine and nervous system functions. A precise exercise prescription combining ART, PMT, AT, RT, and ACT is recommended to optimize metabolic health in T2DM patients. Future research should focus on individualized intervention strategies to enhance clinical outcomes.

**Systematic review registration:**

PROSPERO, identifier CRD42024539376.

## Highlights

- Exercise can be an effective way to reduce metabolic and inflammatory markers in overweight or obese individuals with T2DM.
- Different types of exercise, including ART and PMT, have been found to be effective in improving multiple health indicators.
- ART and PMT were identified as the most beneficial interventions, with ART significantly reducing BMI, IL-6, and TNF-α levels, and PMT improving lipid profiles and insulin sensitivity.

## Introduction

Type 2 diabetes (T2DM) is a major contributor to kidney failure, peripheral neuropathy, retinopathy, and cardiovascular disease (CVD) (1). By 2045, it is projected to affect 693 million adults globally (2). T2DM arises from a combination of genetic and environmental factors, with obesity, driven by high-energy diets and sedentary lifestyles, being a significant cause (3, 4). Obesity-induced insulin resistance (IR) leads to increased lipolysis and elevated free fatty acids (FFA), which further worsen IR. Addressing obesity is thus essential for improving T2DM outcomes (5).

Exercise interventions are effective in improving blood sugar levels, weight, blood lipids, and blood pressure in overweight or obese T2DM patients. They also help prevent CVD, reduce mortality, and enhance quality of life (6). Aerobic training (AT) enhances blood sugar control and body composition by improving aerobic capacity and metabolism of fats and glucose, thus boosting insulin sensitivity (7). Resistance training (RT) promotes metabolic health not only by improving body composition—via increased muscle mass, elevated basal metabolic rate (BMR), and enhanced insulin sensitivity—but also by modulating pancreatic β-cell function. Recent studies implicate RT in the regulation of muscle–pancreas crosstalk, suggesting a mechanistic link between skeletal muscle activity and β-cell insulin secretory capacity, ultimately contributing to systemic glucose homeostasis (8). Combined aerobic and resistance training (ART) offers comprehensive benefits, improving both metabolic health and body composition (9). Recently, physical-mental training (PMT) and whole-body vibration training (WBT) have gained attention. Mind-body exercises like yoga and tai chi reduce stress, enhance mental health, and improve insulin sensitivity and blood sugar control by combining physical movement with breath control (10). WBT stimulates muscle contraction through mechanical vibrations, increasing muscle strength and metabolic rate, benefiting weight control and metabolic health (11). Acupuncture therapy (ACT), a traditional Chinese medicine intervention, has shown promise in treating obese T2DM patients. It regulates various metabolic pathways, reduces appetite, slows gastric emptying, improves insulin sensitivity, increases basal metabolic rate (BMR), and accelerates fat consumption through multiple mechanisms¹². Although ACT is not a physical activity-based intervention, it was included in the present study due to its emerging evidence in metabolic regulation and its potential relevance to cardiovascular and autonomic function. Including ACT thus offers a complementary perspective to conventional exercise-based strategies for managing subclinical myocardial dysfunction in individuals with type 2 diabetes (12).

Inflammation, particularly involving interleukin-6 (IL-6) and tumor necrosis factor-alpha (TNF-α), plays a crucial role in the pathogenesis of obesity and T2DM (13). Elevated IL-6 and TNF-α levels are closely linked to IR. Exercise interventions reduce IL-6 and TNF-α levels, improving insulin sensitivity by decreasing macrophage infiltration in adipose tissue and modulating pro- and anti-inflammatory factors (14). PMT and WBT also show potential in reducing these inflammatory markers by affecting the neuroendocrine system and mitigating chronic inflammation.

Despite the benefits of these interventions, a systematic comparison is lacking. This study employs a systematic review and meta-analysis to evaluate the impact of combined interventions on T2DM and obesity, aiming to provide a basis for developing more effective intervention strategies.

## Methods

This study adhered to the Preferred Reporting Items for Systematic Reviews and Meta-Analyses (PRISMA) extension for network meta-analysis (PRISMA NMA) guidelines (15). The study protocol was registered with PROSPERO (registration number CRD42024539376). Ethical approval and informed consent were deemed unnecessary due to the nature of this research.

### Study design and participants

We included randomized controlled trials (RCTs) published from January 2000 to April 2023, focusing on overweight or obese individuals diagnosed with type 2 diabetes mellitus (T2DM). The criteria for overweight and obesity were defined according to ethnicity-specific guidelines: BMI ≥25 kg/m² (overweight) or ≥30 kg/m² (obesity) for Europeans (16), and BMI ≥23 kg/m² (overweight) or ≥27 kg/m² (obesity) for Asians (17). These lower thresholds for Asian populations reflect their higher percentage of body fat and increased cardiometabolic risk at lower BMI levels, as recommended by the World Health Organization Expert Consultation (2004). In studies where BMI data were not reported, body fat percentage (BF%) was used as an alternative index of adiposity. Participants were classified as overweight or obese if BF% was ≥30% for women and ≥25% for men, which are widely accepted cut-off values associated with elevated risk of metabolic syndrome and cardiovascular disease. In studies where BMI was not provided, body fat percentage (BF%) was used (BF% ≥30% for women and ≥25% for men). T2DM diagnosis had to meet the criteria of the International Diabetes Federation (IDF) (18) or the American Diabetes Association (ADA) (19).

### Interventions

Interventions included aerobic training (AT), resistance training (RT), combined aerobic and resistance training (ART), physical-mental training (PMT, such as Baduanjin, Tai Chi, yoga, Pilates, etc.), whole-body vibration training (WBT), and acupuncture (ACT), each lasting for a minimum of four weeks. Control groups received either no intervention or standard diabetes medication.

Outcome Measures

Primary outcomes were changes in BMI, lipid profiles (TG, TC, HDL-C, LDL-C), fasting blood glucose (FBG), glycosylated hemoglobin (HbA1c), homeostasis model assessment of insulin resistance (HOMA-IR), IL-6, and TNF-α levels pre- and post-intervention. Secondary outcomes included withdrawal risk differences at eight weeks.

### Literature search strategy

We systematically searched PubMed, Web of Science, CNKI, MEDLINE, EMBASE, and the Cochrane Central Register of Controlled Trials from January 2000 to April 2024. A combination of subject terms and free terms was used for the search, and only core journals were included in the Chinese literature. References of the included studies were also reviewed to identify additional relevant studies.

### Literature screening and data extraction

Two researchers independently screened the literature, extracted data, and cross-checked the results. Disagreements were resolved through discussion with a third party. Authors were contacted to supplement missing data when necessary. Data extraction included: first author, publication year, intervention subjects (sample size, gender, age, BMI), intervention details (type, duration, frequency), and outcome measures. Outcome data were converted into mean and standard deviation (SD) of the pre- and post-intervention differences using the following formula (20, 21):


$$M=M_{2}-M_{1}$$

$$SD=\sqrt{SD_{1}^{2}+SD_{2}^{2}-2\times R\times SD_{1}\times SD_{2}}$$

where M is the mean difference, SD is the standard deviation of the difference, and R is the correlation coefficient (assumed to be 0.5). In cases where original studies did not report the SD of the change between pre- and post-intervention values, we estimated this value using a widely accepted method based on the variance structure of paired data. This approach assumes that each participant provides two correlated measurements (before and after the intervention), and the variability of the change depends not only on the individual SDs at each time point but also on the degree of correlation between them. To account for this correlation, we followed the recommendation of the Cochrane Handbook for Systematic Reviews of Interventions, which advises using an assumed correlation coefficient (R) of 0.5 when the actual value is not reported. This moderate assumption balances the need to reflect within-subject consistency without overestimating or underestimating variability. The use of this method allows for the inclusion of studies lacking direct change score SDs and enables the standardized calculation of intervention effects across trials. It is widely applied in meta-analytical research to maximize data availability and maintain methodological consistency (21).

### Risk of bias assessment

The quality of included studies was assessed using the Cochrane Risk of Bias (RoB 2.0) tool, evaluating randomization, allocation concealment, blinding, completeness of outcome data, selective reporting, and other biases. Each domain was rated as “high risk”, “low risk”, or “unclear” (22).

### Statistical analysis

Bayesian network meta-analysis was conducted using the Gemtc package in R 4.3.2, with sampling simulated via JAGS 4.3.1 generalized linear models. Missing SDs were calculated from t statistics and p-values of mean differences according to Cochrane guidelines handbooks (23). A random effects model was used to estimate effect sizes and 95% confidence intervals (CI). Surface under the cumulative ranking (SUCRA) values were calculated to rank interventions, and results were presented as ranking tables and forest plots using ggplot2. Publication bias was assessed using comparison-corrected funnel plots, with p< 0.05 considered statistically significant.

## Results

### Literature search and screening

**Figure 1:**
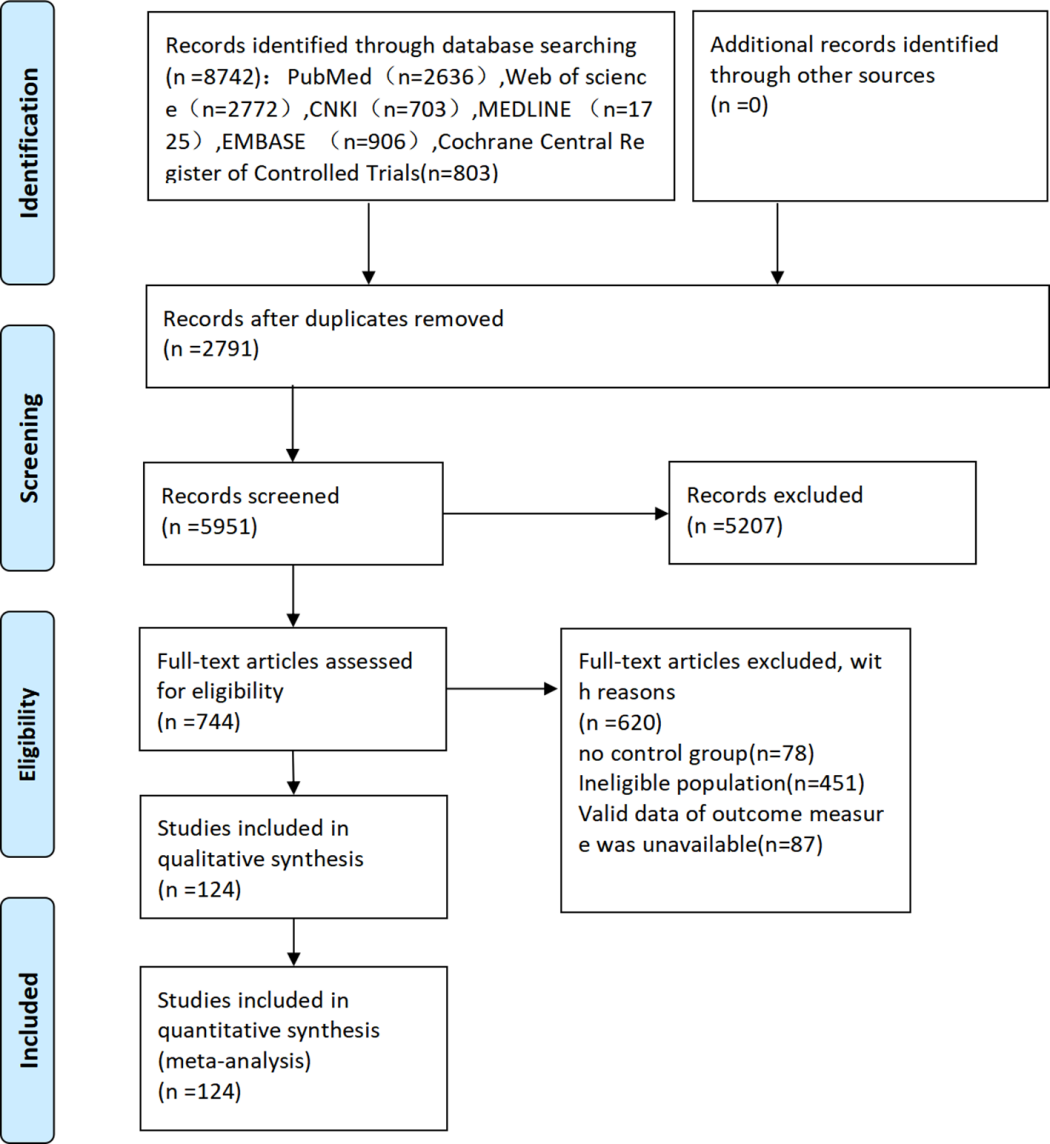
Flow chart of literature screening.

A total of 8742 articles were retrieved, including 8039 in English and 703 in Chinese. Ultimately, 124 articles (24–48) were included. The literature screening process and results are illustrated in **Figure 1**.

### Basic characteristics of literature and quality assessment results

**Figure 2:**
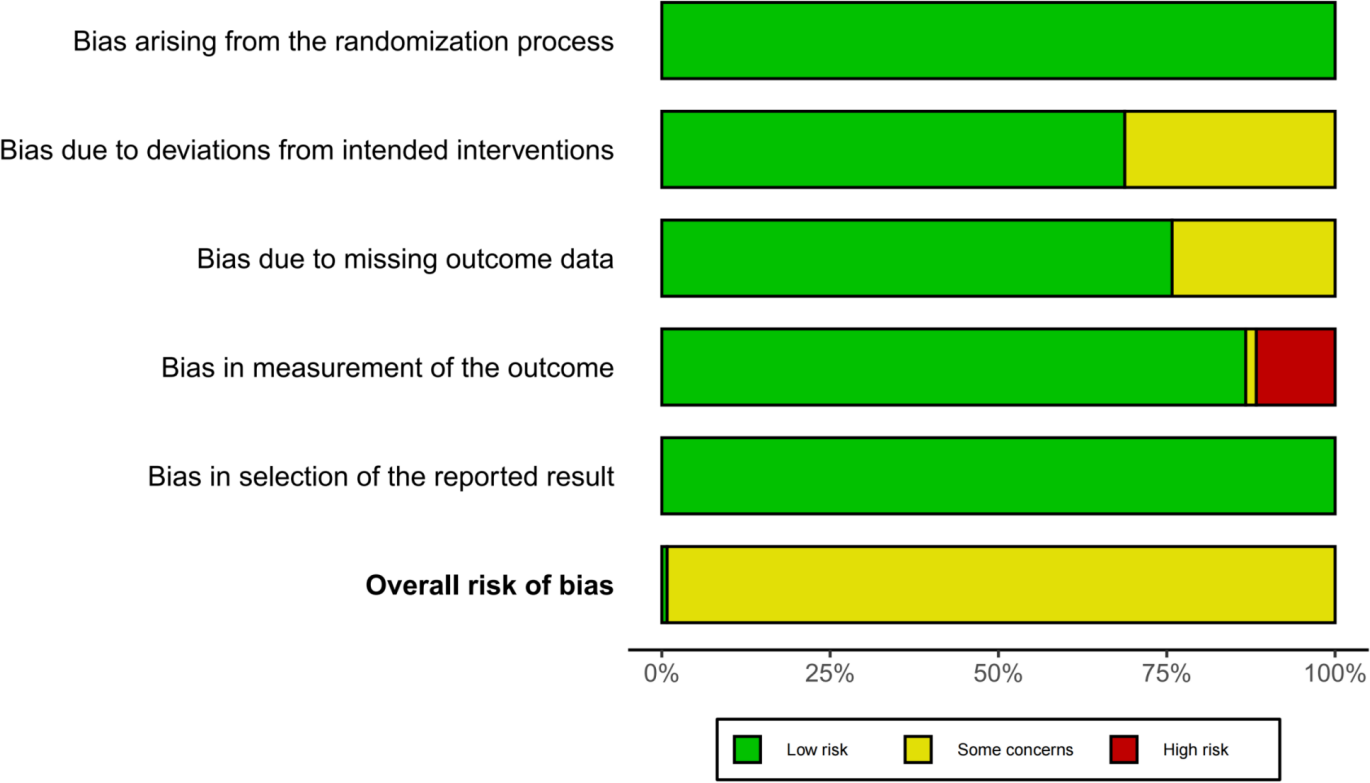
Summary of literature quality assessment.

The 124 included articles are presented in **Supplementary Table 1**. All patients were treated with conventional medications for diabetes and metabolic syndrome, and the baseline characteristics between the two groups were comparable. The quality and risk assessments are summarized in **Figure 2**.

### Network relationship and inconsistency analysis

#### Network relationship diagram

**Figure 3:**
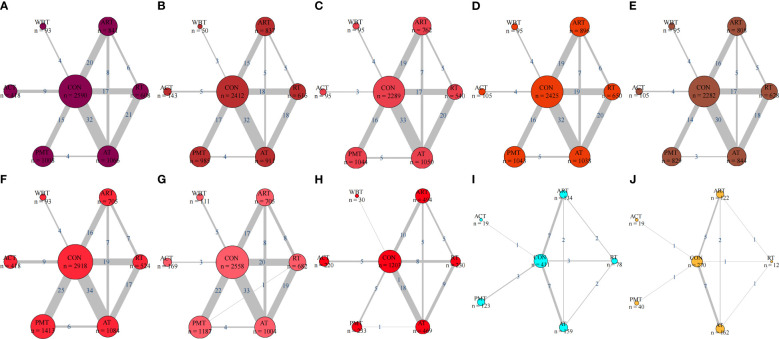
**(A)** Network relationship diagram of BMI outcome indicators. **(B)** Network relationship diagram of TG outcome indicators. **(C)** Network relationship diagram of TC outcome indicators. **(D)** Network relationship diagram of HDL-C outcome indicators. **(E)** Network relationship diagram of LDL-C outcome indicators. **(F)** Network relationship diagram of FBG outcome indicators. **(G)** Network relationship diagram of HbA1c% outcome indicators. **(H)** Network relationship diagram of HOMA-IR outcome indicators. **(I)** Network relationship diagram of IL-6 outcome indicators. **(J)** Network relationship diagram of TNF-α outcome indicators eta-analysis forest plot.

The network evidence diagram with BMI, TG, TC, HDL-C, LDL-C, FBG, HbA1c, HOMA-IR, IL-6, and TNF-α as outcome indicators is presented in **Figures 3A–J**.

This study constructed ten separate network meta-analysis models targeting key metabolic and inflammatory outcomes in patients with type 2 diabetes mellitus, including body mass index (BMI), triglycerides (TG), total cholesterol (TC), high-density lipoprotein cholesterol (HDL-C), low-density lipoprotein cholesterol (LDL-C), fasting blood glucose (FBG), glycated hemoglobin (HbA1c%), homeostasis model assessment of insulin resistance (HOMA-IR), interleukin-6 (IL-6), and tumor necrosis factor-alpha (TNF-α). Each network was centered on the control group (CON), which was directly compared with six types of non-pharmacological interventions: aerobic training (AT), resistance training (RT), combined aerobic and resistance training (ART), psychomotor training (PMT), acupuncture (ACT), and whole-body therapy (WBT). The network structures for metabolic outcomes such as BMI, blood lipids, FBG, and HbA1c% were generally dense and well-connected, with frequent direct comparisons between CON and major interventions like AT, PMT, and RT—often supported by more than 15 trials. In addition, several active interventions were directly compared (e.g., AT vs RT, ART vs AT), forming multiple triangular loops that support consistency checks and robust indirect comparisons. The network for HOMA-IR was moderately connected, while those for IL-6 and TNF-α were sparse, with very few direct comparisons among active interventions and limited representation for treatments like ACT and RT. Despite these variations in network density, all interventions were involved in at least one direct or indirect comparison across most outcomes, ensuring broad intervention coverage and acceptable structural comparability. Collectively, these networks provide a strong methodological basis for estimating relative treatment effects, performing SUCRA-based rankings, and conducting subgroup or sensitivity analyses across diverse physiological domains.

#### Heterogeneity test

This network meta-analysis comprised 124 studies. Heterogeneity tests revealed that studies evaluating the impact of exercise on body composition, glycolipid metabolism, and inflammatory markers among populations with obesity and type 2 diabetes exhibited substantial heterogeneity (I^2^ > 50%, P< 0.1). Utilizing the data from this meta-analysis, individual studies were sequentially excluded to pinpoint sources of heterogeneity (refer to **Table 1**). Although some groups displayed persistent heterogeneity, the others were largely homogeneous. To enhance the precision of the findings, a random effects model was employed in the analyses.

**Table 1:** Heterogeneity analysis.

			Pre-exclusion		Post-exclusion	
Outcomes	Comparison	Traditional/Network	I2/%	P	I2/%	P
BMI	RT-CON	Pooled (pair-wise)	84.90%	0.001	43.30%	0.020
		Pooled (network)	83.80%	0.001	38.50%	0.030
	ART-CON	Pooled (pair-wise)	97.50%	0.906	69.50%	0.060
		Pooled (network)	98.50%	0.906	58.50%	0.084
	PMT-CON	Pooled (pair-wise)	91.10%	0.603	91.10%	0.603
		Pooled (network)	88.10%	0.073	88.10%	0.073
	ACT-CON	Pooled (pair-wise)	60.50%	0.05	21.20%	0.000
		Pooled (network)	60.60%	0.05	17.60%	0.000
	AT-CON	Pooled (pair-wise)	52.20%	0.05	32.20%	0.027
		Pooled (network)	39.00%	0.05	39.00%	0.05
	WBT-CON	Pooled (pair-wise)	0.00%	0.035	0.00%	0.035
		Pooled (network)	0.00%	0.075	0.00%	0.075
TG	RT-CON	Pooled (pair-wise)	68.60%	0.169	0.00%	0.239
		Pooled (network)	99.40%	0.063	47.00%	0.017
	ART-CON	Pooled (pair-wise)	99.40%	0.075	39.40%	0.013
		Pooled (network)	99.30%	0.054	33.80%	0.020
	PMT-CON	Pooled (pair-wise)	93.40%	0.001	83.70%	0.121
		Pooled (network)	93.00%	0.005	92.40%	0.031
	ACT-CON	Pooled (pair-wise)	25.50%	0.228	25.50%	0.228
		Pooled (network)	31.40%	0.154	31.40%	0.154
	AT-CON	Pooled (pair-wise)	99.40%	0.187	92.60%	0.179
		Pooled (network)	99.70%	0.068	99.70%	0.018
	WBT-CON	Pooled (pair-wise)	73.80%	0.075	37.20%	0.041
		Pooled (network)	73.60%	0.056	39.40%	0.056
TC	RT-CON	Pooled (pair-wise)	91.70%	0.061	47.20%	0.061
		Pooled (network)	98.70%	0.000	49.40%	0.000
	ART-CON	Pooled (pair-wise)	96.40%	0.049	96.40%	0.000
		Pooled (network)	96.10%	0.002	94.20%	0.002
	PMT-CON	Pooled (pair-wise)	84.80%	0.211	27.40%	0.120
		Pooled (network)	87.20%	0.198	37.70%	0.079
	ACT-CON	Pooled (pair-wise)	66.70%	0.048	28.90%	0.120
		Pooled (network)	65.30%	0.051	34.60%	0.014
	AT-CON	Pooled (pair-wise)	0.00%	0.121	0.00%	0.121
		Pooled (network)	0.00%	0.154	0.00%	0.154
	WBT-CON	Pooled (pair-wise)	89.30%	0.101	34.70%	0.000
		Pooled (network)	96.10%	0.254	51.30%	0.053
HDL-C	RT-CON	Pooled (pair-wise)	91.50%	0.155	41.70%	0.010
		Pooled (network)	98.50%	0.103	46.40%	0.032
	ART-CON	Pooled (pair-wise)	99.30%	0.121	41.90%	0.073
		Pooled (network)	98.30%	0.231	40.90%	0.183
	PMT-CON	Pooled (pair-wise)	91.40%	0.055	34.00%	0.007
		Pooled (network)	92.60%	0.063	35.20%	0.015
	ACT-CON	Pooled (pair-wise)	45.90%	0.061	45.90%	0.061
		Pooled (network)	50.10%	0.073	43.20%	0.030
	AT-CON	Pooled (pair-wise)	87.20%	0.059	38.90%	0.024
		Pooled (network)	86.90%	0.062	34.10%	0.040
	WBT-CON	Pooled (pair-wise)	20.10%	0.081	20.10%	0.081
		Pooled (network)	17.60%	0.095	17.60%	0.095
LDL-C	RT-CON	Pooled (pair-wise)	96.90%	0.085	39.10%	0.125
		Pooled (network)	98.60%	0.062	40.80%	0.014
	ART-CON	Pooled (pair-wise)	97.80%	0.077	40.00%	0.121
		Pooled (network)	97.70%	0.063	39.90%	0.1200
	PMT-CON	Pooled (pair-wise)	93.30%	0.003	35.50%	0.17
		Pooled (network)	93.20%	0.011	35.40%	0.03
	ACT-CON	Pooled (pair-wise)	48.60%	0.095	48.60%	0.025
		Pooled (network)	48.20%	0.063	48.20%	0.007
	AT-CON	Pooled (pair-wise)	99.20%	0.002	86.40%	0.069
		Pooled (network)	98.90%	0.032	81.70%	0.721
	WBT-CON	Pooled (pair-wise)	0.00%	0.059	0.00%	0.011
		Pooled (network)	0.00%	0.063	0.00%	0.072
FBG	RT-CON	Pooled (pair-wise)	100.00%	0.065	43.20%	0.005
		Pooled (network)	100.00%	0.051	44.70%	0.019
	ART-CON	Pooled (pair-wise)	99.10%	0.076	49.10%	0.006
		Pooled (network)	98.90%	0.084	55.00%	0.014
	PMT-CON	Pooled (pair-wise)	99.90%	0.010	41.00%	0.023
		Pooled (network)	99.90%	0.006	39.00%	0.046
	ACT-CON	Pooled (pair-wise)	66.00%	0.056	38.80%	0.014
		Pooled (network)	65.90%	0.052	42.00%	0.018
	AT-CON	Pooled (pair-wise)	96.40%	0.008	44.20%	0.062
		Pooled (network)	95.70%	0.005	32.70%	0.065
	WBT-CON	Pooled (pair-wise)	97.80%	0.069	97.80%	0.399
		Pooled (network)	97.40%	0.076	97.40%	0.395
HbA1c%	RT-CON	Pooled (pair-wise)	99.90%	0.043	99.90%	0.42
		Pooled (network)	100.00%	0.052	100.00%	0.421
	ART-CON	Pooled (pair-wise)	75.20%	0.048	75.20%	0.173
		Pooled (network)	74.40%	0.039	74.40%	0.165
	PMT-CON	Pooled (pair-wise)	98.40%	0.006	98.40%	0.405
		Pooled (network)	98.30%	0.013	98.30%	0.404
	ACT-CON	Pooled (pair-wise)	97.20%	0.133	97.20%	0.393
		Pooled (network)	97.10%	0.162	97.10%	0.392
	AT-CON	Pooled (pair-wise)	86.40%	0.002	83.70%	0.120
		Pooled (network)	93.40%	0.000	79.40%	0.000
	WBT-CON	Pooled (pair-wise)	41.80%	0.074	41.80%	0.074
		Pooled (network)	47.00%	0.069	47.00%	0.069
HOMA-IR	RT-CON	Pooled (pair-wise)	74.00%	0.052	49.20%	0.037
		Pooled (network)	77.90%	0.050	52.70%	0.012
	ART-CON	Pooled (pair-wise)	95.60%	0.005	38.20%	0.021
		Pooled (network)	96.40%	0.008	39.00%	0.027
	PMT-CON	Pooled (pair-wise)	93.50%	0.008	36.10%	0.010
		Pooled (network)	93.70%	0.006	36.30%	0.002
	ACT-CON	Pooled (pair-wise)	98.90%	0.042	41.50%	0.21
		Pooled (network)	99.00%	0.038	41.60%	0.01
	AT-CON	Pooled (pair-wise)	92.30%	0.048	34.90%	0.12
		Pooled (network)	92.00%	0.045	34.60%	0.37
	WBT-CON	Pooled (pair-wise)	57.00%	0.053	57.00%	0.87
		Pooled (network)	51.80%	0.051	51.80%	0.012
IL-6	RT-CON	Pooled (pair-wise)	13.90%	0.098	13.90%	0.024
		Pooled (network)	75.90%	0.152	74.30%	0.004
	ART-CON	Pooled (pair-wise)	86.10%	0.062	39.10%	0.294
		Pooled (network)	84.50%	0.048	37.50%	0.09
	PMT-CON	Pooled (pair-wise)	89.80%	0.003	52.80%	0.003
		Pooled (network)	87.80%	0.006	52.80%	0.007
	ACT-CON	Pooled (pair-wise)	77.80%	0.095	50.80%	0.005
		Pooled (network)	89.80%	0.087	46.80%	0.02
	AT-CON	Pooled (pair-wise)	71.50%	0.031	44.50%	0.013
		Pooled (network)	88.30%	0.047	41.30%	0.078
TNF-α	RT-CON	Pooled (pair-wise)	NA	NA	NA	NA
		Pooled (network)	NA	NA	NA	NA
	ART-CON	Pooled (pair-wise)	69.10%	0.051	34.80%	0.021
		Pooled (network)	67.80%	0.048	39.10%	0.006
	PMT-CON	Pooled (pair-wise)	NA	NA	NA	NA
		Pooled (network)	NA	NA	NA	NA
	ACT-CON	Pooled (pair-wise)	NA	NA	NA	NA
		Pooled (network)	NA	NA	NA	NA
	AT-CON	Pooled (pair-wise)	81.50%	0.056	41.70%	0.023
		Pooled (network)	86.10%	0.048	42.80%	0.003

NA, not available.

#### Inconsistency testing and convergence assessment

Except for the TNF-α outcome indicator, the local inconsistency test results showed P > 0.05, indicating no significant difference between the direct and indirect comparison results. Thus, the consistency model could be used for analysis, while TNF-α was analyzed using an inconsistency model. The PSRFs for all 10 outcome indicators were close to 1, indicating good convergence.

### Network meta-analysis results

#### Body composition

**Figure 4:**
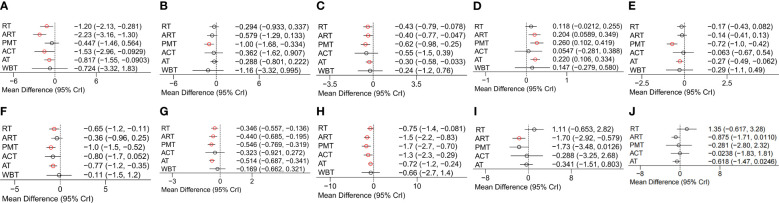
**(A)** BMI network meta-analysis forest plot. **(B)** TG network meta-analysis forest plot. **(C)** TC network meta-analysis forest plot. **(D)** HDL-C network meta-analysis forest plot. **(E)** LDL-C network meta-analysis forest plot. **(F)** FBG network meta-analysis forest plot. **(G)** HbA1c% network meta-analysis forest plot. **(H)** HOMA-IR network meta-analysis forest plot. **(I)** IL-6 network meta-analysis forest plot. **(J)** TNF-α network meta-analysis forest plot.

A total of 87 RCTs (70 two-arm, 12 three-arm, and 5 four-arm) (24–28, 31, 32, 35, 36, 38–40, 42–44, 47–58) reported improvements in BMI outcome indicators for patients with T2DM and overweight or obesity. The network meta-analysis results showed that in terms of improving BMI outcome indicators, RT, ART, ACT, and AT were more effective than the control group as shown in **Figure 4A**. The SUCRA ranking results showed: ART (0.9332) > ACT (0.7064) > RT (0.6159) > AT (0.4404) > WBT (0.4267) > PMT (0.2907) > CON (0.0867). ART was most likely to be the best intervention measure as shown in **Table 2**.

**Table 2:** Overview of network meta-analysis league tables for each outcome indicator.

BMI						
CON	RT	ART	PMT	ACT	AT	WBT
1	-1.2 (-2.13, -0.28)	-2.23 (-3.16, -1.3)	-0.45 (-1.46, 0.56)	-1.53 (-2.96, -0.09)	-0.82 (-1.55, -0.09)	-0.72 (-3.32, 1.83)
	2	-1.02 (-2.19, 0.15)	0.76 (-0.58, 2.09)	-0.33 (-2.02, 1.39)	0.38 (-0.51, 1.29)	0.48 (-2.28, 3.21)
		3	1.78 (0.42, 3.13)	0.7 (-1.01, 2.41)	1.41 (0.35, 2.47)	1.5 (-1.25, 4.23)
			4	-1.08 (-2.83, 0.69)	-0.37 (-1.53, 0.8)	-0.27 (-3.06, 2.5)
				5	0.71 (-0.89, 2.31)	0.8 (-2.16, 3.73)
					6	0.09 (-2.59, 2.76)
						7

TG						
CON	RT	ART	PMT	ACT	AT	WBT
1	-0.29 (-0.93, 0.34)	-0.58 (-1.29, 0.13)	-1 (-1.68, -0.33)	-0.36 (-1.62, 0.91)	-0.29 (-0.8, 0.22)	-1.16 (-3.32, 1)
	2	-0.29 (-1.15, 0.59)	-0.71 (-1.61, 0.2)	-0.07 (-1.47, 1.36)	0.01 (-0.64, 0.65)	-0.86 (-3.11, 1.38)
		3	-0.42 (-1.4, 0.54)	0.22 (-1.23, 1.67)	0.29 (-0.52, 1.09)	-0.58 (-2.86, 1.68)
			4	0.64 (-0.79, 2.08)	0.71 (-0.08, 1.5)	-0.15 (-2.42, 2.1)
				5	0.08 (-1.3, 1.42)	-0.79 (-3.32, 1.7)
					6	-0.87 (-3.09, 1.34)
						7

TC						
CON	RT	ART	PMT	ACT	AT	WBT
1	-0.43 (-0.79, -0.08)	-0.4 (-0.77, -0.05)	-0.62 (-0.98, -0.25)	-0.55 (-1.48, 0.39)	-0.3 (-0.58, -0.03)	-0.24 (-1.23, 0.76)
	2	0.02 (-0.43, 0.48)	-0.19 (-0.68, 0.31)	-0.12 (-1.11, 0.89)	0.13 (-0.22, 0.47)	0.19 (-0.86, 1.26)
		3	-0.21 (-0.71, 0.29)	-0.14 (-1.15, 0.87)	0.1 (-0.31, 0.51)	0.17 (-0.89, 1.23)
			4	0.07 (-0.93, 1.08)	0.31 (-0.11, 0.73)	0.38 (-0.68, 1.44)
				5	0.24 (-0.73, 1.22)	0.3 (-1.05, 1.68)
					6	0.07 (-0.96, 1.1)
						7

HDL-C						
CON	RT	ART	PMT	ACT	AT	WBT
1	0.12 (-0.02, 0.25)	0.2 (0.06, 0.35)	0.26 (0.1, 0.42)	0.05 (-0.28, 0.39)	0.22 (0.11, 0.33)	0.15 (-0.28, 0.58)
	2	0.09 (-0.09, 0.27)	0.14 (-0.06, 0.35)	-0.06 (-0.43, 0.3)	0.1 (-0.04, 0.24)	0.03 (-0.42, 0.48)
		3	0.06 (-0.15, 0.27)	-0.15 (-0.52, 0.21)	0.02 (-0.15, 0.18)	-0.06 (-0.51, 0.4)
			4	-0.21 (-0.58, 0.16)	-0.04 (-0.22, 0.14)	-0.11 (-0.57, 0.35)
				5	0.17 (-0.19, 0.52)	0.09 (-0.45, 0.64)
					6	-0.07 (-0.52, 0.38)
						7

LDL-C						
CON	RT	ART	PMT	ACT	AT	WBT
1	-0.17 (-0.43, 0.08)	-0.14 (-0.41, 0.13)	-0.72 (-1.03, -0.42)	-0.06 (-0.67, 0.54)	-0.27 (-0.49, -0.06)	-0.29 (-1.08, 0.49)
	2	0.03 (-0.3, 0.37)	-0.55 (-0.94, -0.16)	0.11 (-0.55, 0.77)	-0.1 (-0.35, 0.15)	-0.12 (-0.94, 0.72)
		3	-0.58 (-0.99, -0.19)	0.08 (-0.6, 0.74)	-0.13 (-0.45, 0.17)	-0.15 (-0.98, 0.67)
			4	0.65 (-0.02, 1.34)	0.45 (0.1, 0.8)	0.43 (-0.4, 1.27)
				5	-0.21 (-0.86, 0.43)	-0.22 (-1.22, 0.77)
					6	-0.02 (-0.84, 0.8)
						7

FBG						
CON	RT	ART	PMT	ACT	AT	WBT
1	-0.65 (-1.18, -0.11)	-0.36 (-0.96, 0.25)	-1.01 (-1.5, -0.52)	-0.8 (-1.65, 0.05)	-0.77 (-1.19, -0.35)	-0.11 (-1.48, 1.25)
	2	0.29 (-0.43, 1.01)	-0.36 (-1.06, 0.34)	-0.15 (-1.16, 0.85)	-0.12 (-0.68, 0.43)	0.54 (-0.93, 2)
		3	-0.65 (-1.42, 0.12)	-0.44 (-1.49, 0.6)	-0.42 (-1.09, 0.26)	0.25 (-1.26, 1.74)
			4	0.21 (-0.78, 1.19)	0.24 (-0.36, 0.83)	0.9 (-0.56, 2.35)
				5	0.03 (-0.92, 0.98)	0.68 (-0.93, 2.31)
					6	0.66 (-0.77, 2.09)
						7

HbA1c%						
CON	RT	ART	PMT	ACT	AT	WBT
1	-0.35 (-0.56, -0.14)	-0.44 (-0.69, -0.2)	-0.55 (-0.77, -0.32)	-0.32 (-0.92, 0.27)	-0.51 (-0.69, -0.34)	-0.17 (-0.66, 0.32)
	2	-0.09 (-0.37, 0.19)	-0.2 (-0.5, 0.1)	0.02 (-0.61, 0.65)	-0.17 (-0.38, 0.05)	0.18 (-0.36, 0.72)
		3	-0.11 (-0.43, 0.22)	0.12 (-0.53, 0.76)	-0.07 (-0.34, 0.19)	0.27 (-0.28, 0.82)
			4	0.22 (-0.41, 0.86)	0.03 (-0.24, 0.3)	0.38 (-0.17, 0.91)
				5	-0.19 (-0.81, 0.43)	0.16 (-0.62, 0.93)
					6	0.35 (-0.18, 0.87)
						7

HOMA-IR						
CON	RT	ART	PMT	ACT	AT	WBT
1	-0.75 (-1.42, -0.08)	-1.49 (-2.19, -0.83)	-1.69 (-2.69, -0.7)	-1.25 (-2.25, -0.29)	-0.72 (-1.21, -0.24)	-0.66 (-2.73, 1.37)
	2	-0.74 (-1.56, 0.06)	-0.94 (-2.14, 0.25)	-0.5 (-1.71, 0.66)	0.03 (-0.63, 0.68)	0.09 (-2.03, 2.16)
		3	-0.2 (-1.38, 1.02)	0.24 (-0.94, 1.44)	0.78 (0.07, 1.51)	0.83 (-1.28, 2.95)
			4	0.43 (-0.97, 1.82)	0.97 (-0.12, 2.06)	1.03 (-1.24, 3.29)
				5	0.54 (-0.54, 1.64)	0.6 (-1.67, 2.86)
					6	0.05 (-1.94, 2.04)
						7

#### Lipid metabolism

A total of 82 RCTs (68 two-arm, 10 three-arm, and 4 four-arm) (24–26, 29, 32, 34, 36–38, 40–42, 47, 50–53, 57–66) reported improvements in TG outcome indicators for patients with T2DM and overweight or obesity. The network meta-analysis results showed that PMT was more effective than the control group in improving TG outcome indicators as shown in **Figure 4B**. The SUCRA ranking results showed: PMT (0.8277) > WBT (0.7313) > ART (0.5952) > ACT (0.443) > RT (0.3842) > AT (0.3841) > CON (0.1346). PMT was most likely to be the best intervention measure as shown in **Table 2**.

A total of 82 RCTs (66 two-arm, 11 three-arm, and 5 four-arm) (24, 25, 28, 32, 34–37, 40–42, 47, 49–53, 55–61, 63–120) reported improvements in TC outcome indicators for patients with T2DM and overweight or obesity. The network meta-analysis results showed that in terms of improving TC outcome indicators, the effects of RT, ART, PMT, and AT were better than those of the control group as shown in **Figure 4C**. The SUCRA ranking results showed: PMT (0.8019) > ACT (0.6463) > RT (0.5966) > ART (0.5571) > AT (0.4116) > WBT (0.407) > CON (0.0794). PMT was most likely to be the best intervention measure as shown in **Table 3**.

**Table 3:** List of network meta-analysis league tables for each outcome indicator (sequence).

IL-6					
CON	RT	ART	PMT	ACT	AT
1	1.11 (-0.65, 2.82)	-1.7 (-2.92, -0.58)	-1.73 (-3.48, 0.01)	-0.29 (-3.25, 2.68)	-0.34 (-1.51, 0.8)
	2	-2.82 (-4.62, -1.06)	-2.84 (-5.29, -0.34)	-1.41 (-4.81, 2.09)	-1.45 (-3.24, 0.37)
		3	-0.03 (-2.07, 2.12)	1.41 (-1.71, 4.64)	1.36 (-0.04, 2.83)
			4	1.45 (-2, 4.86)	1.39 (-0.73, 3.48)
				5	-0.05 (-3.25, 3.1)
					6

TNF-α					
CON	RT	ART	PMT	ACT	AT
1	1.35 (-0.62, 3.28)	-0.87 (-1.71, 0.01)	-0.28 (-2.8, 2.32)	-0.02 (-1.83, 1.81)	-0.62 (-1.47, 0.02)
	2	-2.23 (-4.19, -0.18)	-1.63 (-4.81, 1.63)	-1.37 (-4.01, 1.32)	-1.98 (-4.02, -0.04)
		3	0.59 (-2.08, 3.3)	0.85 (-1.18, 2.84)	0.25 (-0.93, 1.18)
			4	0.26 (-2.88, 3.36)	-0.35 (-3.12, 2.21)
				5	-0.59 (-2.68, 1.27)
					6

A total of 85 RCTs (69 two-arm, 11 three-arm, and 5 four-arm) (24–26, 28, 32, 34, 35, 37, 38, 40–42, 47, 49–53, 56–60, 62–65) reported improvements in HDL-C outcome indicators for patients with T2DM and overweight or obesity. The network meta-analysis results showed that in terms of improving HDL-C outcome indicators, the effects of ART, PMT, and AT were better than those of the control group as shown in **Figure 4D**. The SUCRA ranking results showed: PMT (0.8063) > AT (0.7139) > ART (0.6559) > WBT (0.5017) > RT (0.3948) > ACT (0.3138) > CON (0.1137). PMT was most likely to be the best intervention measure as shown in **Table 3**.

A total of 76 RCTs (62 two-arm, 10 three-arm, and 4 four-arm) (24–26, 32, 34, 38, 40–42, 47, 49, 51–53, 56, 58–60, 62–65, 67–75, 77–85, 87–90, 92–103, 121–124) reported improvements in LDL-C outcome indicators for patients with T2DM and overweight or obesity. The network meta-analysis results showed that in terms of improving LDL-C outcome indicators, the effects of PMT and AT were better than those of the control group as shown in **Figure 4E**. The SUCRA ranking results showed: PMT (0.9671) > AT (0.6356) > WBT (0.5606) > RT (0.4527) > ART (0.4034) > ACT (0.33) > CON (0.1505). PMT was most likely to be the best intervention measure as shown in **Table 3**.

A total of 92 RCTs (75 two-arm, 11 three-arm, and 6 four-arm) (24–36, 38–43, 46, 48, 49, 52–54, 56–59, 61, 63, 64, 66–70, 73, 75–83, 121, 125–132) reported improvements in FBG outcome indicators for patients with T2DM and overweight or obesity. The network meta-analysis results showed that in terms of improving FBG outcome indicators, the effects of RT, PMT, and AT were better than those of the control group as shown in **Figure 4F**. The SUCRA ranking results showed: PMT (0.8552) > AT (0.6787) > ACT (0.6731) > RT (0.5693) > ART (0.3461) > WBT (0.277) > CON (0.1007). PMT was most likely to be the best intervention measure as shown in **Table 4**.

**Table 4:** SUCRA ranking league.

BMI						
CON	RT	ART	PMT	ACT	AT	WBT
0.0867	0.6159	0.9332	0.2907	0.7064	0.4404	0.4267

TG						
0.1346	0.3842	0.5952	0.8277	0.443	0.3841	0.7313

TC						
0.0794	0.5966	0.5571	0.8019	0.6463	0.4116	0.407

HDL-C						
0.1137	0.3948	0.6559	0.8063	0.3138	0.7139	0.5017

LDL-C						
0.1505	0.4527	0.4034	0.9671	0.33	0.6356	0.5606

FBG						
0.1007	0.5693	0.3461	0.8552	0.6731	0.6787	0.277

HbA1c%						
0.0652	0.4471	0.6293	0.8171	0.478	0.7809	0.2823

HOMA-IR						
0.0466	0.3906	0.795	0.8473	0.6584	0.3662	0.396

IL-6						
0.3217	0.0716	0.8574	0.8405	0.4459	0.463	

TNF-α						
0.3743	0.0812	0.8329	0.5459	0.4399	0.7258	

A total of 90 RCTs (74 two-arm, 10 three-arm, and 6 four-arm) (24, 25, 27, 29, 30, 33–35, 37, 38, 41–43, 46, 47, 49–54, 56, 57, 59, 61, 62, 64, 67–70, 73–89, 122, 125, 126, 128–130, 132–135) reported improvements in HbA1c% outcome indicators for patients with T2DM and overweight or obesity. The network meta-analysis results showed that in terms of improving HbA1c% outcome indicators, the effects of RT, ART, PMT, and AT were better than those of the control group as shown in **Figure 4G**. The SUCRA ranking results showed: PMT (0.8171) > AT (0.7809) > ART (0.6293) > ACT (0.478) > RT (0.4471) > WBT (0.2823) > CON (0.0652). PMT was most likely to be the best intervention measure as shown in **Table 4**.

A total of 41 RCTs (34 two-arm, 3 three-arm, and 4 four-arm) (25–27, 40, 41, 43, 45, 48, 53–57, 61, 63, 66, 70, 74, 76, 78–80, 83, 86, 88, 94, 103–105, 112–114, 117, 118, 121, 123, 127, 131, 136–138) reported improvements in HOMA-IR outcome indicators for patients with T2DM and overweight or obesity. The network meta-analysis results showed that in terms of improving HOMA-IR outcome indicators, the effects of RT, ART, PMT, ACT, and AT were better than those of the control group as shown in **Figure 4H**. The SUCRA ranking results showed: PMT (0.8473) > ART (0.795) > ACT (0.6584) > WBT (0.396) > RT (0.3906) > AT (0.3662) > CON (0.0466). PMT was most likely to be the best intervention measure as shown in **Table 4**.

#### Inflammatory factors

A total of 18 RCTs (15 two-arm, 2 three-arm, and 1 four-arm) (33, 44, 49, 54, 66, 68, 76, 83, 93, 97, 103, 109, 110, 121, 126, 139–141) reported improvements in IL-6 outcome indicators for patients with T2DM and overweight or obesity. The network meta-analysis results showed that the ART group had better improvements in IL-6 outcome indicators compared to the control group as shown in **Figure 4I**. The SUCRA ranking results showed: ART (0.8574) > PMT (0.8405) > AT (0.463) > ACT (0.4459) > CON (0.3217) > RT (0.0716). ART was most likely to be the best intervention measure as shown in **Table 4**.

A total of 14 RCTs (13 two-arm and 1 four-arm) (33, 44, 49, 64, 66, 76, 81, 83, 91, 93, 103, 109, 121, 140) reported improvements in TNF-α outcome indicators for patients with T2DM and overweight or obesity. The network meta-analysis results showed that in terms of improving TNF-α outcome indicators, the crude improvement effect of no intervention was better than that of the control group as shown in **Figure 4J**. The SUCRA ranking results showed: ART (0.8329) > AT (0.7258) > PMT (0.5459) > ACT (0.4399) > CON (0.3743) > RT (0.0812). ART was most likely to be the best intervention measure as shown in **Table 4**.

The network meta-analysis SUCRA league table is presented in **Table 4**.

### Meta-regression analysis

**Figure 5:**
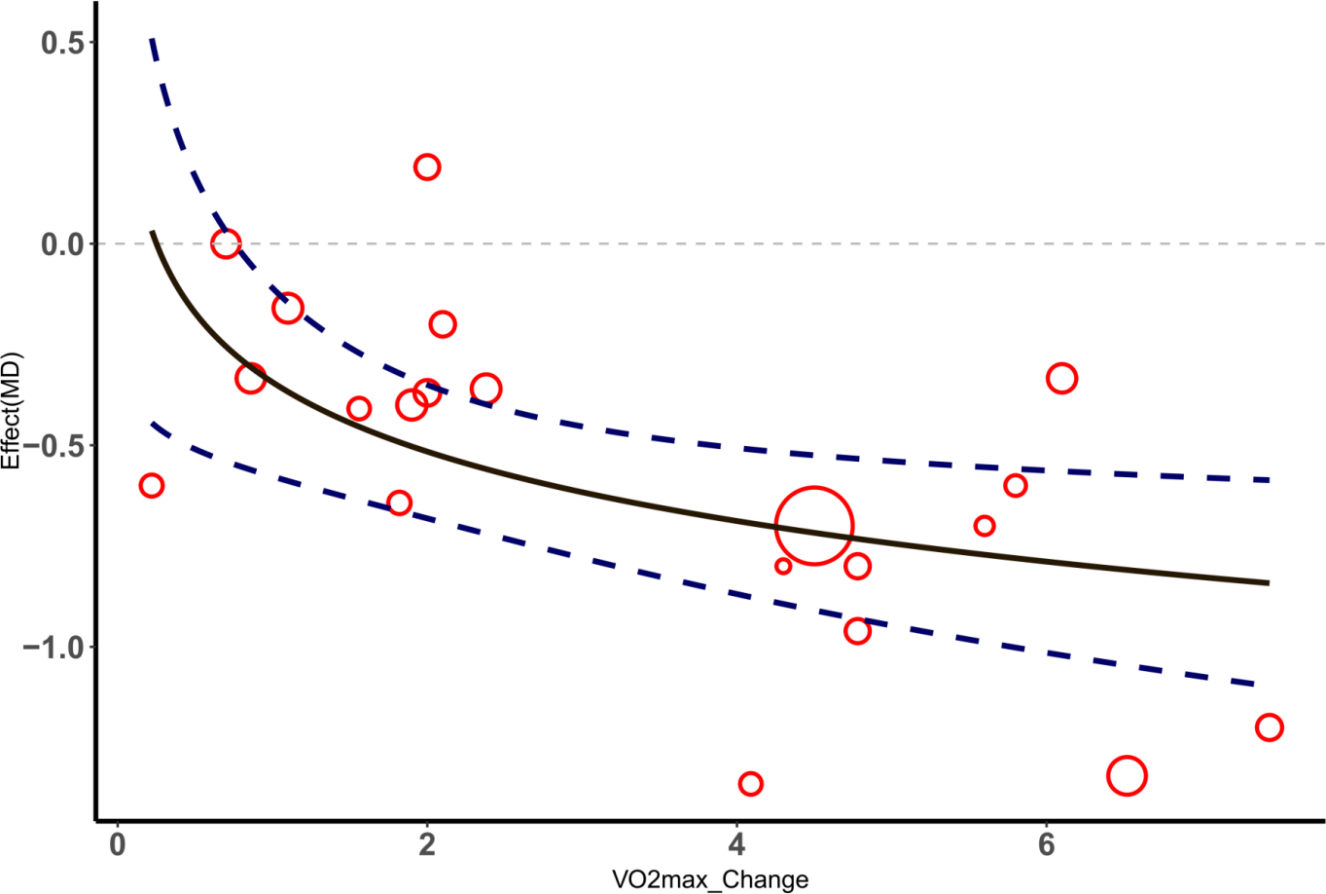
△ Meta-regression analysis of VO_2max_ and HbA1c%.

**Figure 6:**
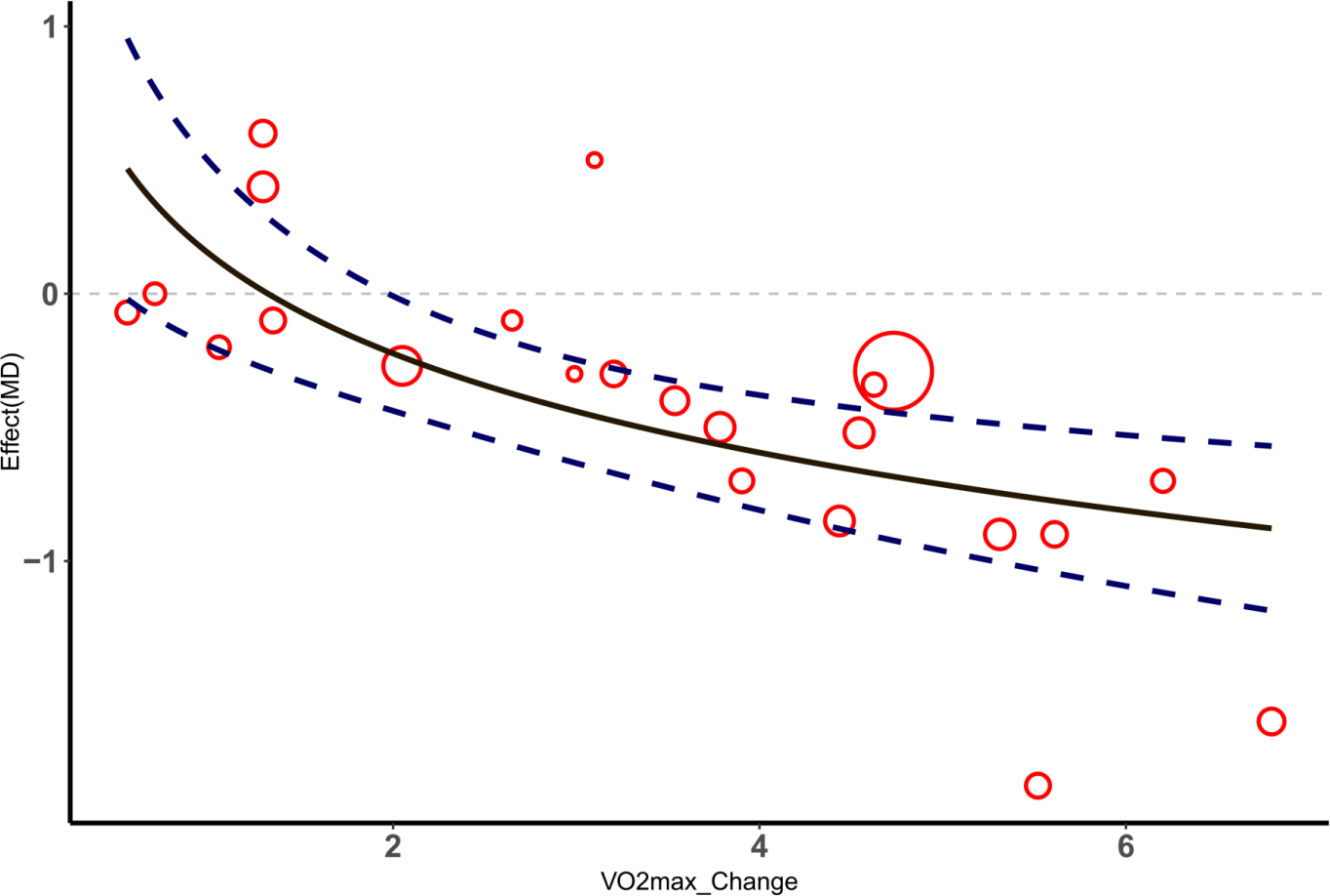
△ Meta-regression analysis of VO_2max_ and HbA1c%.

According to the included studies, the change in VO_2max_ significantly impacted the effect size of the selected outcome measures. The nonlinear relationship between the change in VO_2max_ and the effect size of outcome indicators was better captured using a logarithm-transformed regression model. In the meta-regression analysis, the BMI effect size was used as the dependent variable, and the VO_2max_ change value was used as the independent variable. The results showed an R-squared value of 0.4405 and an adjusted R-squared value of 0.4138, indicating that the model explained approximately 44.05% of the BMI change. The F-test results (F=16.53, df=1 and 21, P=0.0005551) indicated that the model was overall significant. For each unit increase in the logarithmic change in VO_2max_, BMI decreased by 0.5350 units (P< 0.001). Similarly, a meta-regression analysis was performed using the HbA1c% effect size as the dependent variable, and the VO_2max_ change value was used as the independent variable. The results showed an R-squared value of 0.2954 and an adjusted R-squared value of 0.2584, indicating that the model explained approximately 29.54% of the HbA1c% change. The F-test results (F=7.967, df=1 and 19, P=0.01087) indicated that the model was overall significant. For each unit increase in the logarithmic change in VO_2max_, HbA1c% decreased by 0.24834 units (P< 0.05), as shown in **Figures 5**, **6**.

### Two-dimensional graph evaluation

**Figure 7:**
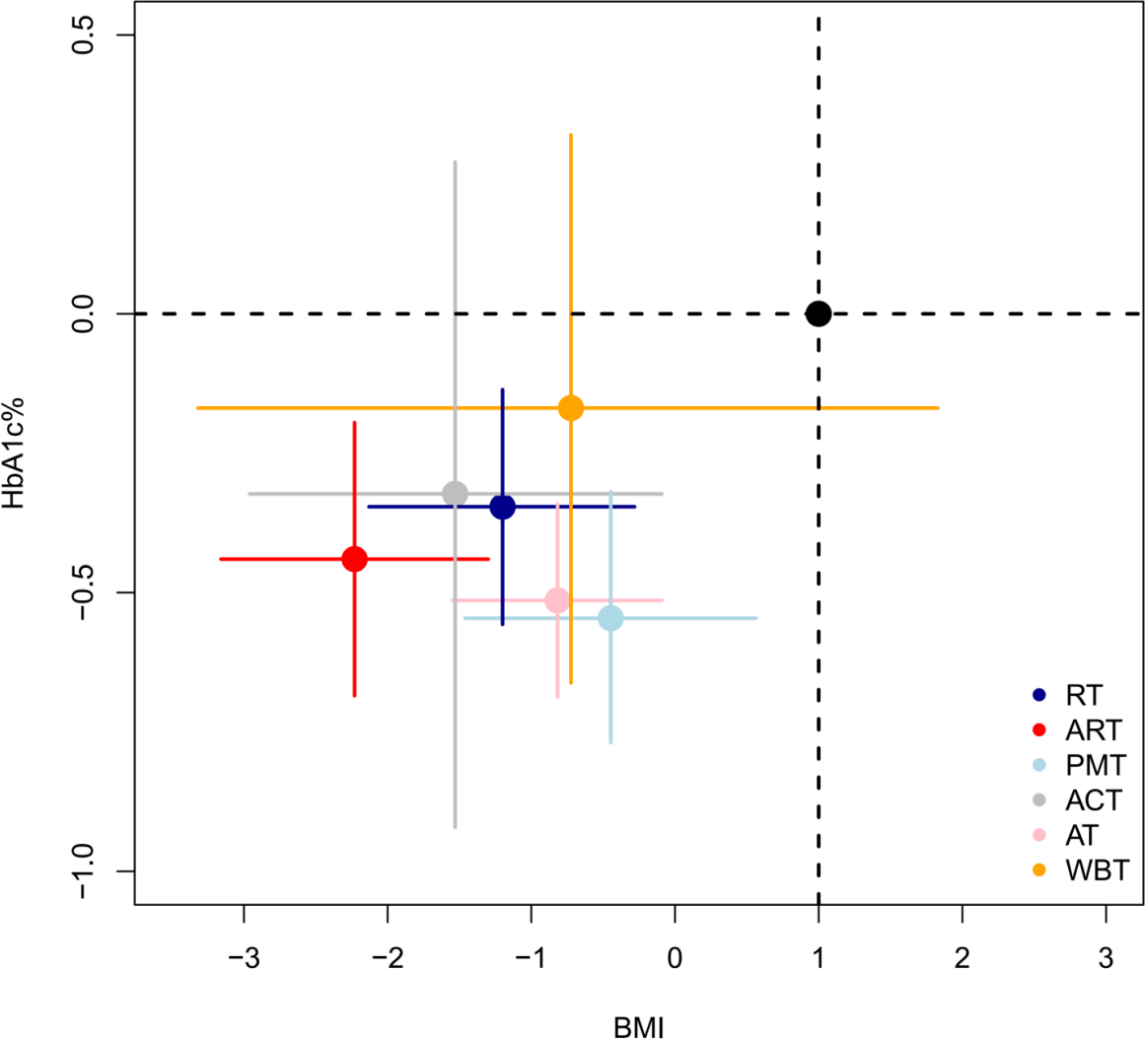
Two-dimensional evaluation of the comprehensive intervention effects of different non-drug interventions on BMI and HbA1c%.

**Figure 8:**
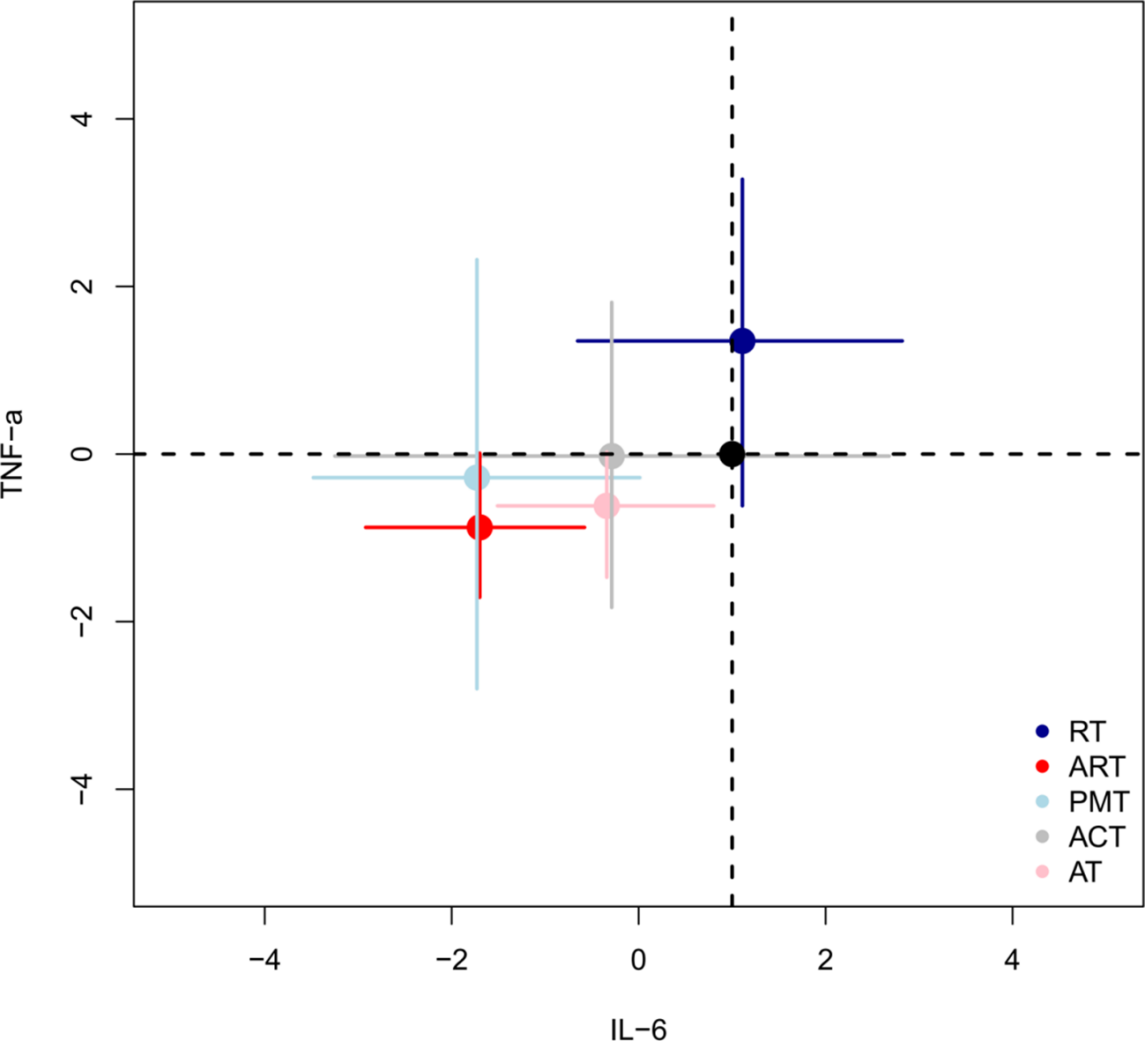
Two-dimensional evaluation of the comprehensive intervention effects of different non-drug interventions on IL-6 and TNF-α.

This study used two-dimensional plots to evaluate the comprehensive effects of different intervention measures on BMI, HbA1c%, IL-6, and TNF-α. **Figure 7** shows the performance of different intervention measures on the outcome indicators of BMI and HbA1c%. **Figure 8** shows the performance of different intervention measures on the outcome indicators of IL-6 and TNF-α. In the figures, each intervention is represented by a point of a different color. The position of the point indicates the impact of the intervention on the corresponding indicator, and the error bars indicate its 95% confidence interval. The analysis results in **Figures 6** and **7** show that the ART intervention had the best effect on the four selected outcome indicators. Specifically, the ART point is close to the lower left corner in both **Figures 7** and **8**, indicating that it can significantly reduce the levels of BMI, HbA1c%, IL-6, and TNF-α with a better effect than other interventions. In summary, through the comprehensive evaluation of the two-dimensional graphs, the ART intervention demonstrated the best performance in reducing BMI, HbA1c%, IL-6, and TNF-α, and it is recommended to prioritize this intervention strategy.

### Publication bias assessment

The adjusted funnel plots for publication bias of the 10 outcome indicators are presented in **Figures 9**-**18**. The adjusted funnel plots for the studies included in the 10 outcome indicators are relatively symmetrical, with fewer studies falling at the bottom of the funnel. This indicates that there may be a small sample effect or a certain degree of publication bias.

**Figure 9:**
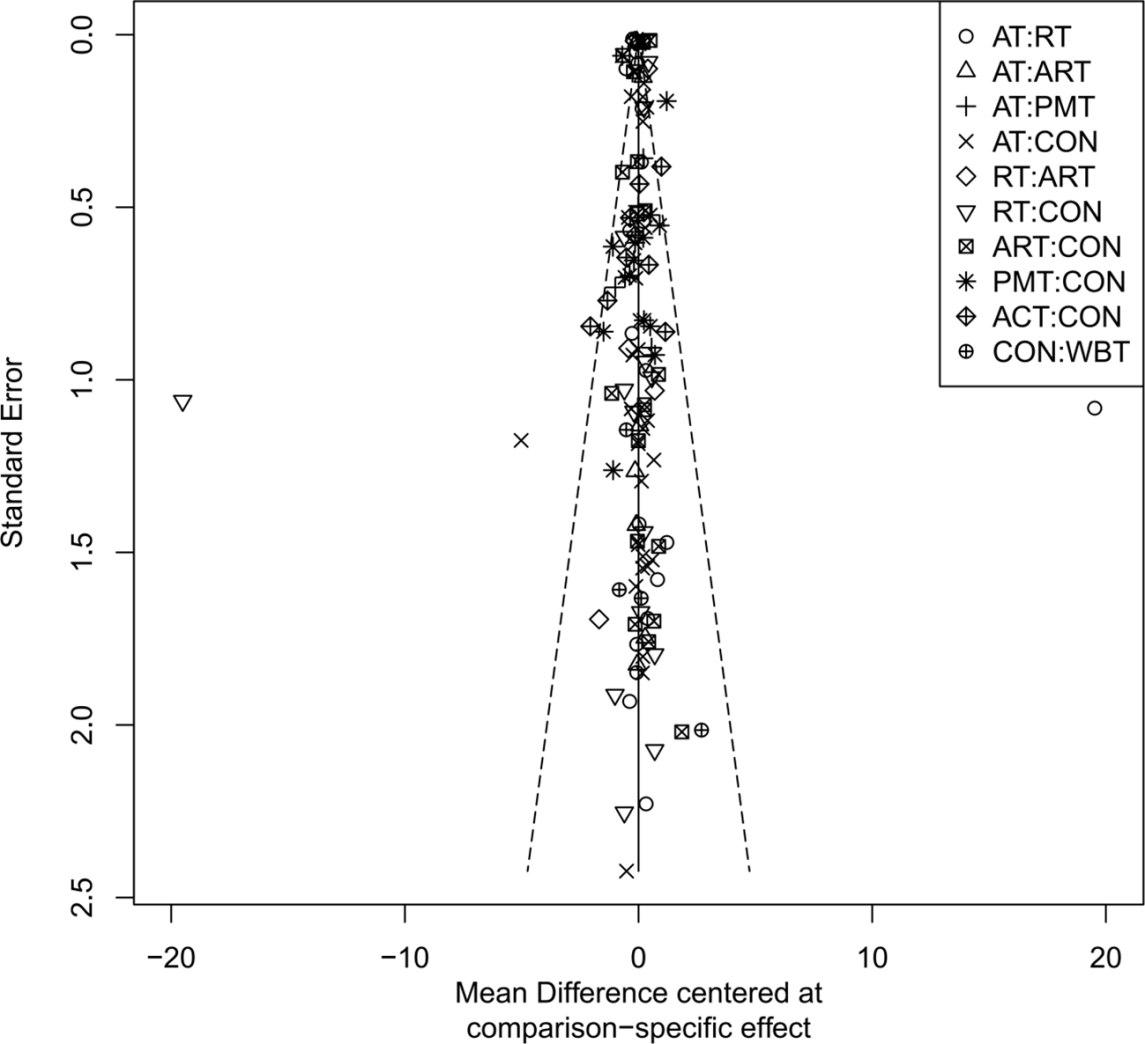
Comparison-correction funnel diagram (BMI).

**Figure 10:**
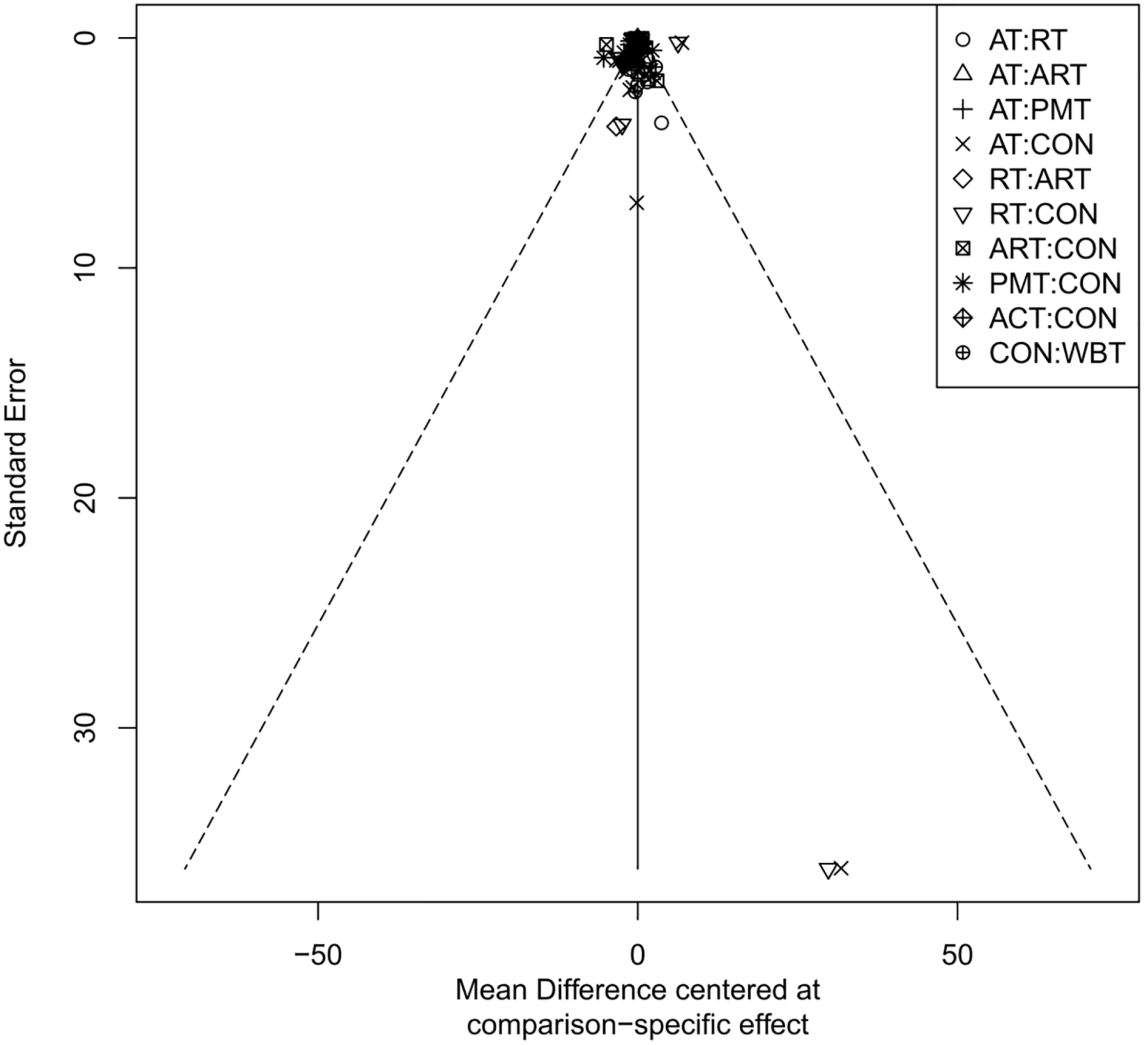
Comparison-correction funnel diagram (TG).

**Figure 11:**
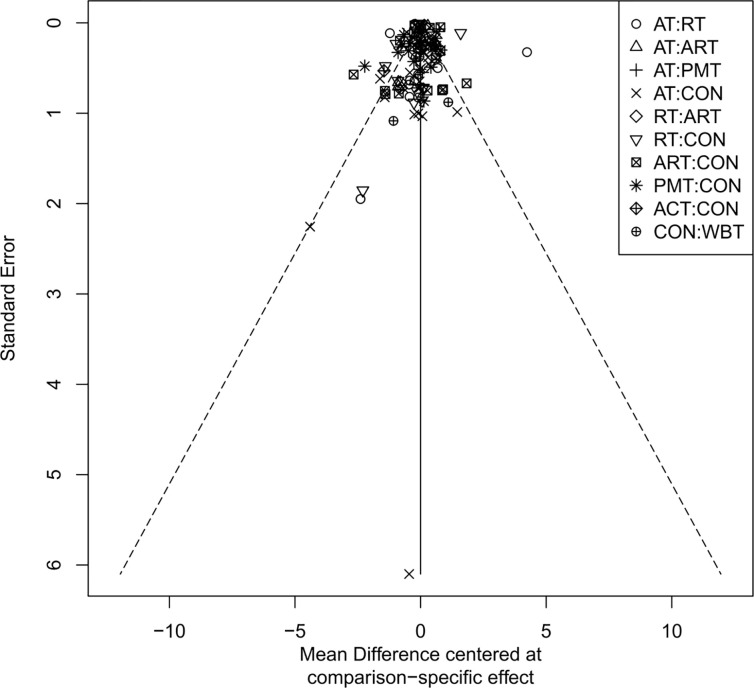
Comparison-correction funnel diagram (TC).

**Figure 12:**
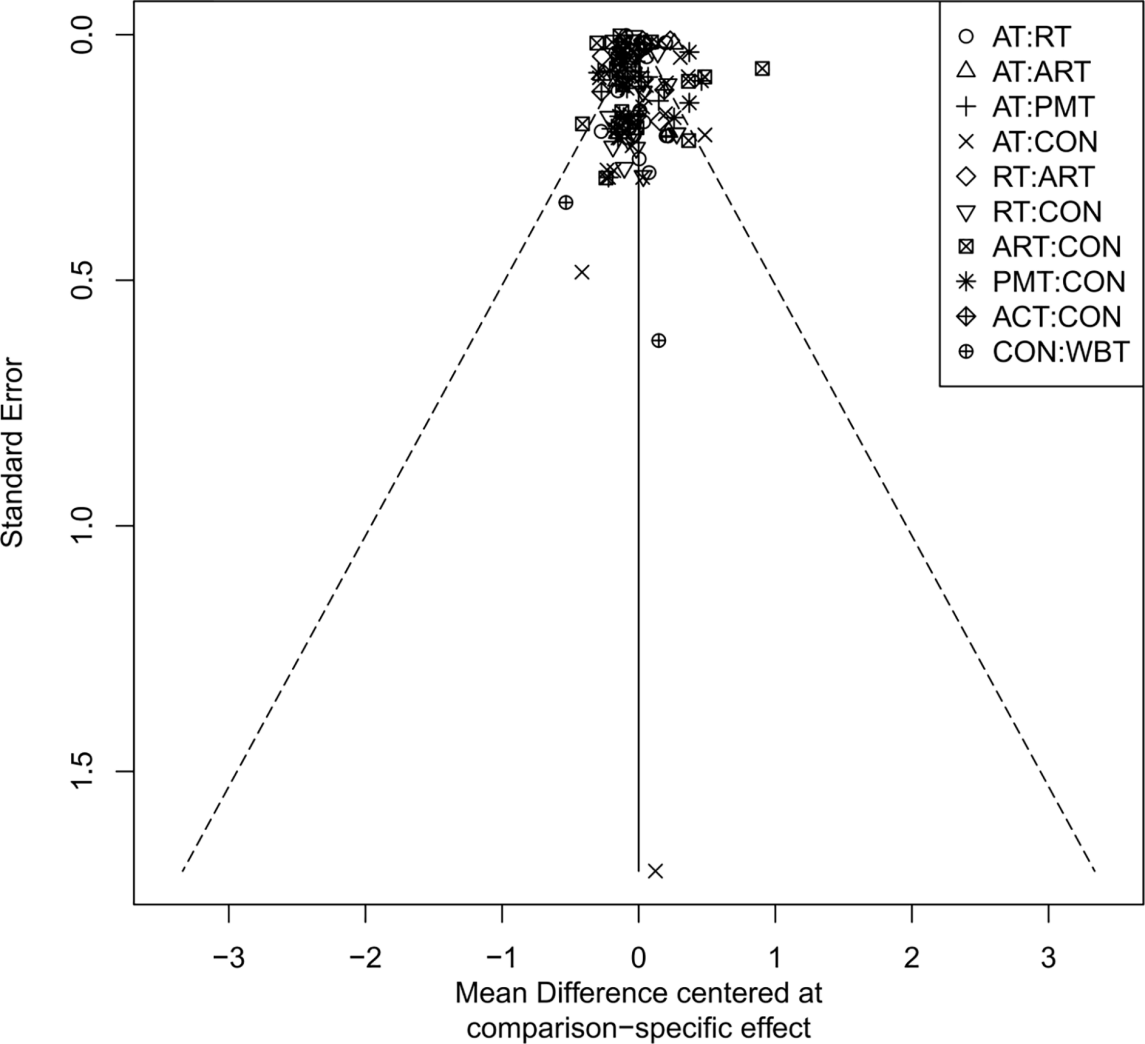
Comparison-Correction Funnel Diagram (HDL-C).

**Figure 13:**
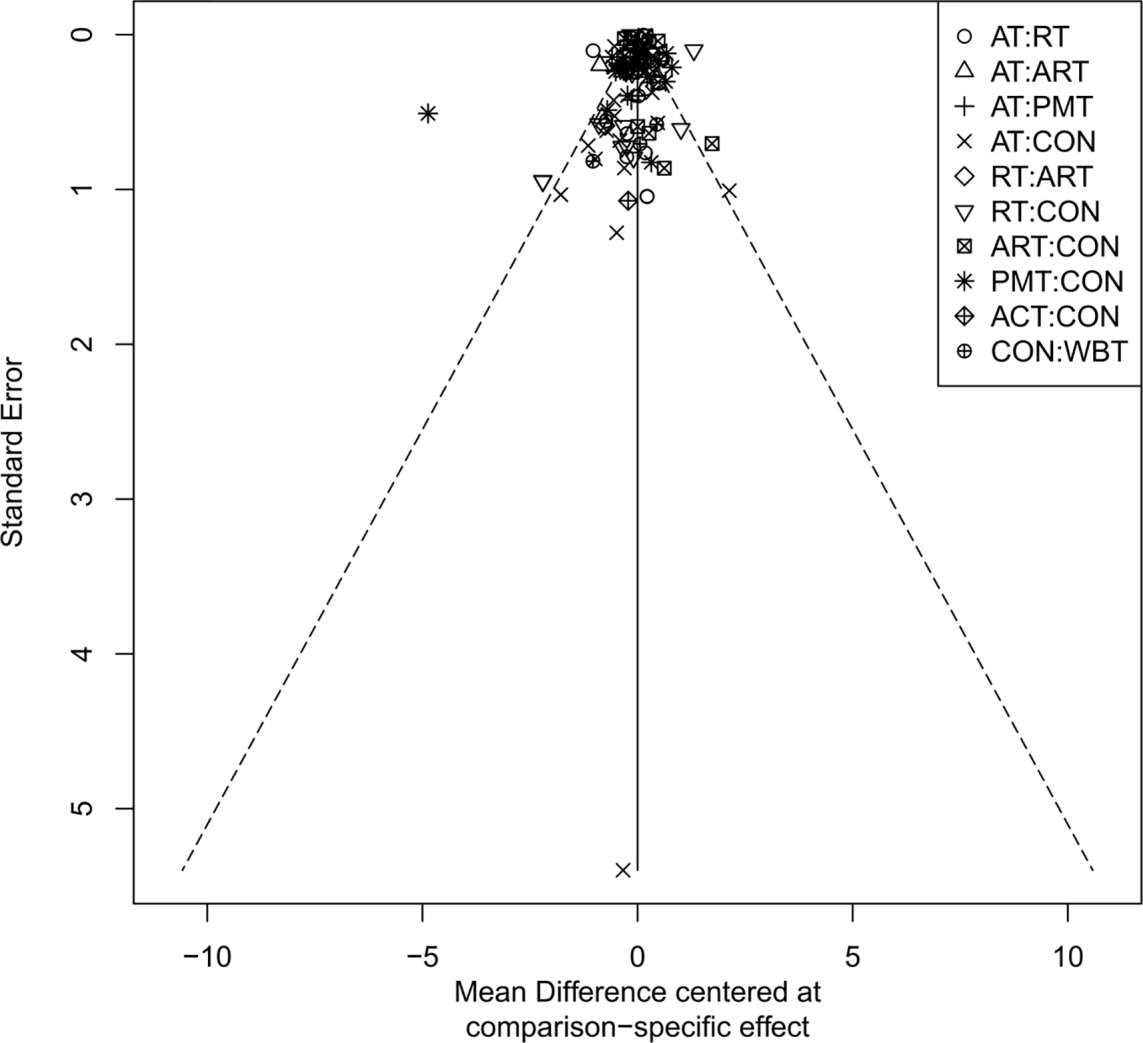
Comparison-correction funnel diagram (LDL-C).

**Figure 14:**
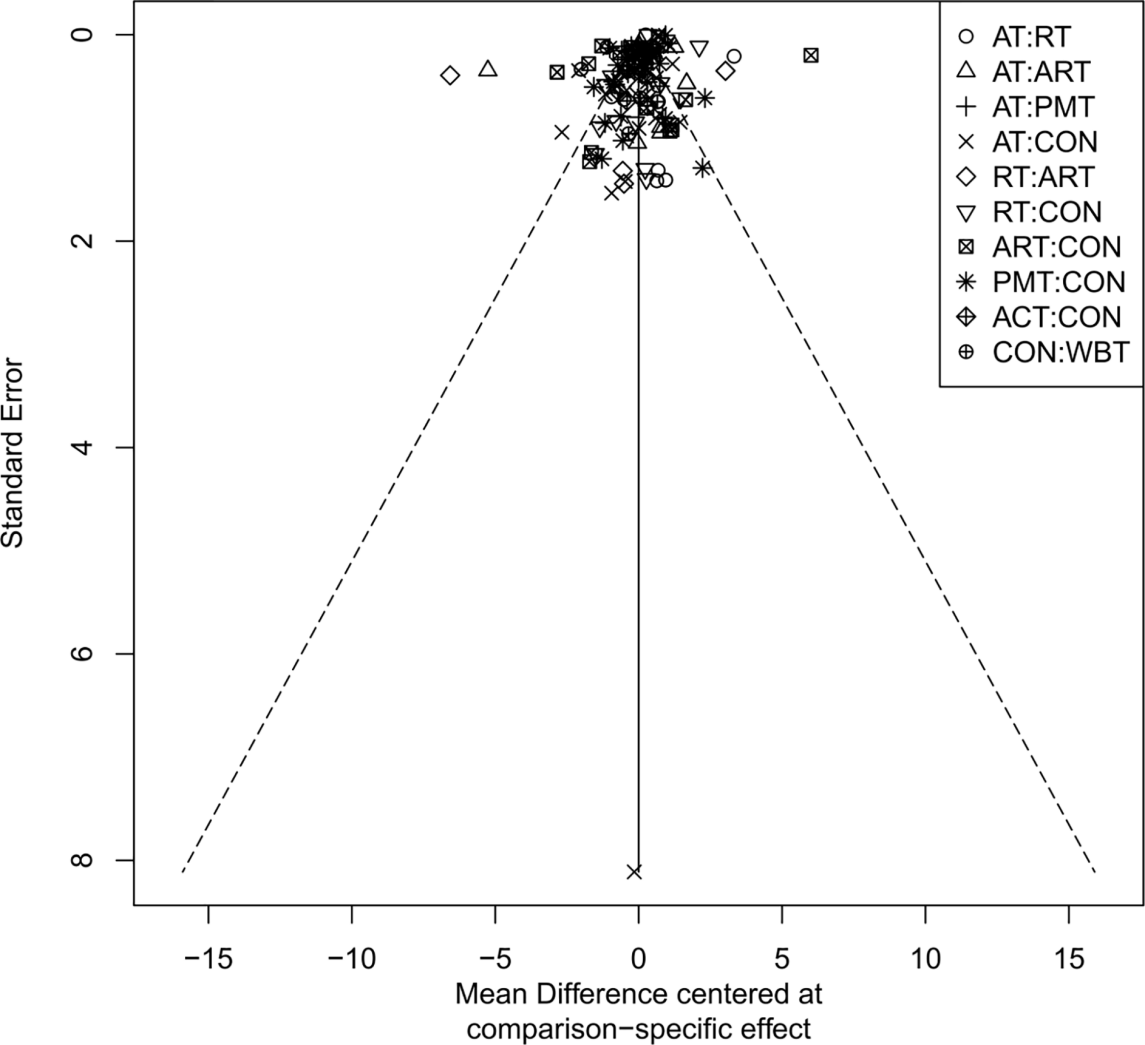
Comparison-correction funnel diagram (FBG).

**Figure 15:**
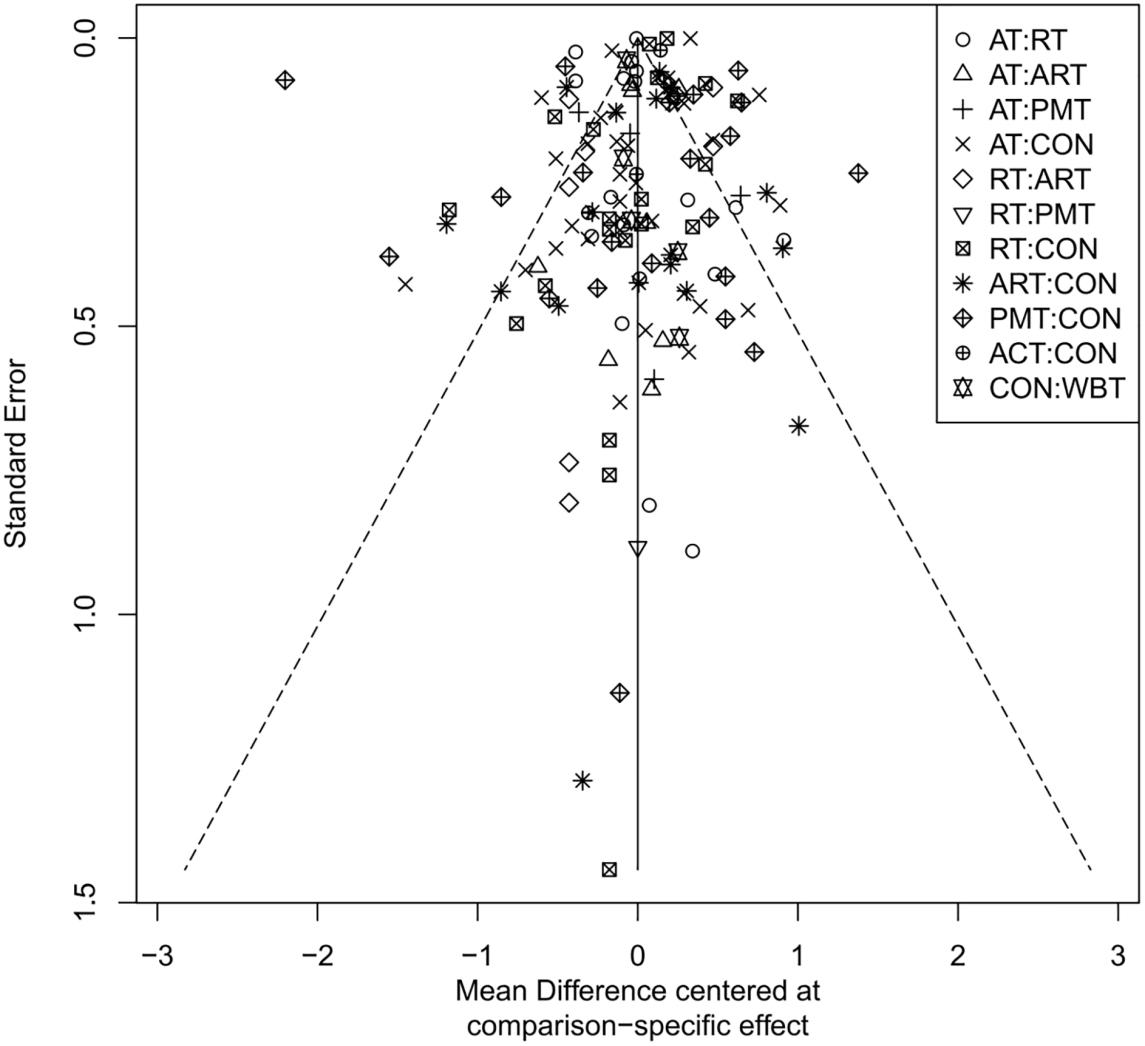
Comparison-correction funnel diagram (HbA1c%).

**Figure 16:**
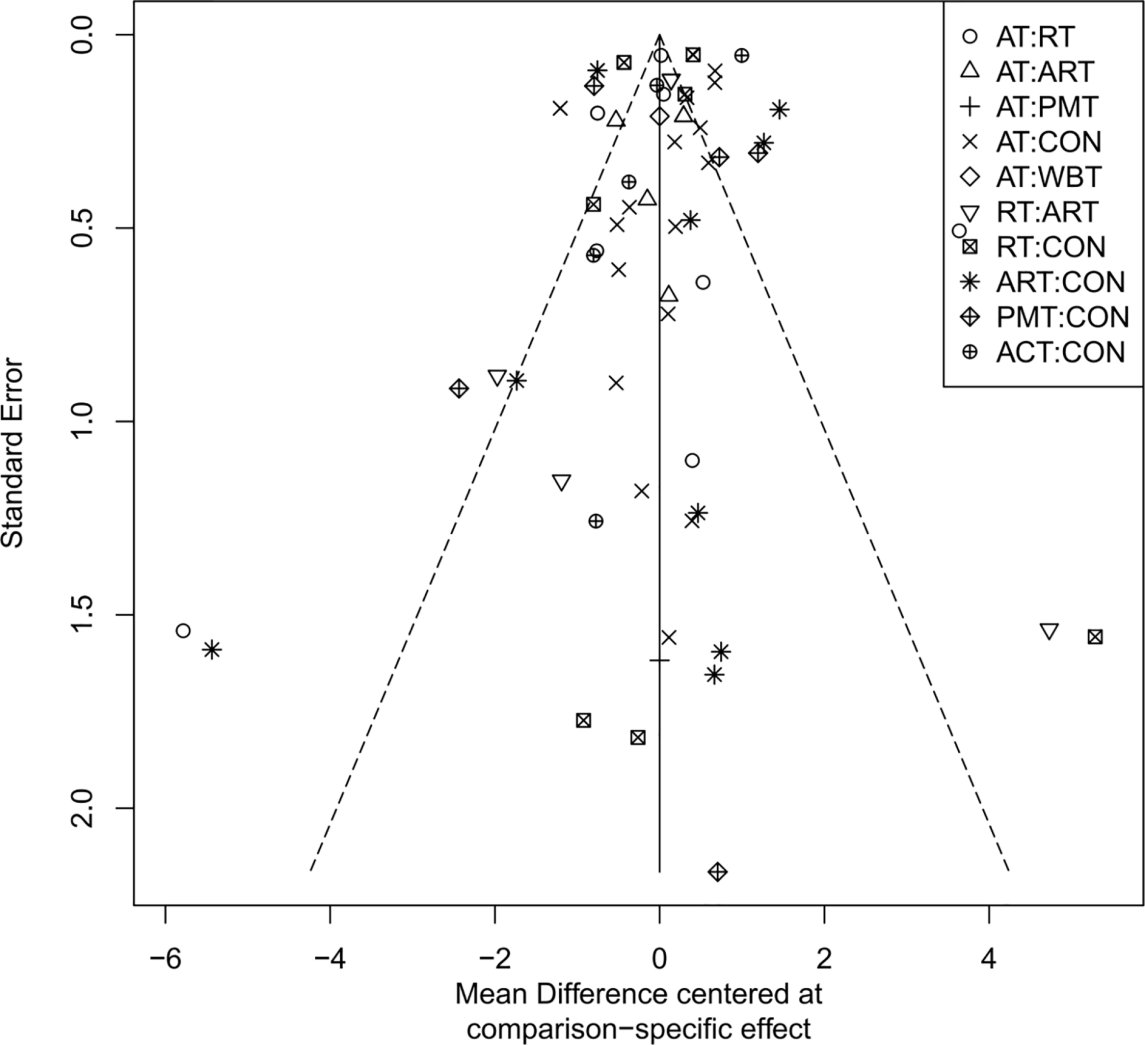
Comparison-correction funnel diagram (HOMA-IR).

**Figure 17:**
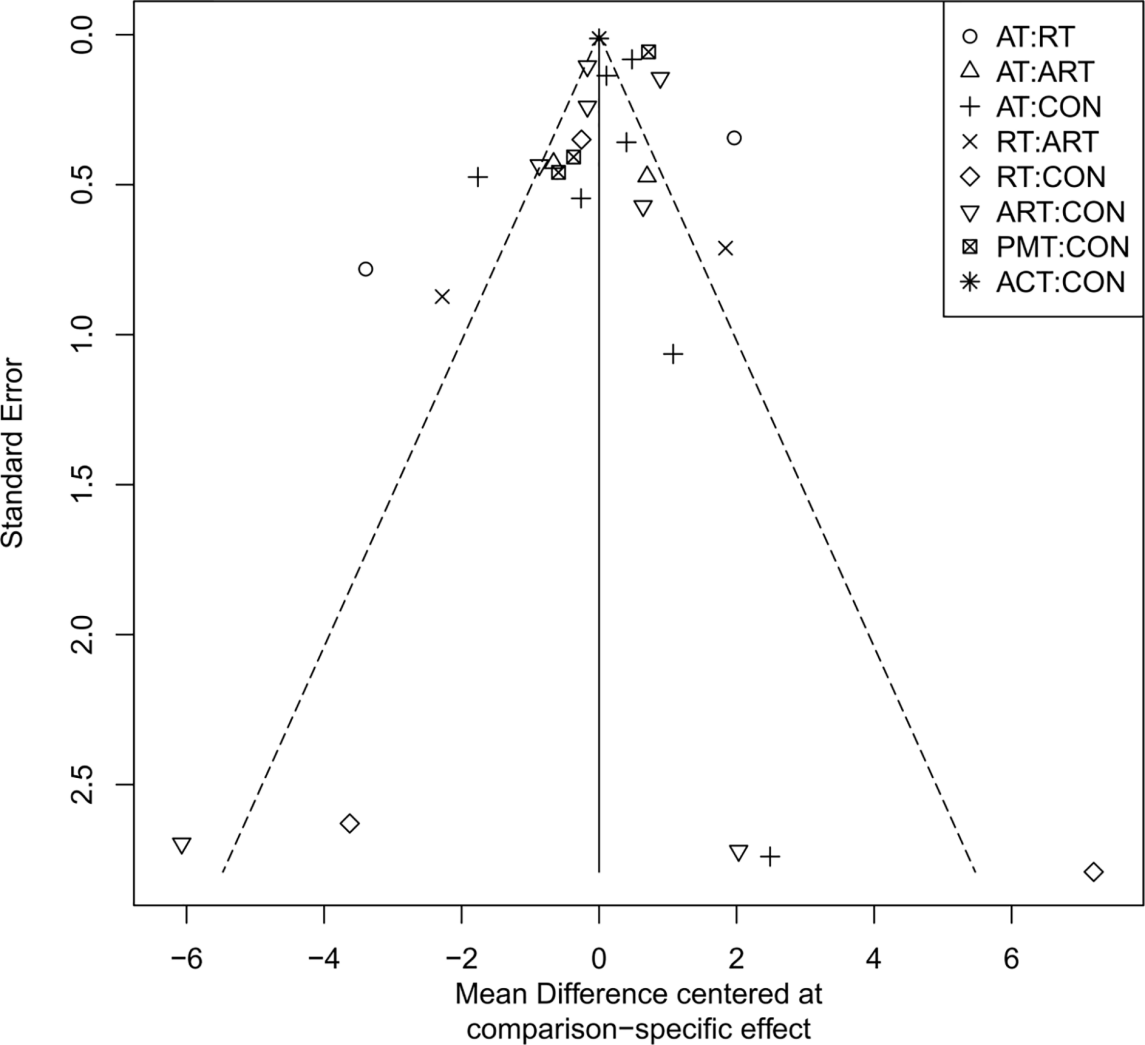
Comparison-correction funnel diagram (IL-6).

**Figure 18:**
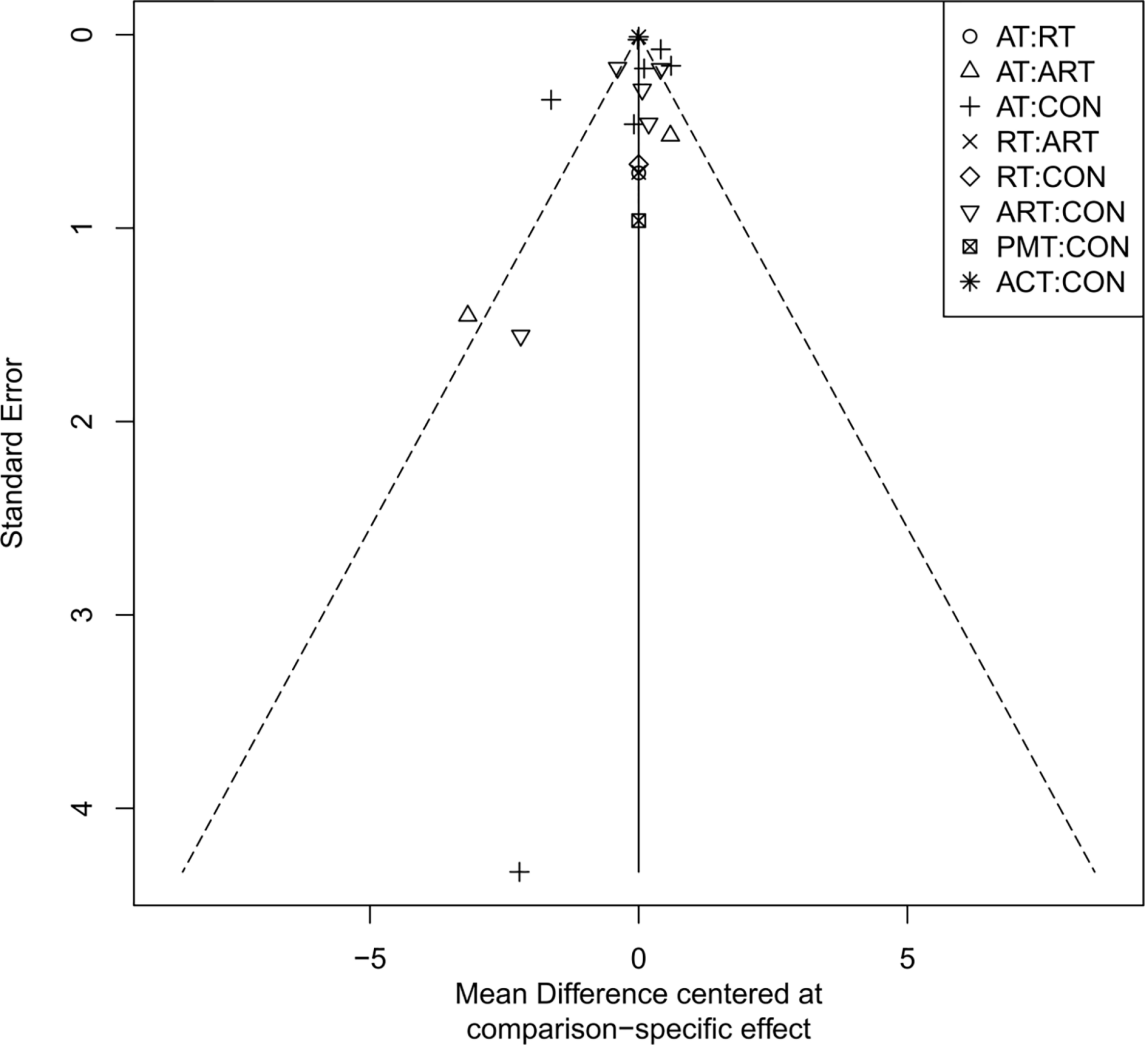
Comparison-correction funnel diagram (TNF-α).

## Discussion

In 1988, Professor Reaven of Stanford University first elucidated the pathogenesis of obesity-related T2DM (142). In overweight or obese individuals, adipose tissue, muscle tissue, and the liver become less sensitive to insulin, causing pancreatic beta cells to secrete more insulin to maintain blood sugar balance (143). If this compensatory hyperinsulinemia persists for a long time, it causes pancreatic β-cell function to gradually decline, reduces insulin secretion, and fails to effectively control blood sugar levels, eventually leading to diabetes (144). With the deepening of research, various theories have been proposed, including the inflammation hypothesis (145), the seborrheic hypothesis, the adipokine hypothesis (146), Gut flora hypothesis (147).

The inflammation hypothesis suggests that obesity is a chronic inflammatory state, and inflammatory factors such as TNF-α, interleukin-1β (IL-1β), and IL-6 can decrease tissue sensitivity to insulin and lead to pathological changes in pancreatic β-cell function (145). The lipid overflow hypothesis posits that when the storage capacity of adipose tissue reaches its limit, FFA overflow and accumulate in non-adipose tissues such as the liver, skeletal muscle, and pancreas, leading to IR and damage to pancreatic β-cell function. The adipokine hypothesis suggests that in obesity, the pattern of adipokines secreted by adipose tissue changes, with an increase in pro-inflammatory factors and a decrease in anti-inflammatory factors, leading to chronic low-grade inflammation and IR (146). The gut microbiota hypothesis suggests that the intestinal microbiota plays an important role in the pathological processes of obesity and T2DM by affecting energy balance, metabolic status, and inflammation levels (147).

The trend of obesity and T2DM among younger individuals is increasingly apparent, which may be closely related to high-calorie diets, sedentary lifestyles, and lack of exercise (148). Based on the above mechanisms, intervention measures for obesity and T2DM have gradually diversified and become increasingly refined. Among them, combined interventions such as AT, RT, ART, PMT, ACT, and WBT have shown significant results in various outcome indicators. This article aims to evaluate the effects of these interventions on body composition, blood lipid metabolism, and inflammatory factors in obese patients with T2DM through a systematic review and network meta-analysis and to provide a scientific basis for optimizing clinical intervention strategies.

### Body composition

In patients with T2DM and obesity, changes in body fat and muscle composition are directly related to disease progression and management. Through network meta-analysis, this article found that ART had the most significant effect in improving body composition, but AT, RT, and ACT also showed significant improvements. ART promotes lipolysis and inhibits lipogenesis in adipocytes via multiple pathways, effectively reducing body fat content. Initially, ART enhances the activity of hormone-sensitive lipase (HSL), a crucial enzyme in lipolysis. Elevated HSL activity facilitates the hydrolysis of triacylglycerol, thereby releasing free fatty acids (FFA) and glycerol. Catecholamines, such as epinephrine and norepinephrine, regulate this process by activating adenylyl cyclase (AC) via β-adrenergic receptors, thus increasing intracellular cyclic adenosine monophosphate (cAMP) levels, activating protein kinase A (PKA), and ultimately enhancing HSL phosphorylation and activation (149, 150). Moreover, combined aerobic and resistance exercise significantly enhances HSL by boosting its protein content and enzymatic activity in adipose tissue, further facilitating lipolysis. During exercise, phosphorylation of HSL at sites such as Ser-563 and Ser-660 increases, enhancing its translocation to lipid droplets and augmenting its lipolytic activity. Additionally, exercise facilitates the interaction between HSL and the lipid droplet-associated protein PLIN1, thereby enhancing HSL’s access to triacylglycerol and diacylglycerol substrates within the droplets (151). In addition, ART inhibits the expression and activity of key enzymes involved in lipogenesis in adipose tissue, such as fatty acid synthase (FAS) and acetyl-CoA carboxylase (ACC). Through the AMP-activated protein kinase (AMPK) pathway, ART reduces the expression of sterol regulatory element-binding protein 1c (SREBP-1c) (152), a key regulatory factor in fat synthesis, and decreases the synthesis of nascent fatty acids (153). ART not only improves body composition by reducing the amount of adipose tissue but also optimizes body composition by promoting muscle protein synthesis and enhancing muscle metabolism (154). PGC-1α promotes mitochondrial DNA replication and protein expression by interacting with transcription factors such as nuclear respiratory factor 1 (NRF-1) and mitochondrial transcription factor A (TFAM). ART promotes β-oxidation of fatty acids and increases energy consumption by increasing the activity of fatty acid oxidases such as carnitine palmitoyltransferase 1 (CPT1) in skeletal muscle. The AMPK pathway plays an important role in this process. ART activates AMPK, inhibits the activity of ACC, and reduces the production of malonyl-coenzyme A (malonyl-CoA), thereby releasing the inhibition of CPT1 and promoting fatty acids to enter the mitochondria for oxidative metabolism (155). Additionally, AT and RT also show significant effects in improving body fat composition. AT reduces body fat by increasing whole-body energy consumption and fat oxidation, enhancing the number and function of mitochondria in skeletal muscle cells, and improving fatty acid oxidation capacity in muscles. Additionally, AT positively impacts body fat composition by increasing cardiopulmonary function and improving systemic metabolic status. RT improves the quality of skeletal muscle by increasing the volume and number of muscle fibers and promoting muscle protein synthesis (156). Additionally, RT helps reduce body fat by increasing BMR and resting energy expenditure and improves glucose metabolism by increasing insulin sensitivity (157). ACT, as a traditional Chinese medicine method, has also shown certain effects in improving body composition. ACT helps reduce body fat by regulating the neuroendocrine system and promoting lipolysis and energy metabolism. ACT also regulates body composition by improving insulin sensitivity and regulating the secretory function of adipocytes (158). Additionally, ART further affects body composition by regulating hormone levels. ART significantly improves insulin sensitivity and reduces IR. Its mechanism includes increasing the expression and activity of insulin receptor substrate 1 (IRS-1) and glucose transporter type 4 (GLUT4) in skeletal muscle and adipose tissue, enhancing insulin signaling, thereby promoting glucose uptake and utilization. ART regulates leptin and adiponectin, two hormones secreted by adipose tissue, reducing leptin levels, decreasing leptin resistance, and increasing adiponectin levels. Adiponectin exerts anti-inflammatory and insulin-sensitizing effects through the AMPK pathway (159).

### Lipid metabolism

In patients with T2DM and overweight or obesity, dyslipidemia is closely related to IR and is a significant factor in disease progression. This study found through network meta-analysis that different types of training improve blood lipid metabolism, especially PMT, which showed significant improvements in multiple blood lipid metabolism indicators. PMT showed the best effect on multiple indicators such as TG, TC, HDL-C, LDL-C, FBG, HbA1c, and HOMA-IR, which may be related to its unique mechanisms. Recent research indicates that PMT has a positive impact on blood lipid metabolism by comprehensively regulating physical and mental status and improving endocrine and nervous system functions (111). PMT includes yoga, tai chi, and other practices. These exercises reduce the secretion of stress hormones (such as cortisol) and decrease the overactivity of the sympathetic nervous system, thereby improving insulin sensitivity. Research indicates that PMT can significantly increase the expression of IRS-1 and GLUT4, enhance glucose uptake and utilization, and reduce blood sugar and blood lipid levels (160). ART has a significant effect in reducing TG and LDL-C levels and increasing HDL-C levels mainly by increasing AMPK activity, promoting fatty acid oxidation and glucose uptake, and inhibiting FAS and ACC, thereby reducing the synthesis of nascent fatty acids. Additionally, ART contributes to long-term blood lipid management by enhancing muscle strength and metabolic rate and increasing resting energy expenditure (161). AT reduces body fat by increasing whole-body energy consumption and fat oxidation while improving cardiopulmonary function and systemic metabolic status. It also shows significant effects in reducing TG, TC, and LDL-C levels and increasing HDL-C levels. Recent research indicates that AT can improve insulin sensitivity and lipid metabolism by regulating intestinal flora and increasing the production of short-chain fatty acids (SCFA) (162). RT plays an important role in reducing FBG and HbA1c% levels by increasing muscle mass and metabolic rate, enhancing resting energy expenditure, and improving glucose metabolism. The mechanism of RT also includes increasing the number and function of mitochondria in skeletal muscle, promoting fatty acid oxidation and energy consumption, and reducing fat accumulation (68). ACT shows a certain lipid-lowering effect by regulating the neuroendocrine system, improving insulin sensitivity, and promoting adipocyte function. Emerging evidence suggests that acupuncture can enhance lipolysis and improve insulin sensitivity through several mechanisms. These include modulation of the hypothalamic-pituitary-adrenal (HPA) axis, reduction of chronic low-grade inflammation, and regulation of adipokine secretion such as adiponectin and leptin. Furthermore, stimulation of specific acupoints (e.g., ST36, CV12) has been shown to activate AMP-activated protein kinase (AMPK) and peroxisome proliferator-activated receptor gamma (PPAR-γ) signaling pathways, which play key roles in enhancing glucose uptake, fatty acid oxidation, and mitochondrial function. These effects collectively contribute to improved lipid metabolism and insulin action in individuals with obesity or type 2 diabetes (163). In summary, PMT has become the best intervention measure for multiple blood lipid metabolism indicators, which may be attributed to its multi-level and multi-target comprehensive regulatory effects. PMT shows unique advantages in blood lipid management by reducing stress hormone levels, regulating the neuroendocrine system, and improving insulin sensitivity and metabolic function (164). However, the advantages of other interventions such as ART, AT, and RT on specific indicators cannot be ignored. Different types of training methods improve blood lipid metabolism through various metabolic pathways and mechanisms, providing effective non-drug intervention methods for patients with T2DM and obesity. These interventions not only help reduce blood lipid levels but also reduce the risk of diabetes by improving insulin sensitivity and overall metabolic function, providing a comprehensive clinical management strategy.

### Inflammatory factors

In the pathogenesis of T2DM and obesity, inflammatory factors, particularly IL-6 and TNF-α, play a crucial role. In obesity and T2DM, the inflammatory response in adipose tissue increases significantly, mainly manifested by the infiltration of pro-inflammatory cytokines and immune cells, thereby accelerating the development of metabolic diseases (165). The results of the network meta-analysis showed that ART had a significant effect in reducing IL-6 levels, but the improvement effect on TNF-α did not reach significance.

Obesity significantly affects the immune system in adipose tissue, exacerbating the inflammatory response. This inflammatory environment attracts more immune cell infiltration, mainly macrophages and T lymphocytes, further aggravating the inflammatory response and inhibiting cellular metabolic function (166). Adipose tissue macrophages (ATMs) are crucial in the inflammatory process caused by obesity, with their infiltration promoting inflammation in adipose tissue. With the progression of obesity, the increase in pro-inflammatory signals prompts M2-type macrophages to transform into an M1-type pro-inflammatory phenotype, inducing adipocytes to secrete pro-inflammatory cytokines such as TNF-α, thereby promoting inflammatory responses and IR (13). This mechanism is particularly evident in patients with T2DM, as the inflammatory response not only aggravates IR but also worsens glycemic control by affecting pancreatic β-cell function. ART significantly reduces IL-6 levels through multiple mechanisms. First, ART enhances the expression of anti-inflammatory cytokines in adipose tissue and inhibits the secretion of IL-6, thereby reducing local and systemic inflammatory responses. Secondly, ART promotes the maintenance of M2 macrophages and the reduction of M1 macrophages in adipose tissue, a shift that helps alleviate the inflammatory response and improve insulin sensitivity (167). Additionally, ART enhances immune regulatory function by regulating the balance of T lymphocytes, reducing the infiltration of CD8+ T cells, and increasing the proportion of CD4+ T cells. At the molecular level, ART works by regulating multiple signaling pathways. ART significantly inhibits the activities of nuclear transcription factor kappa B (NF-κB) and c-Jun N-terminal kinase (JNK) signaling pathways. Inhibition of NF-κB and JNK signaling pathways reduces the expression of IL-6, thereby reducing the inflammatory response (168). Additionally, ART activates the phosphatidylinositol 3-kinase (PI3K)/Akt and AMPK signaling pathways. Activation of these pathways improves insulin sensitivity and enhances glucose and lipid metabolism, further alleviating IR (169). Although ART did not reach a significant difference in reducing TNF-α levels, the forest plot of the network meta-analysis still showed a trend in the improvement effect on TNF-α. This suggests that ART may have a regulatory effect on TNF-α levels, but it does not reach statistical significance. It is worth noting that the improvement of inflammatory factors is crucial to the overall metabolic health of T2DM patients. ART plays an important role in regulating blood sugar and improving metabolic function by reducing IL-6 levels and enhancing insulin sensitivity.

### Meta-regression analysis and two-dimensional graph evaluation

Meta-regression analysis results showed that changes in VO_2max_ significantly impacted the effect sizes of BMI and HbA1c%. Increases in VO_2max_ are closely related to improvements in BMI and HbA1c%. As an indicator of aerobic capacity, improvements in VO_2max_ mean enhanced cardiopulmonary function and improved systemic metabolic efficiency. Mechanistically, increasing VO_2max_ can increase the density and function of mitochondria in skeletal muscle, promote the oxidation of fatty acids and the uptake and utilization of glucose, directly leading to the reduction of body fat and stabilization of blood sugar (74). Therefore, the significant reduction effect of increasing VO_2max_on BMI and HbA1c% reflects its key role in metabolic regulation.

Two-dimensional graph evaluation further revealed the comprehensive advantages of ART across multiple outcome indicators. In the evaluation of the outcome indicators of BMI, HbA1c%, IL-6, and TNF-α, ART was the best intervention. This indicates that ART has a significant effect in comprehensively improving body composition, glycemic control, and inflammatory status. ART combines the advantages of AT and RT, achieving its therapeutic effects through a variety of molecular mechanisms.

First, ART effectively reduces BMI by activating AMPK and mTOR signaling pathways, increasing muscle synthesis and fat oxidation, increasing lean body mass, and reducing fat storage. Secondly, ART can significantly reduce HbA1c% levels and improve glucose metabolism by enhancing insulin sensitivity, promoting GLUT4 translocation and glucose uptake. Regarding the regulation of inflammatory factors, ART reduces the expression of pro-inflammatory factors and the levels of IL-6 and TNF-α by inhibiting the NF-κB and JNK signaling pathways. Modulation of these signaling pathways plays a key role in alleviating chronic inflammation and IR (153). Specifically, ART reduces the infiltration of M1 macrophages in adipose tissue and promotes the phenotype conversion of M2 macrophages, thereby reducing the inflammatory response. Additionally, ART regulates intestinal flora, increases the production of anti-inflammatory short-chain fatty acids (such as butyric acid), and further alleviates systemic inflammation (167). Moreover, both aerobic and resistance training induce the secretion of a wide range of exercise-induced signaling molecules known as exerkines, including myokines (e.g., irisin, IL-6), adipokines, and hepatokines. These circulating factors serve as mediators of inter-organ communication and play a crucial role in regulating metabolic function. Notably, exerkines influence distant target organs such as the liver, adipose tissue, and particularly the endocrine pancreas, where they enhance β-cell function and insulin secretion, ultimately contributing to improved glycemic control and systemic glucose homeostasis.

Overall, the results of meta-regression analysis and two-dimensional graph evaluation show that increasing VO_2max_ has a significant effect on improving BMI and HbA1c%, and ART shows comprehensive advantages across multiple metabolic and inflammatory indicators. ART employs multiple biomolecular mechanisms to comprehensively improve patients’ health status through metabolic regulation and inflammation relief, providing strong evidence for clinical intervention. Future research should further explore the individualized application of different training methods to optimize intervention strategies and maximize clinical effects.

### Precise exercise prescription

Based on the findings of our systematic review and network meta-analysis, a precise and comprehensive exercise prescription was developed to maximize metabolic improvements in patients with T2DM and obesity. The core components—ART, PMT, and ACT—were prioritized due to their high SUCRA rankings and superior effects across multiple outcomes, including BMI, HbA1c%, HOMA-IR, IL-6, and TNF-α. This was supported by multidimensional comparisons (**Figures 7**, **8**) and the consistency of their effects observed in subgroup analyses. To determine the specific frequency, intensity, and duration of each intervention, we examined the protocols of the most effective and methodologically rigorous RCTs included in our analysis. Aerobic training was commonly performed at 40–60% VO_2_;max for 30–60 minutes per session, 3 times per week, while resistance training was frequently conducted at 50–80% 1RM for similar durations and frequency. The structure of ART, combining moderate-intensity aerobic and resistance training within the same session, was drawn from protocols showing synergistic benefits on insulin sensitivity and body composition. PMT (e.g., Tai Chi, yoga) and ACT (e.g., body and auricular acupuncture targeting acupoints such as Zusanli, Sanyinjiao, Guanyuan, Pishu, and Weishu) were included based on trials demonstrating consistent benefits in inflammatory regulation and glycemic control. In addition, evidence from meta-regression analyses indicated that improvements in VO_2_;max were significantly associated with reductions in BMI and HbA1c%, further justifying the inclusion of structured aerobic components. Mechanistically, these interventions exerted beneficial effects via AMPK/mTOR and GLUT4 signaling, reduction of macrophage-driven inflammation, and modulation of gut microbiota and exerkines. Together, these findings informed the development of a multi-modal, evidence-based exercise plan tailored to the physiological needs of this population (**Table 5**).

**Table 5:** Recommendation of precise intervention prescriptions for type 2 diabetes and overweight and obesity.

Type	Frequency	Duration per Session	Intensity	Description
Aerobic Exercise (AE)	3 sessions per week	30–60 minutes	40% to 60% VO_2max_	e.g., running, cycling, swimming
Resistance Training (RT)	3 sessions per week	30–60 minutes	50% to 75%-80% 1RM	e.g., using dumbbells, barbells, gym equipment
Combined Resistance Training (ART)	3 sessions per week	60 minutes	Moderate-intensity AE 30 minutes + RT 30 minutes	Combination of aerobic and resistance training
Mind-Body Movement (PMT)	2–3 sessions per week	30–60 minutes	Low to Moderate	e.g., yoga, tai chi
Acupuncture (ACT)	2–3 sessions per week	30–60 minutes	NA	Commonly used body acupuncture and ear acupuncture; main acupoints: Zusanli, Sanyinjiao, Guanyuan, Pishu, Weishu

①It is recommended to exercise 1 hour after a meal, especially in the afternoon. ②Continuously monitor heart rate during exercise to ensure it remains within the target intensity range. ③Warm up and cool down for 10 minutes before and after each exercise session to prevent injuries. ④Regular health assessments should be conducted to adjust exercise intensity and methods, ensuring personalized intervention effects.NA, not available.

Although ACT is not a form of physical exercise in the traditional sense, it was included in the prescription due to its non-pharmacological nature and its complementary effects on metabolic regulation, inflammation, and insulin sensitivity, as demonstrated in our network meta-analysis. Thus, ACT was treated as a structured therapeutic session rather than an exercise modality, but integrated into the weekly schedule due to its evidence-based contribution to systemic metabolic improvements. We acknowledge that the combined frequency of the proposed interventions may appear intensive. However, the prescription is intended to offer flexible combinations tailored to individual schedules. Rather than requiring multiple sessions per day, participants are encouraged to alternate between modalities on different days or combine compatible types (e.g., ART replacing separate AT and RT sessions; PMT on rest days). The proposed format represents an optimal upper boundary based on available evidence, and clinicians are advised to adapt it to patient-specific contexts, considering time availability, occupational demands, and socioeconomic factors.

The recommendation to exercise approximately one hour after a meal, especially in the afternoon, is based on physiological and behavioral considerations. Postprandial exercise has been shown to attenuate glucose spikes, enhance insulin action, and reduce cardiovascular stress. Afternoon timing aligns with improved muscular performance and increased substrate utilization, and is often more feasible in daily routines compared to early mornings. These timing recommendations aim to optimize metabolic responses and improve long-term adherence.

### Future Directions

Although the present study did not directly assess cardiac outcomes, subclinical myocardial dysfunction is increasingly recognized in individuals with type 2 diabetes and obesity, often preceding overt cardiac disease. Speckle-tracking echocardiography (STE), particularly the assessment of left ventricular global longitudinal strain (LV-GLS), provides a sensitive tool for detecting early impairments in myocardial contractility, even in patients with preserved ejection fraction (170–172). Future longitudinal studies are warranted to determine whether ART and PMT confer cardioprotective effects by improving LV-GLS or other STE-derived parameters. Exploring this potential link may provide novel insights into integrated interventions for metabolic and cardiac health in diabetes.

## Conclusion

This study systematically evaluated the effects of various combined interventions on patients with T2DM and obesity through a network meta-analysis system. The results show that PMT is the most significant in improving blood lipid metabolism, especially in reducing TG, TC, LDL-C, and increasing HDL-C. In addition, ART has shown significant effects in improving multiple health indicators such as BMI, HbA1c%, and IL-6.

From the perspective of biomolecular mechanisms, PMT significantly improves insulin sensitivity and lipid metabolism by reducing stress hormone levels and improving endocrine and nervous system functions. ART activates the AMPK and mTOR signaling pathways, promotes fatty acid oxidation and muscle protein synthesis, reduces fat storage, and increases lean body mass, thereby effectively reducing BMI and HbA1c%. AT improves cardiopulmonary function and systemic metabolic status by increasing whole-body energy consumption and fat oxidation, helping to lower blood lipids and improve glucose metabolism. RT significantly reduces FBG and HbA1c% levels and improves glucose metabolism by increasing muscle mass and metabolic rate.

Based on the results of this study, it is recommended to use PMT and ART as the main interventions, combined with AT and RT to maximize the improvement of metabolic health in patients with T2DM and obesity. Both PMT and ART show comprehensive advantages in multiple outcome indicators and can significantly improve blood lipid metabolism, reduce inflammatory factor levels, and improve insulin sensitivity. Future research should further explore the individualized application of these interventions to optimize intervention strategies, improve clinical effects, and provide more scientific and effective management solutions for patients with T2DM and obesity.

## Data Availability

The raw data supporting the conclusions of this article will be made available by the authors, without undue reservation.
