# Supplementary figures and images for: Comparative effectiveness of various combined interventions for type 2 diabetes and obesity: a systematic review and network meta-analysis
Source: Front Endocrinol (Lausanne). 2025 Aug 6;16:1462104. doi: 10.3389/fendo.2025.1462104 (PMC12365605; doi:10.3389/fendo.2025.1462104)

**d.1.2**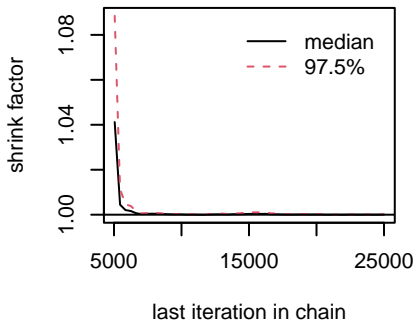**d.1.3**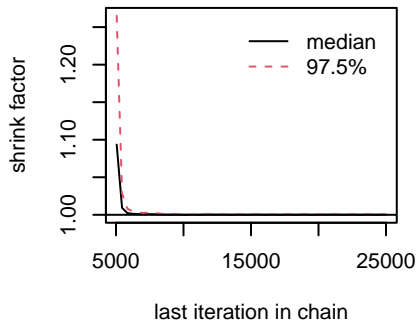**d.1.4**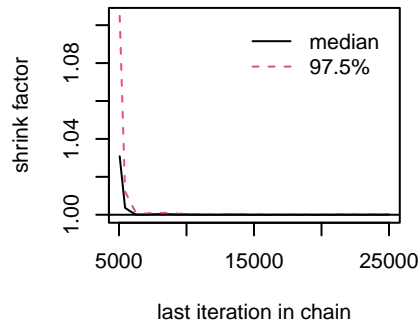**d.1.5**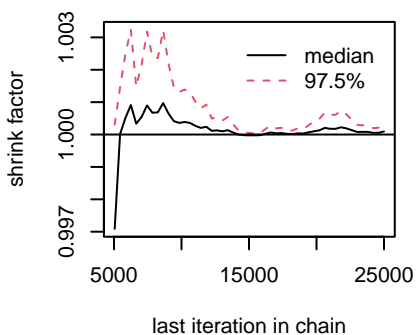**d.1.6**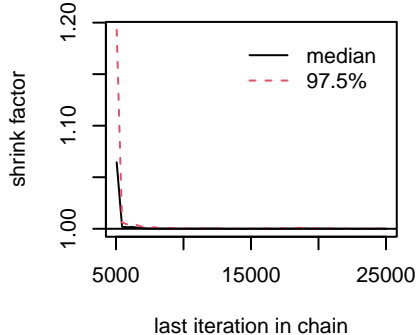**d.1.7**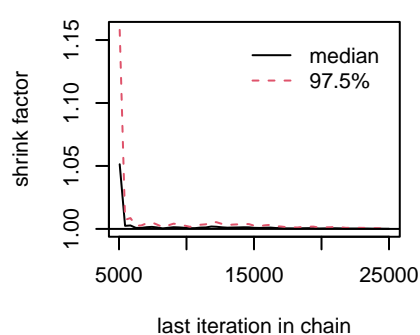**sd.d**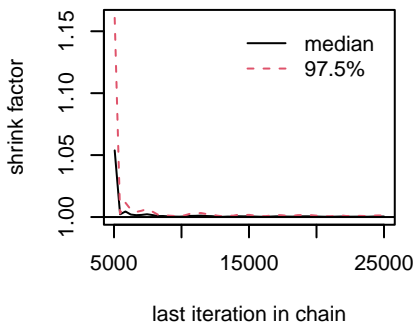

Supplement: Supplementary file 2 [file DataSheet1.zip › gelman/BMI_gelman plot.pdf]

**d.1.2**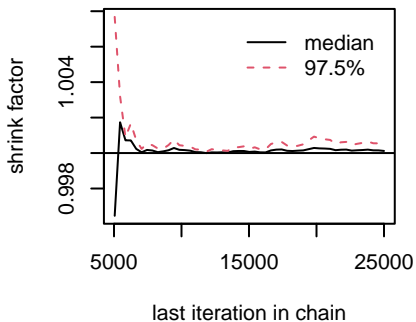**d.1.3**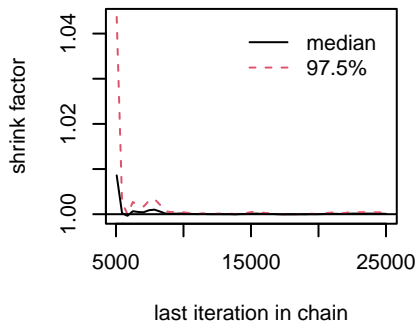**d.1.4**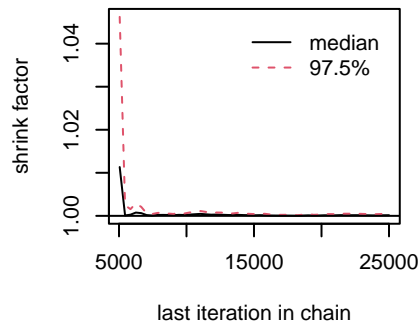**d.1.5**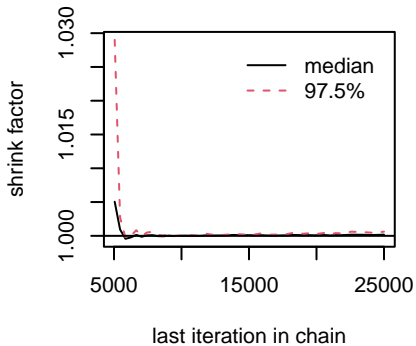**d.1.6**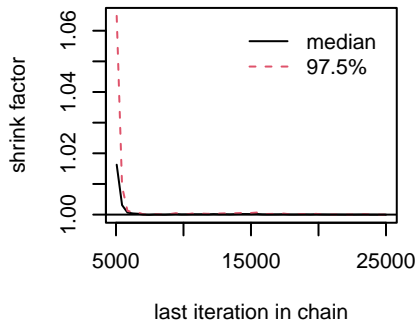**d.1.7**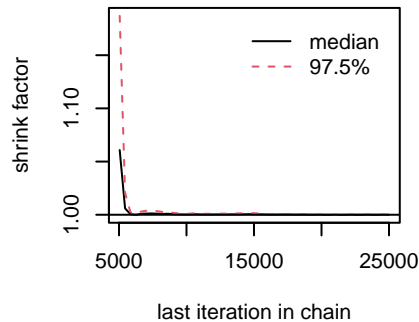**sd.d**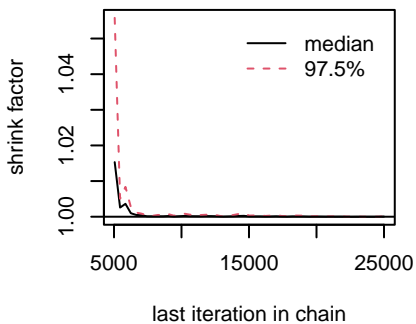

Supplement: Supplementary file 2 [file DataSheet1.zip › gelman/FBG_gelman plot.pdf]

**d.1.2**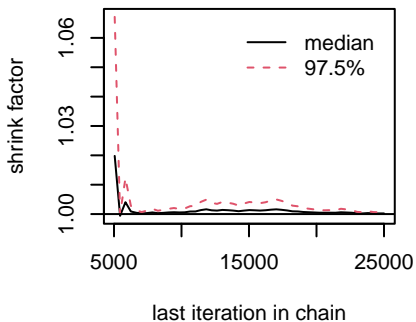**d.1.3**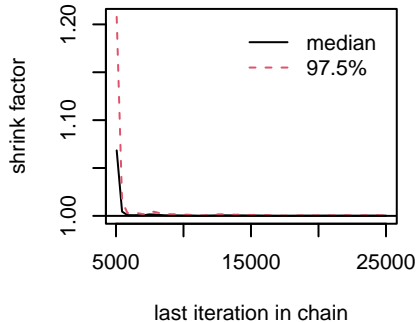**d.1.4**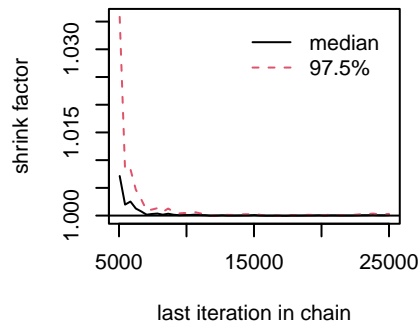**d.1.5**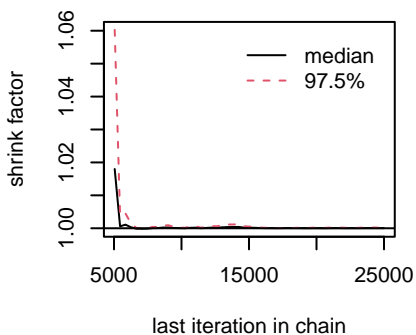**d.1.6**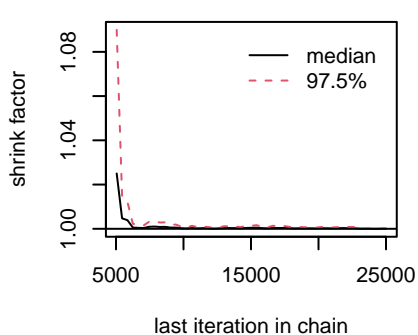**d.1.7**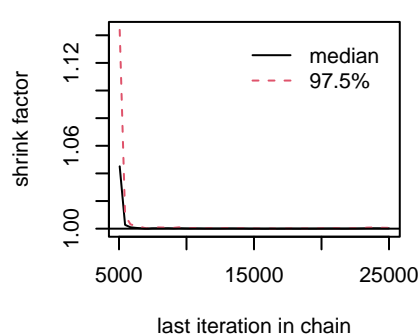**sd.d**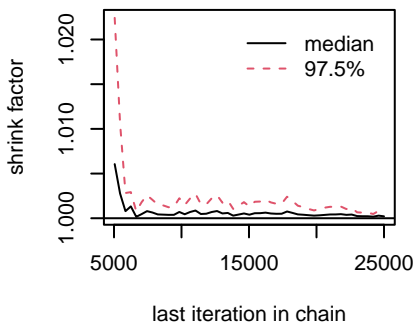

Supplement: Supplementary file 2 [file DataSheet1.zip › gelman/HbA1c%_gelman plot.pdf]

**d.1.2**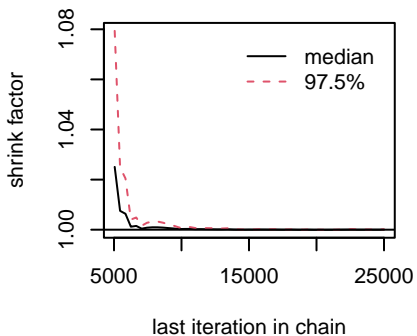**d.1.3**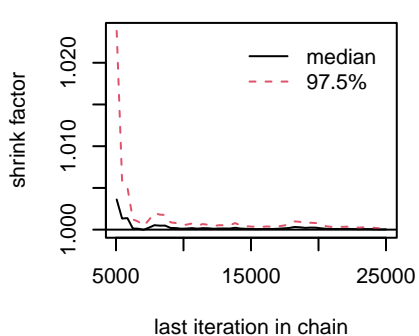**d.1.4**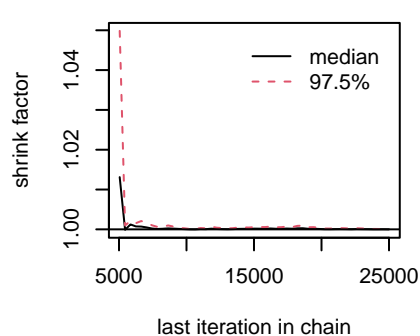**d.1.5**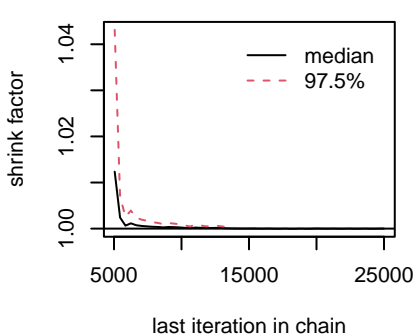**d.1.6**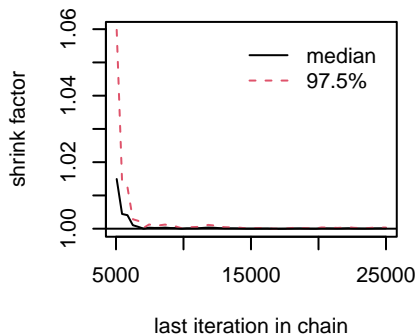**d.1.7**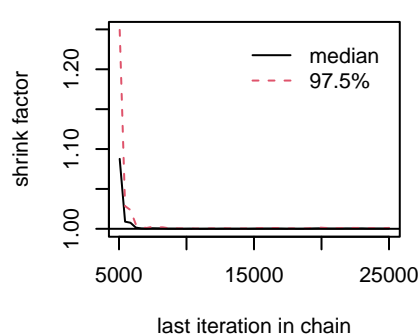**sd.d**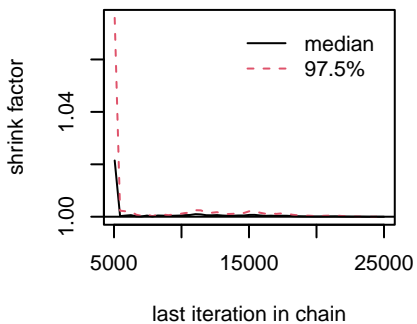

Supplement: Supplementary file 2 [file DataSheet1.zip › gelman/HDL-C_gelman plot.pdf]

**d.1.2**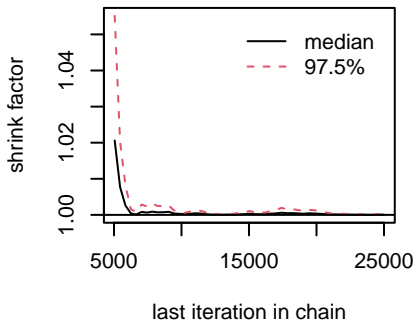**d.1.3**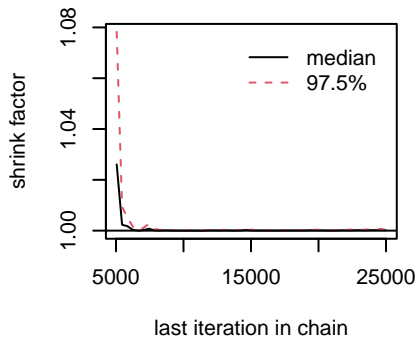**d.1.4**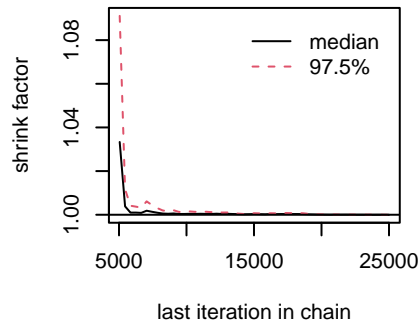**d.1.5**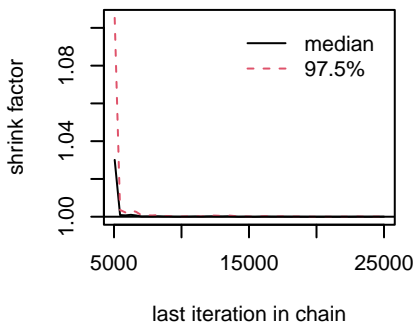**d.1.6**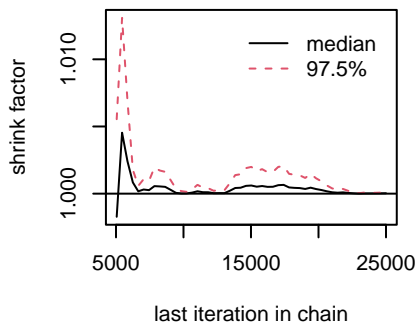**d.6.7**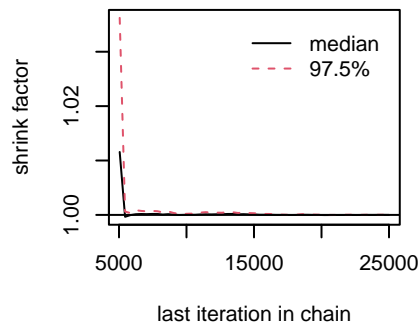**sd.d**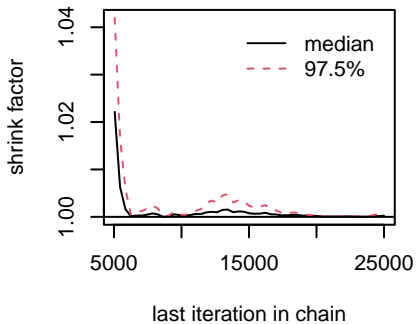

Supplement: Supplementary file 2 [file DataSheet1.zip › gelman/HOMA-IR_gelman plot.pdf]

**d.1.2**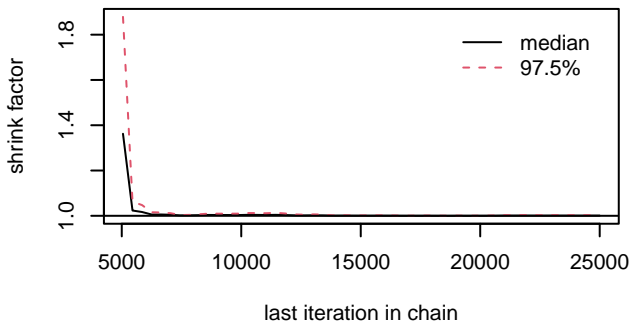**d.1.3**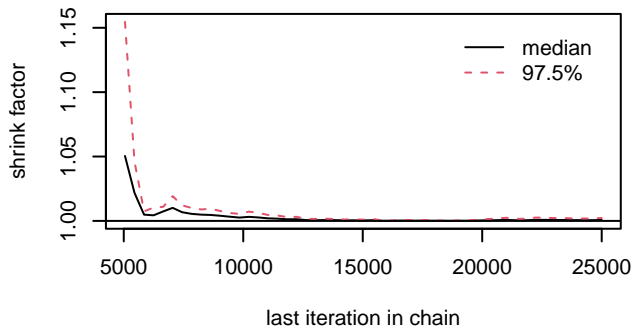**d.1.4**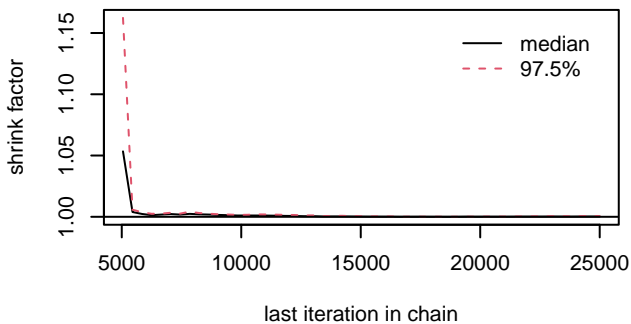**d.1.5**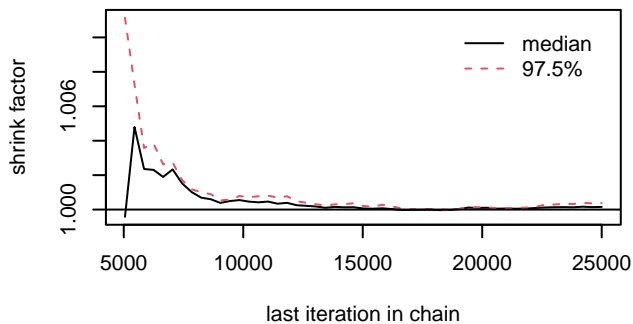**d.1.6**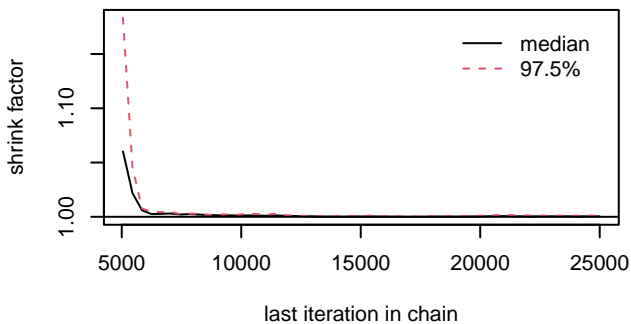**sd.d**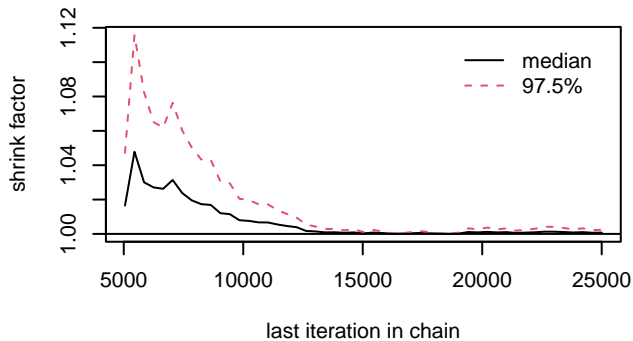

Supplement: Supplementary file 2 [file DataSheet1.zip › gelman/IL-6_gelman plot.pdf]

**d.1.2**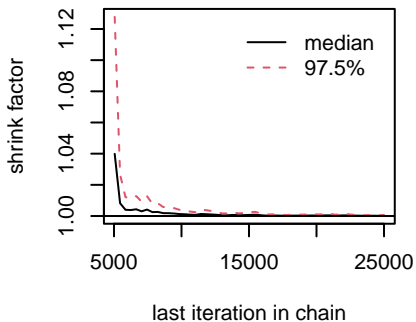**d.1.3**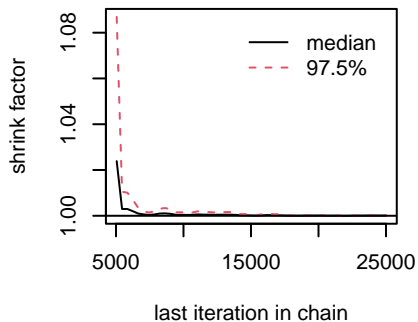**d.1.4**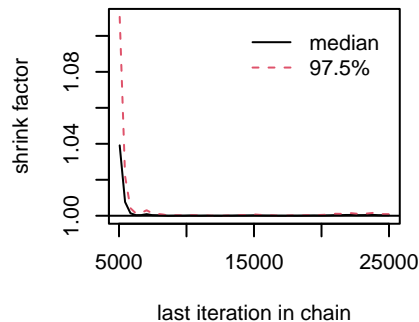**d.1.5**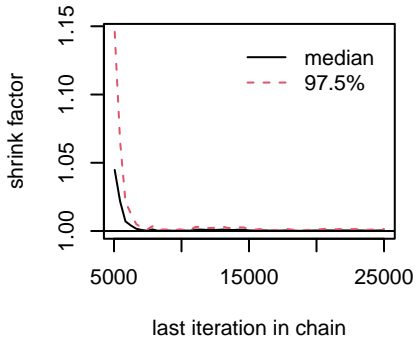**d.1.6**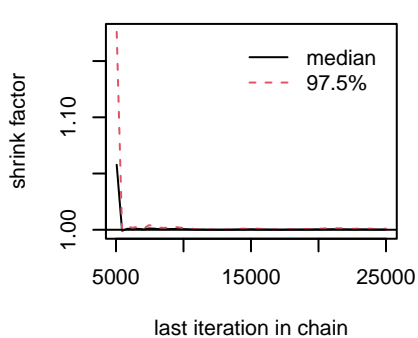**d.1.7**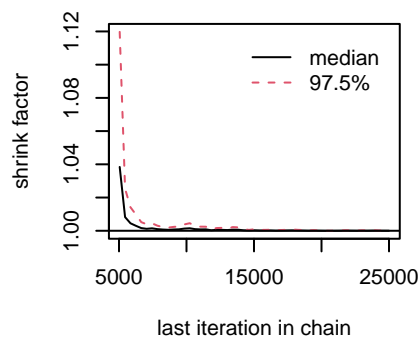**sd.d**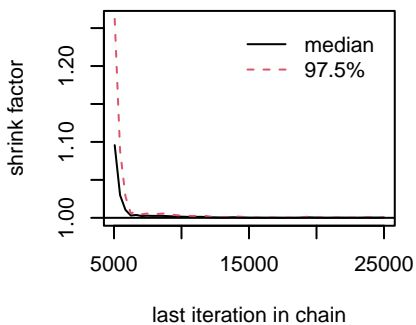

Supplement: Supplementary file 2 [file DataSheet1.zip › gelman/LDL-C_gelman plot.pdf]

**d.1.2**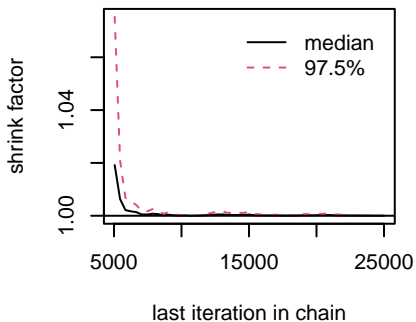**d.1.3**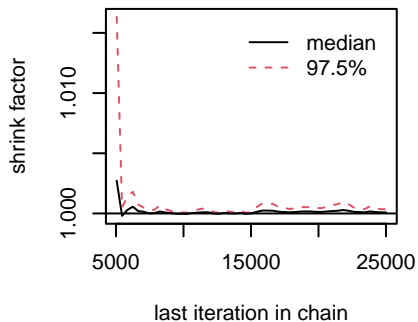**d.1.4**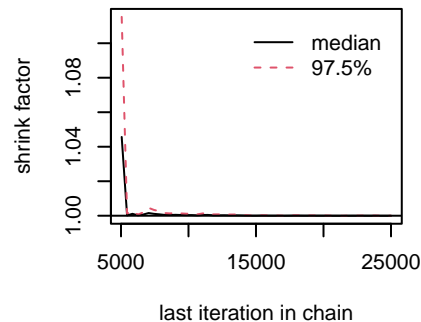**d.1.5**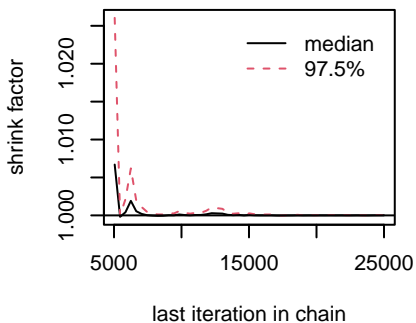**d.1.6**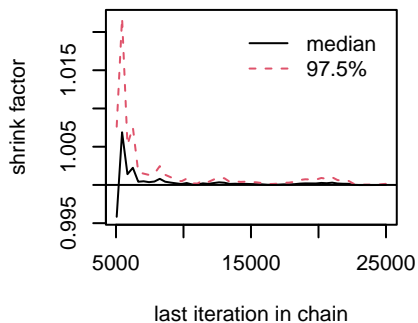**d.1.7**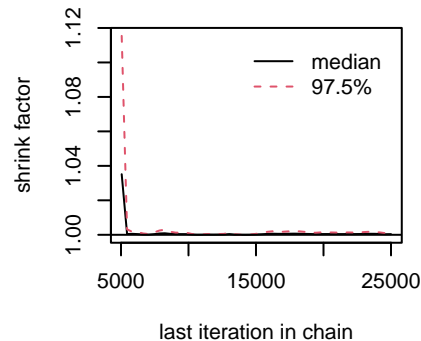**sd.d**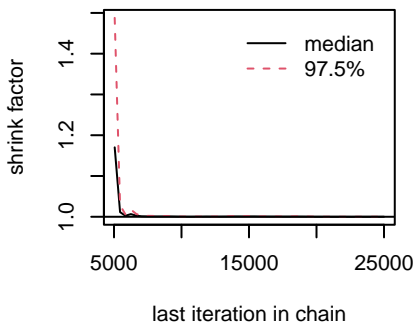

Supplement: Supplementary file 2 [file DataSheet1.zip › gelman/TC_gelman plot.pdf]

**d.1.2**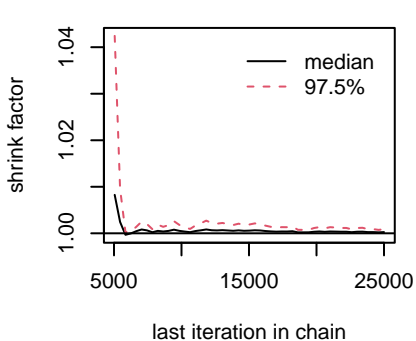**d.1.3**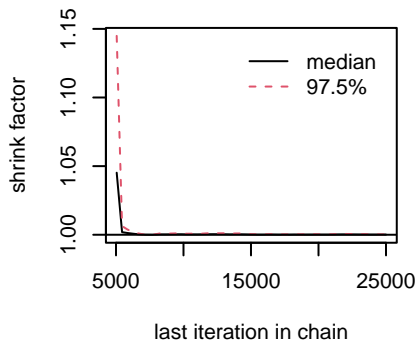**d.1.4**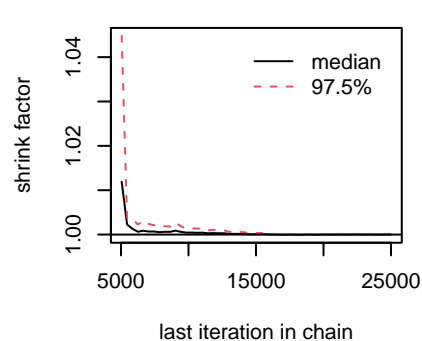**d.1.5**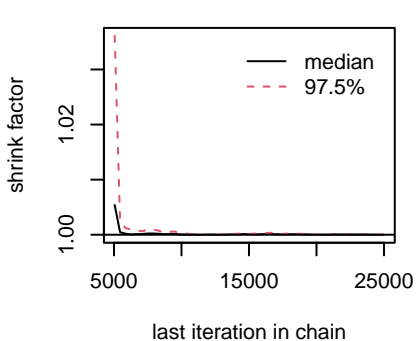**d.1.6**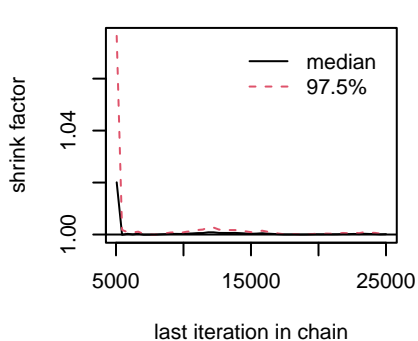**d.1.7**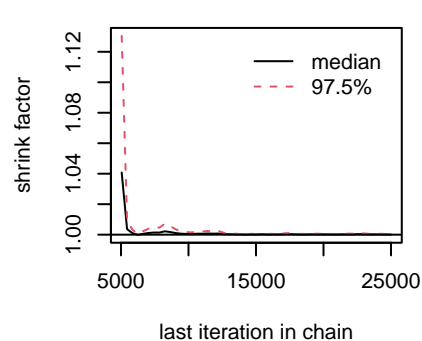**sd.d**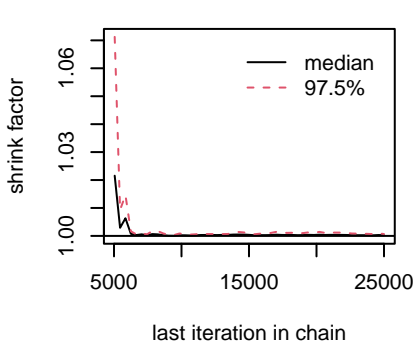

Supplement: Supplementary file 2 [file DataSheet1.zip › gelman/TG_gelman plot.pdf]

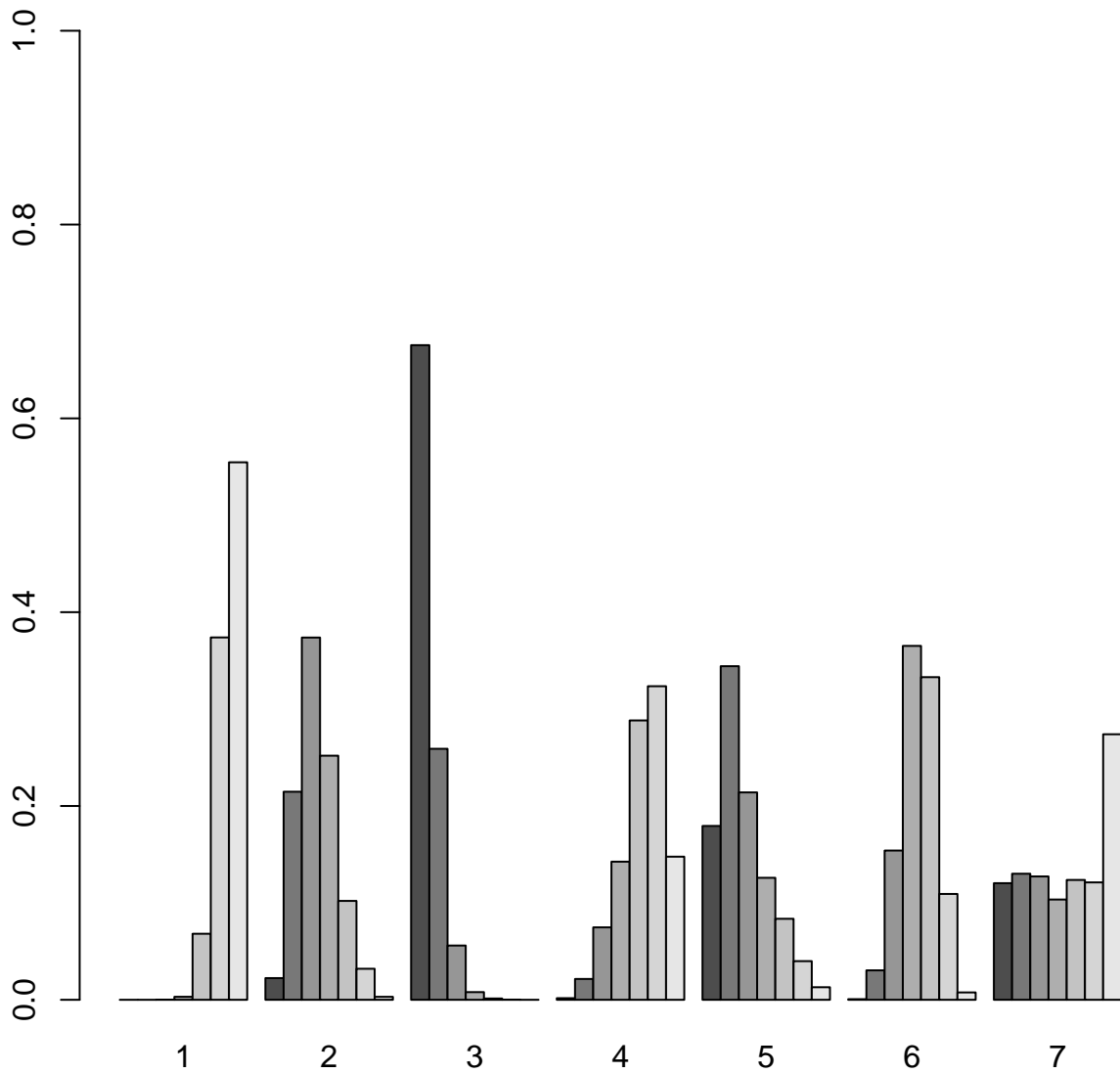

Supplement: Supplementary file 3 [file DataSheet2.zip › rank/BMI_ranko.pdf]

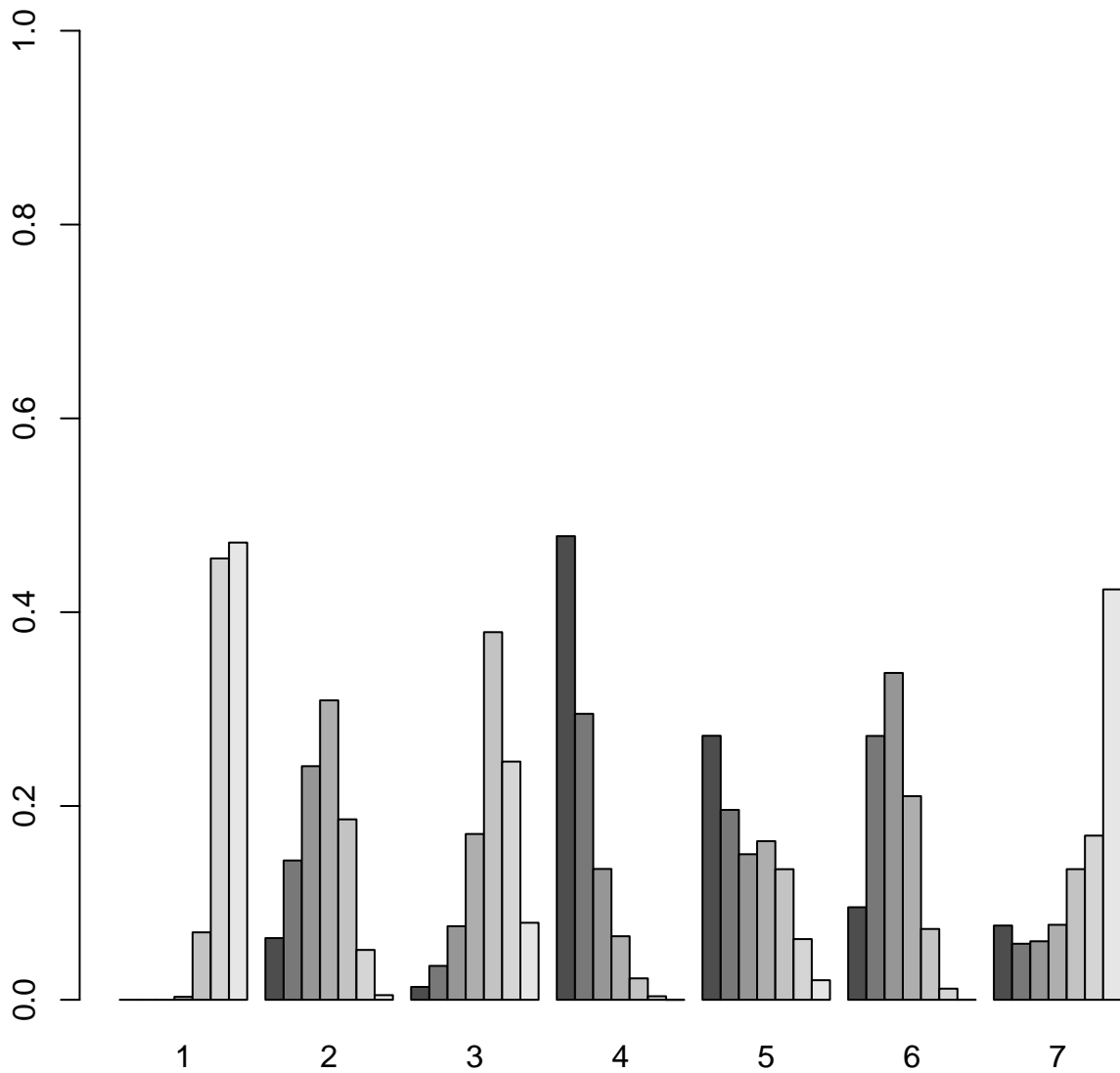

Supplement: Supplementary file 3 [file DataSheet2.zip › rank/FBG_ranko.pdf]

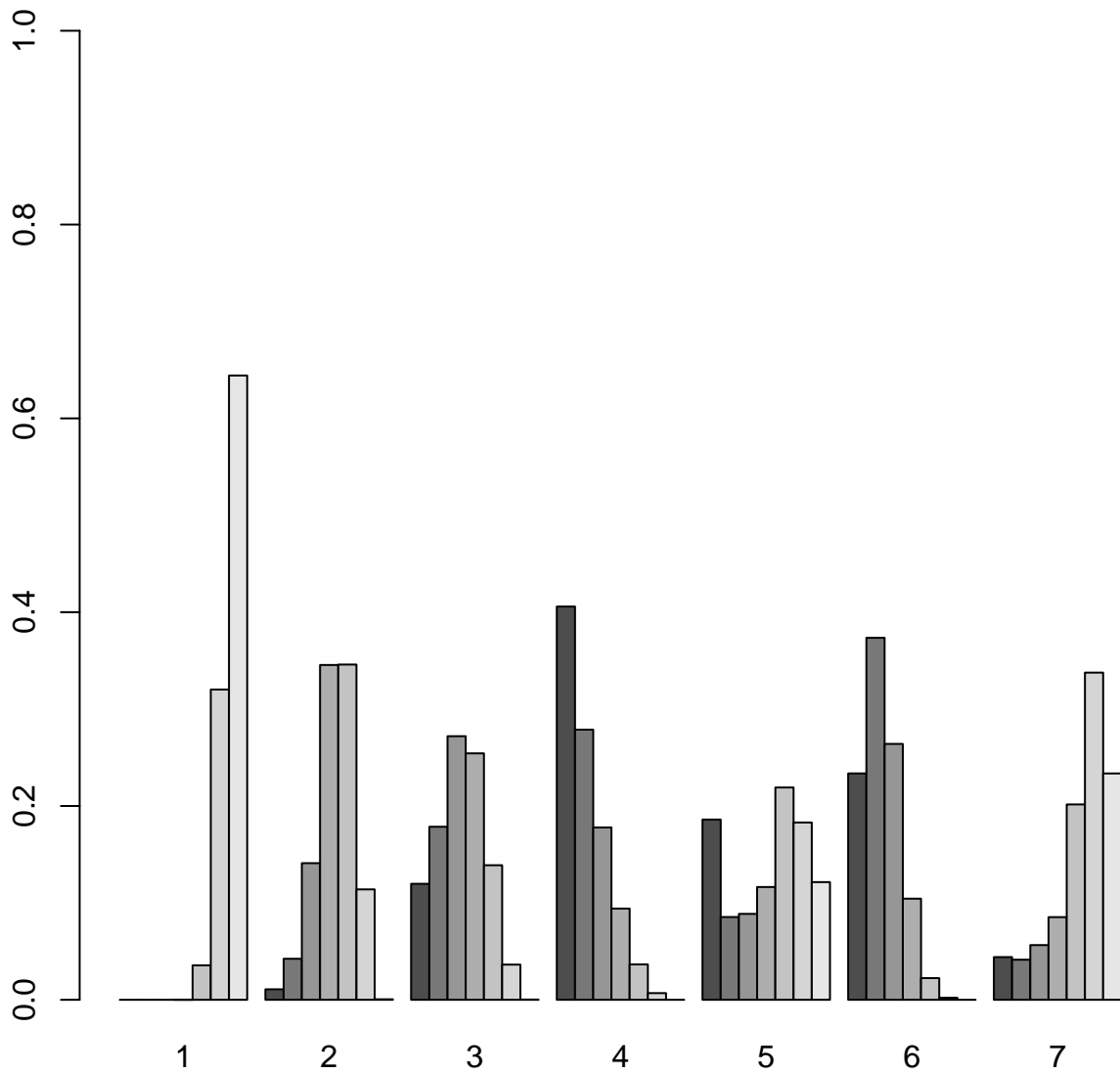

Supplement: Supplementary file 3 [file DataSheet2.zip › rank/HbA1c%_ranko.pdf]

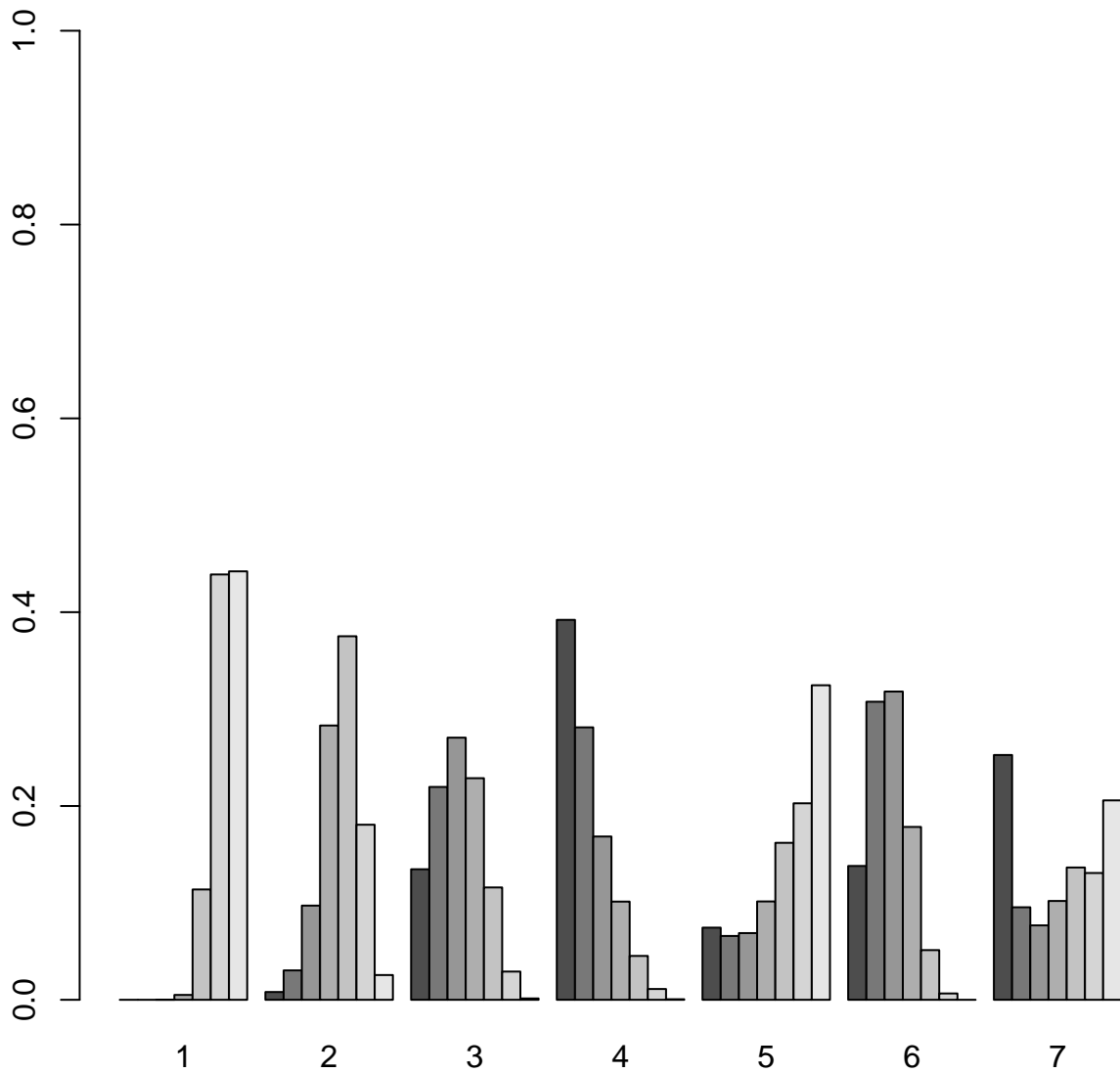

Supplement: Supplementary file 3 [file DataSheet2.zip › rank/HDL-C_ranko.pdf]

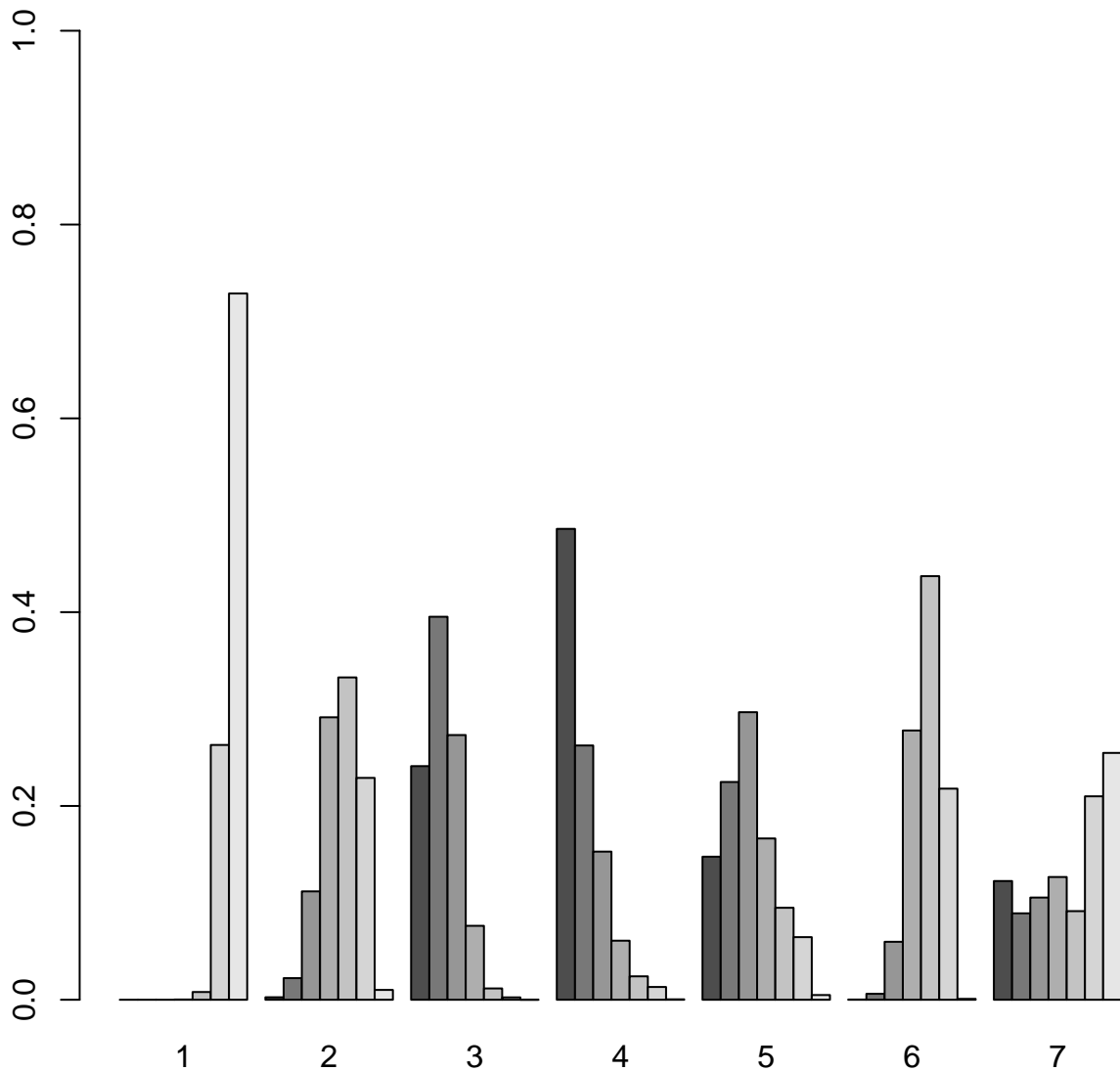

Supplement: Supplementary file 3 [file DataSheet2.zip › rank/HOMA-IR_ranko.pdf]

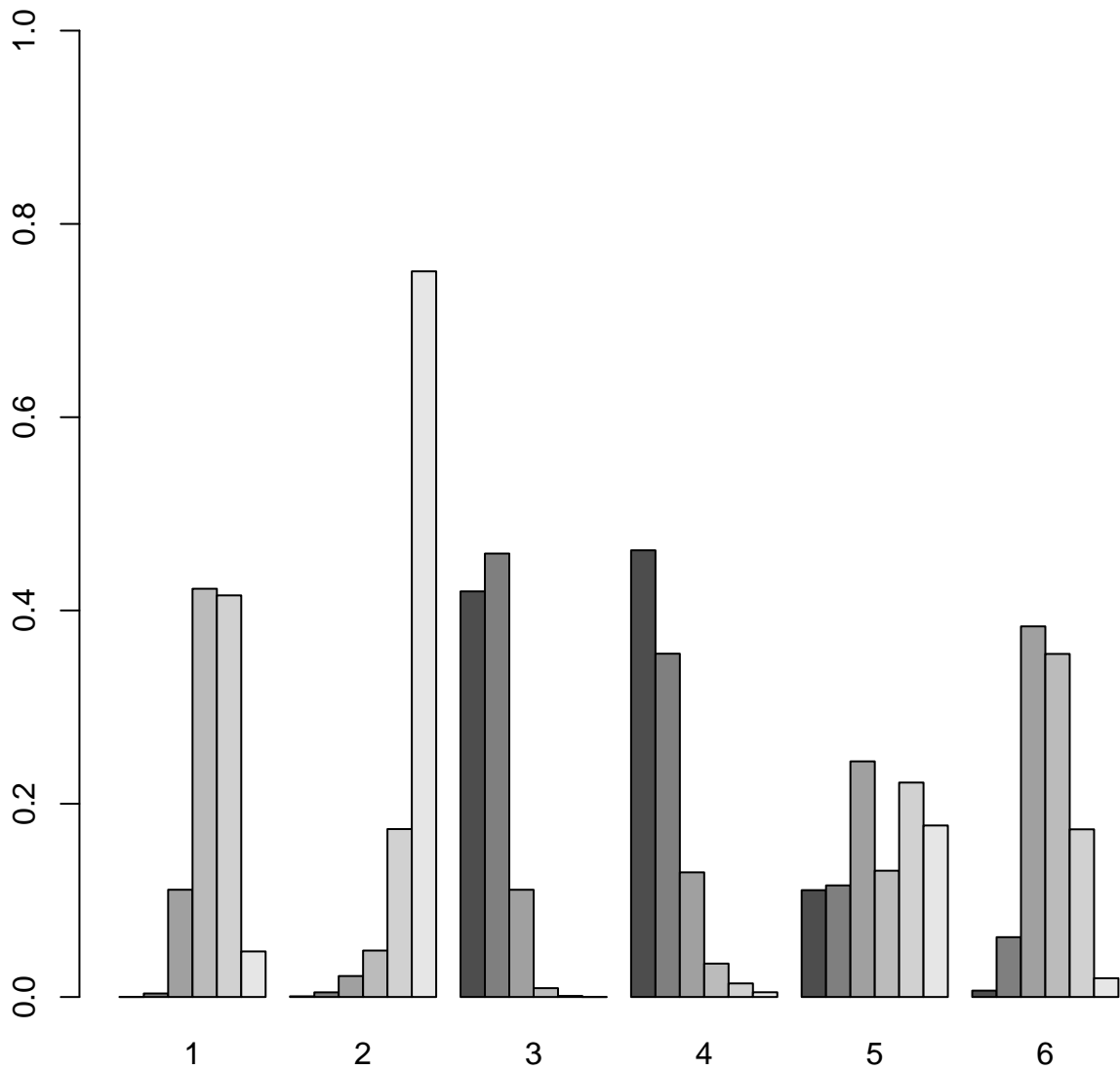

Supplement: Supplementary file 3 [file DataSheet2.zip › rank/IL-6_ranko.pdf]

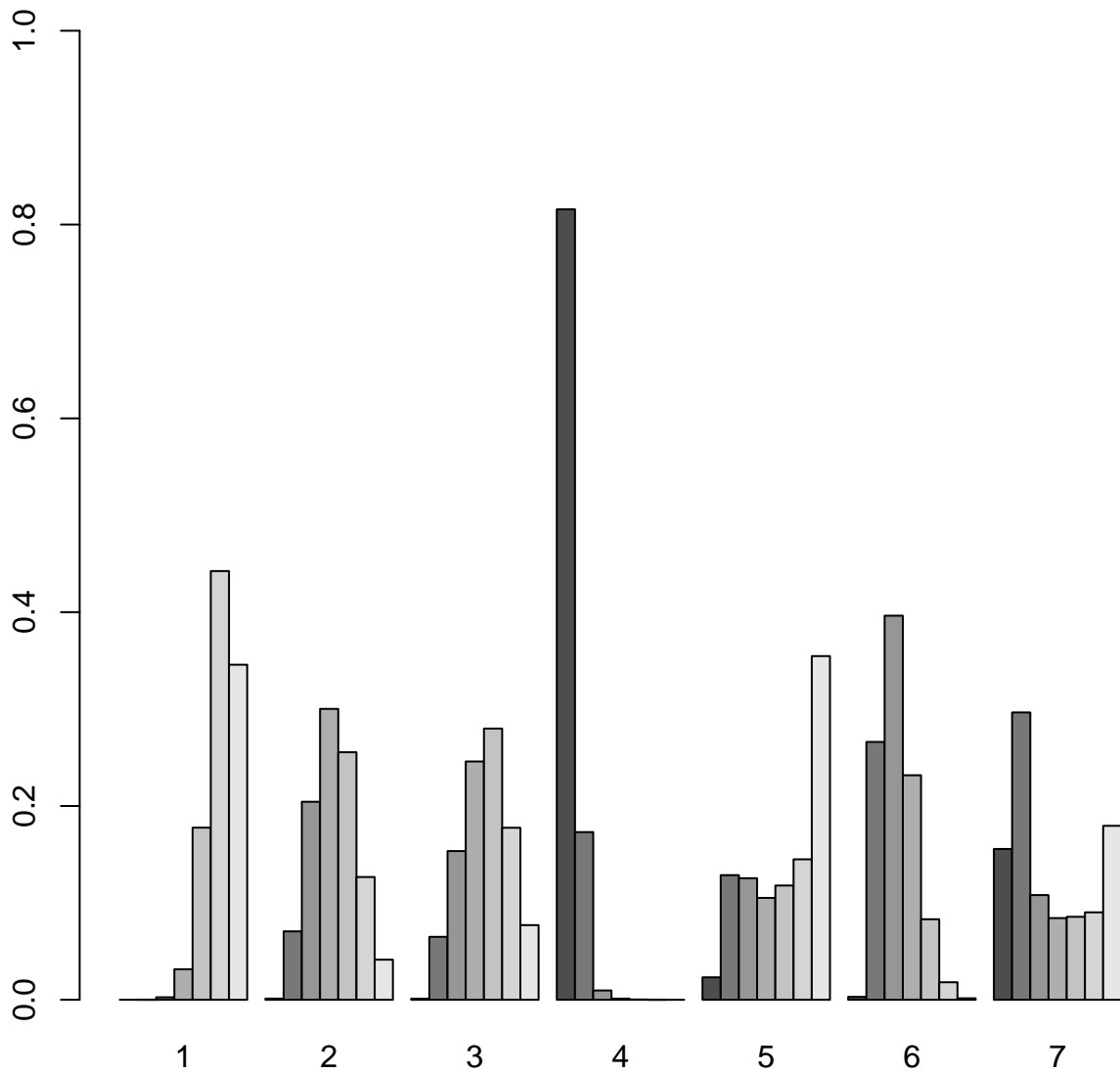

Supplement: Supplementary file 3 [file DataSheet2.zip › rank/LDL-C_ranko.pdf]

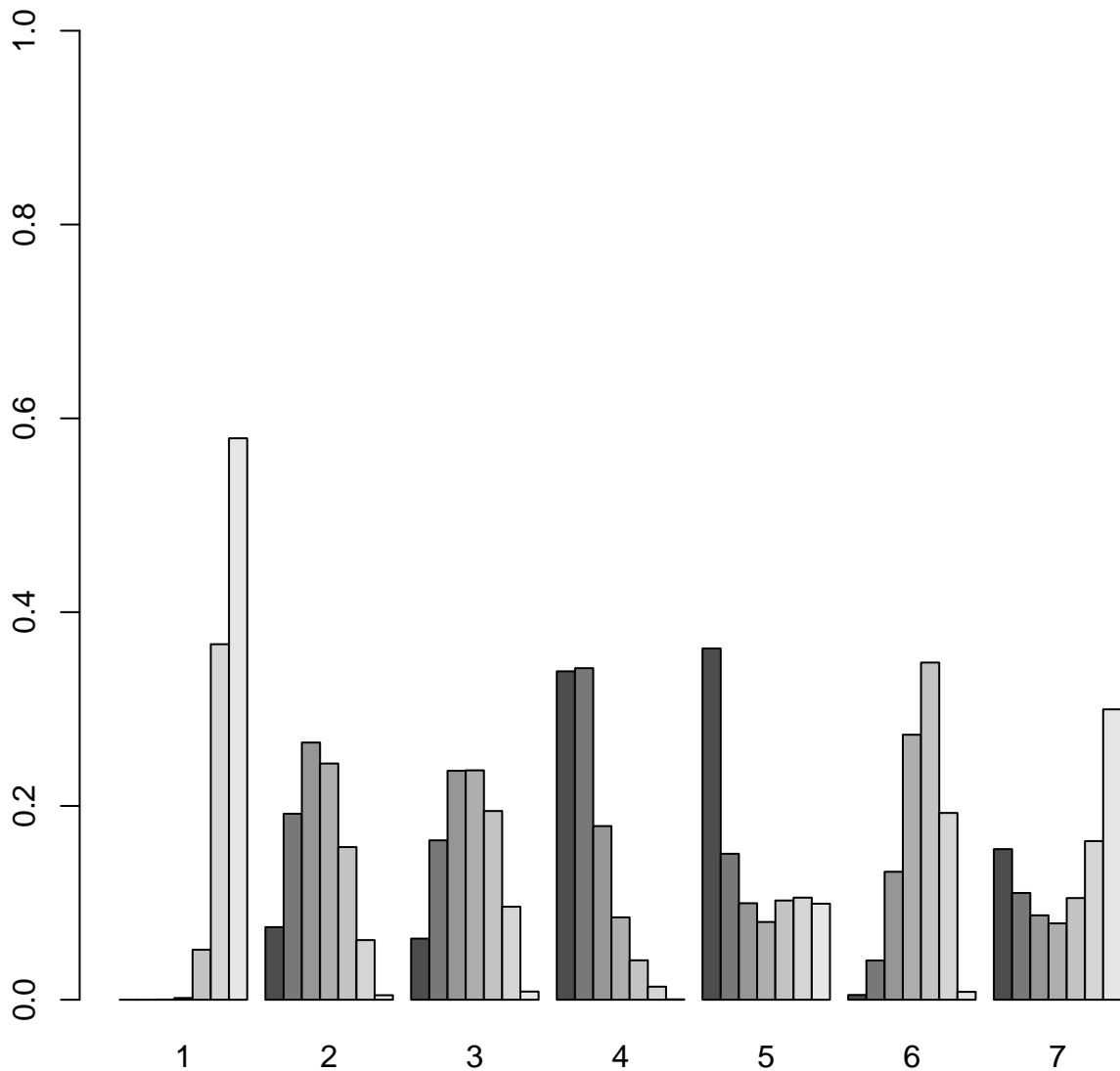

Supplement: Supplementary file 3 [file DataSheet2.zip › rank/TC_ranko.pdf]

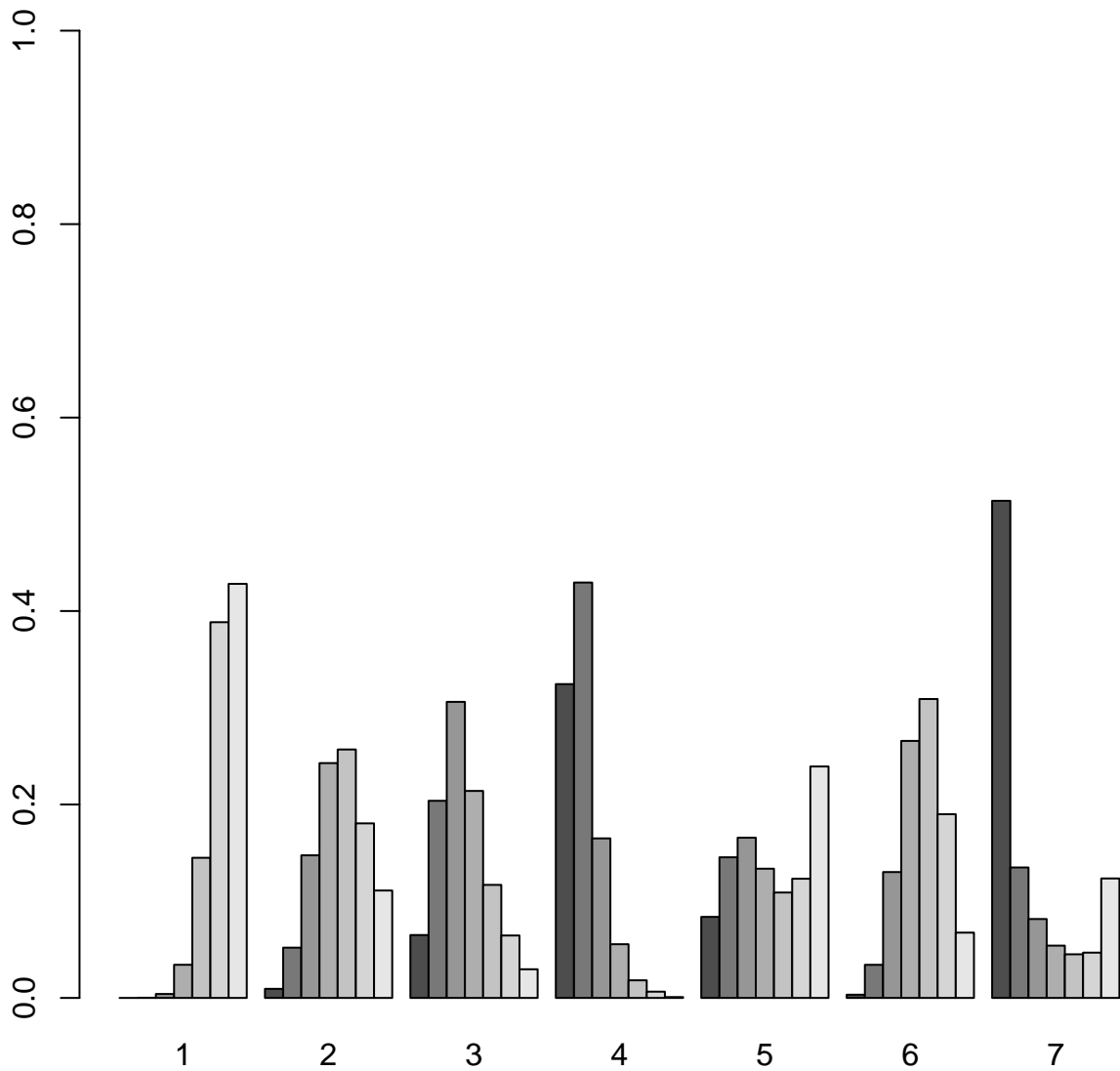

Supplement: Supplementary file 3 [file DataSheet2.zip › rank/TG_ranko.pdf]

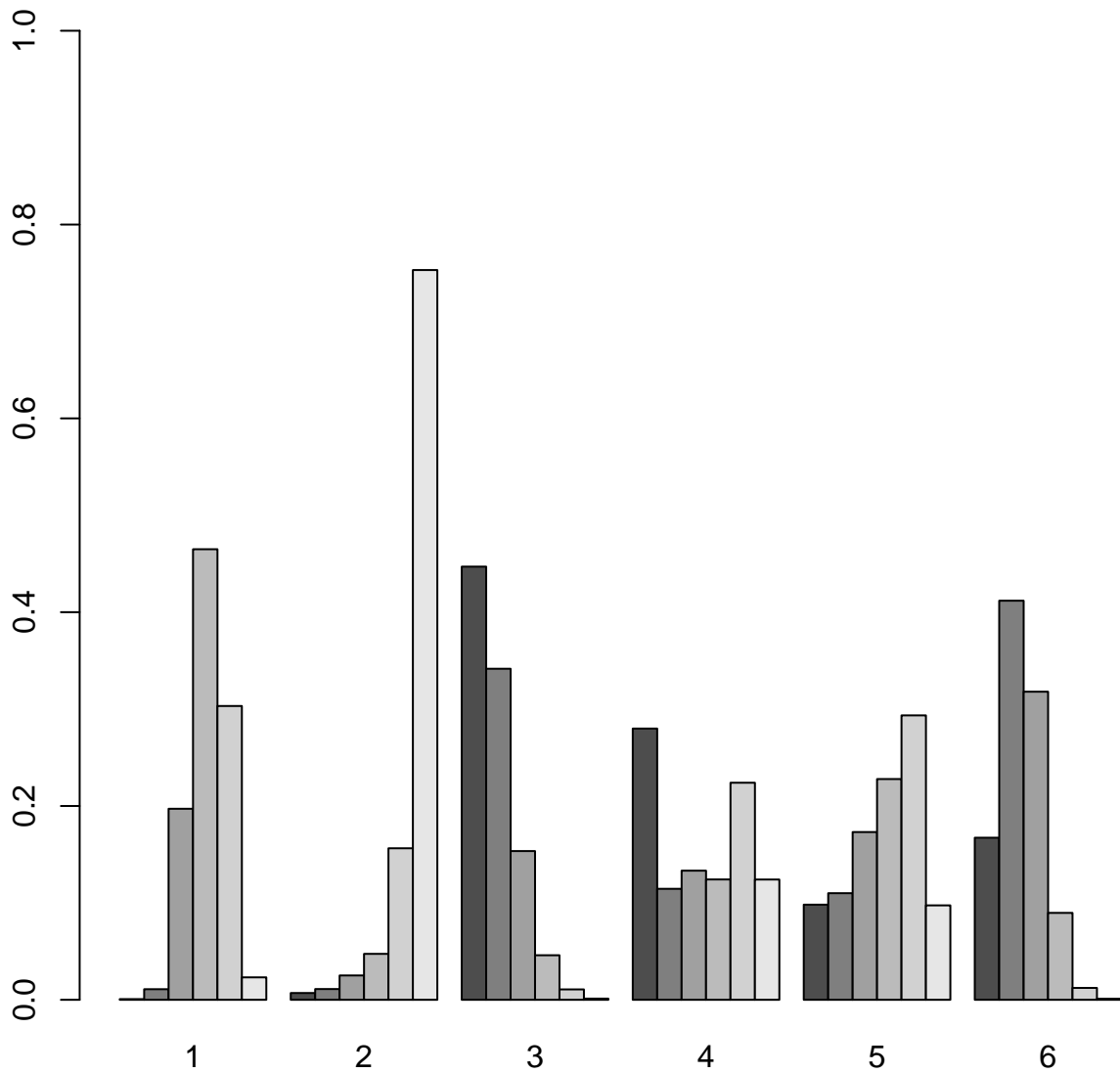

Supplement: Supplementary file 3 [file DataSheet2.zip › rank/TNF-a┴_ranko.pdf]
