# Supplementary material for: Comparative effectiveness of various combined interventions for type 2 diabetes and obesity: a systematic review and network meta-analysis
Source: Front Endocrinol (Lausanne). 2025 Aug 6;16:1462104. doi: 10.3389/fendo.2025.1462104 (PMC12365605; doi:10.3389/fendo.2025.1462104)
Supplement: Supplementary file 4 [file DataSheet3.docx]

| PubMed (through to [Date]) | Web of Science (through to [Date]) | CNKI (through to [Date]) | MEDLINE (through to [Date]) | EMBASE (through to [Date]) | Cochrane Central Register of Controlled Trials (through to [Date]) |
| --- | --- | --- | --- | --- | --- |
| 1. ("Diabetes Mellitus, Adult-Onset"[MeSH] OR "Adult-Onset Diabetes Mellitus") | 1. TS=("Diabetes Mellitus, Adult-Onset" OR "Adult-Onset Diabetes Mellitus") | 1. ("成人起病糖尿病" OR "成人起病的糖尿病") | 1. ("Diabetes Mellitus, Adult-Onset" OR "Adult-Onset Diabetes Mellitus").mp. | 1. ("Diabetes Mellitus, Adult-Onset" OR "Adult-Onset Diabetes Mellitus").mp. | 1. ("Diabetes Mellitus, Adult-Onset" OR "Adult-Onset Diabetes Mellitus").mp. |
| 2. ("Diabetes Mellitus, Adult Onset" OR "Diabetes Mellitus, Ketosis-Resistant") | 2. TS=("Diabetes Mellitus, Adult Onset" OR "Diabetes Mellitus, Ketosis-Resistant") | 2. ("成人起病糖尿病" OR "酮症抵抗糖尿病") | 2. ("Diabetes Mellitus, Adult Onset" OR "Diabetes Mellitus, Ketosis-Resistant").mp. | 2. ("Diabetes Mellitus, Adult Onset" OR "Diabetes Mellitus, Ketosis-Resistant").mp. | 2. ("Diabetes Mellitus, Adult Onset" OR "Diabetes Mellitus, Ketosis-Resistant").mp. |
| 3. ("Diabetes Mellitus, Non-Insulin-Dependent" OR "Non-Insulin-Dependent Diabetes Mellitus") | 3. TS=("Diabetes Mellitus, Non-Insulin-Dependent" OR "Non-Insulin-Dependent Diabetes Mellitus") | 3. ("非胰岛素依赖型糖尿病" OR "非胰岛素依赖的糖尿病") | 3. ("Diabetes Mellitus, Non-Insulin-Dependent" OR "Non-Insulin-Dependent Diabetes Mellitus").mp. | 3. ("Diabetes Mellitus, Non-Insulin-Dependent" OR "Non-Insulin-Dependent Diabetes Mellitus").mp. | 3. ("Diabetes Mellitus, Non-Insulin-Dependent" OR "Non-Insulin-Dependent Diabetes Mellitus").mp. |
| 4. ("Diabetes Mellitus, Stable" OR "Stable Diabetes Mellitus") | 4. TS=("Diabetes Mellitus, Stable" OR "Stable Diabetes Mellitus") | 4. ("稳定型糖尿病" OR "稳定的糖尿病") | 4. ("Diabetes Mellitus, Stable" OR "Stable Diabetes Mellitus").mp. | 4. ("Diabetes Mellitus, Stable" OR "Stable Diabetes Mellitus").mp. | 4. ("Diabetes Mellitus, Stable" OR "Stable Diabetes Mellitus").mp. |
| 5. ("Diabetes Mellitus, Type II" OR "NIDDM") | 5. TS=("Diabetes Mellitus, Type II" OR "NIDDM") | 5. ("2型糖尿病" OR "NIDDM") | 5. ("Diabetes Mellitus, Type II" OR "NIDDM").mp. | 5. ("Diabetes Mellitus, Type II" OR "NIDDM").mp. | 5. ("Diabetes Mellitus, Type II" OR "NIDDM").mp. |
| 6. ("Diabetes Mellitus, Noninsulin Dependent" OR "Diabetes Mellitus, Maturity-Onset") | 6. TS=("Diabetes Mellitus, Noninsulin Dependent" OR "Diabetes Mellitus, Maturity-Onset") | 6. ("非胰岛素依赖型糖尿病" OR "成年发病糖尿病") | 6. ("Diabetes Mellitus, Noninsulin Dependent" OR "Diabetes Mellitus, Maturity-Onset").mp. | 6. ("Diabetes Mellitus, Noninsulin Dependent" OR "Diabetes Mellitus, Maturity-Onset").mp. | 6. ("Diabetes Mellitus, Noninsulin Dependent" OR "Diabetes Mellitus, Maturity-Onset").mp. |
| 7. ("Diabetes Mellitus, Maturity Onset" OR "Maturity-Onset Diabetes Mellitus") | 7. TS=("Diabetes Mellitus, Maturity Onset" OR "Maturity-Onset Diabetes Mellitus") | 7. ("成年发病糖尿病" OR "成年起病糖尿病") | 7. ("Diabetes Mellitus, Maturity Onset" OR "Maturity-Onset Diabetes Mellitus").mp. | 7. ("Diabetes Mellitus, Maturity Onset" OR "Maturity-Onset Diabetes Mellitus").mp. | 7. ("Diabetes Mellitus, Maturity Onset" OR "Maturity-Onset Diabetes Mellitus").mp. |
| 8. ("MODY" OR "Diabetes Mellitus, Slow-Onset") | 8. TS=("MODY" OR "Diabetes Mellitus, Slow-Onset") | 8. ("MODY" OR "缓慢起病糖尿病") | 8. ("MODY" OR "Diabetes Mellitus, Slow-Onset").mp. | 8. ("MODY" OR "Diabetes Mellitus, Slow-Onset").mp. | 8. ("MODY" OR "Diabetes Mellitus, Slow-Onset").mp. |
| 9. ("Diabetes Mellitus, Slow Onset" OR "Slow-Onset Diabetes Mellitus") | 9. TS=("Diabetes Mellitus, Slow Onset" OR "Slow-Onset Diabetes Mellitus") | 9. ("缓慢起病糖尿病" OR "迟发型糖尿病") | 9. ("Diabetes Mellitus, Slow Onset" OR "Slow-Onset Diabetes Mellitus").mp. | 9. ("Diabetes Mellitus, Slow Onset" OR "Slow-Onset Diabetes Mellitus").mp. | 9. ("Diabetes Mellitus, Slow Onset" OR "Slow-Onset Diabetes Mellitus").mp. |
| 10. ("Type 2 Diabetes Mellitus" OR "Noninsulin-Dependent Diabetes Mellitus") | 10. TS=("Type 2 Diabetes Mellitus" OR "Noninsulin-Dependent Diabetes Mellitus") | 10. ("2型糖尿病" OR "非胰岛素依赖型糖尿病") | 10. ("Type 2 Diabetes Mellitus" OR "Noninsulin-Dependent Diabetes Mellitus").mp. | 10. ("Type 2 Diabetes Mellitus" OR "Noninsulin-Dependent Diabetes Mellitus").mp. | 10. ("Type 2 Diabetes Mellitus" OR "Noninsulin-Dependent Diabetes Mellitus").mp. |
| 11. ("Noninsulin Dependent Diabetes Mellitus" OR "Maturity-Onset Diabetes") | 11. TS=("Noninsulin Dependent Diabetes Mellitus" OR "Maturity-Onset Diabetes") | 11. ("非胰岛素依赖型糖尿病" OR "成年发病糖尿病") | 11. ("Noninsulin Dependent Diabetes Mellitus" OR "Maturity-Onset Diabetes").mp. | 11. ("Noninsulin Dependent Diabetes Mellitus" OR "Maturity-Onset Diabetes").mp. | 11. ("Noninsulin Dependent Diabetes Mellitus" OR "Maturity-Onset Diabetes").mp. |
| 12. ("Diabetes, Maturity-Onset" OR "Maturity Onset Diabetes") | 12. TS=("Diabetes, Maturity-Onset" OR "Maturity Onset Diabetes") | 12. ("成年发病糖尿病" OR "成年起病糖尿病") | 12. ("Diabetes, Maturity-Onset" OR "Maturity Onset Diabetes").mp. | 12. ("Diabetes, Maturity-Onset" OR "Maturity Onset Diabetes").mp. | 12. ("Diabetes, Maturity-Onset" OR "Maturity Onset Diabetes").mp. |
| 13. ("Type 2 Diabetes" OR "Diabetes, Type 2") | 13. TS=("Type 2 Diabetes" OR "Diabetes, Type 2") | 13. ("2型糖尿病" OR "2型糖尿病") | 13. ("Type 2 Diabetes" OR "Diabetes, Type 2").mp. | 13. ("Type 2 Diabetes" OR "Diabetes, Type 2").mp. | 13. ("Type 2 Diabetes" OR "Diabetes, Type 2").mp. |
| 14. ("Diabetes Mellitus, Noninsulin-Dependent" OR "Obesity") | 14. TS=("Diabetes Mellitus, Noninsulin-Dependent" OR "Obesity") | 14. ("非胰岛素依赖型糖尿病" OR "肥胖") | 14. ("Diabetes Mellitus, Noninsulin-Dependent" OR "Obesity").mp. | 14. ("Diabetes Mellitus, Noninsulin-Dependent" OR "Obesity").mp. | 14. ("Diabetes Mellitus, Noninsulin-Dependent" OR "Obesity").mp. |
| 15. ("Overweight") | 15. TS=("Overweight") | 15. ("超重") | 15. ("Overweight").mp. | 15. ("Overweight").mp. | 15. ("Overweight").mp. |
| 16. ("Exercise, Aerobic" OR "Aerobic Exercise") | 16. TS=("Exercise, Aerobic" OR "Aerobic Exercise") | 16. ("有氧运动" OR "有氧锻炼") | 16. ("Exercise, Aerobic" OR "Aerobic Exercise").mp. | 16. ("Exercise, Aerobic" OR "Aerobic Exercise").mp. | 16. ("Exercise, Aerobic" OR "Aerobic Exercise").mp. |
| 17. ("Aerobic Exercises" OR "Exercises, Aerobic") | 17. TS=("Aerobic Exercises" OR "Exercises, Aerobic") | 17. ("有氧运动" OR "有氧锻炼") | 17. ("Aerobic Exercises" OR "Exercises, Aerobic").mp. | 17. ("Aerobic Exercises" OR "Exercises, Aerobic").mp. | 17. ("Aerobic Exercises" OR "Exercises, Aerobic").mp. |
| 18. ("Endurance Training" OR "Walking") | 18. TS=("Endurance Training" OR "Walking") | 18. ("耐力训练" OR "步行") | 18. ("Endurance Training" OR "Walking").mp. | 18. ("Endurance Training" OR "Walking").mp. | 18. ("Endurance Training" OR "Walking").mp. |
| 19. ("Training, Resistance" OR "Strength Training") | 19. TS=("Training, Resistance" OR "Strength Training") | 19. ("阻力训练" OR "力量训练") | 19. ("Training, Resistance" OR "Strength Training").mp. | 19. ("Training, Resistance" OR "Strength Training").mp. | 19. ("Training, Resistance" OR "Strength Training").mp. |
| 20. ("Training, Strength" OR "Weight-Lifting Strengthening Program") | 20. TS=("Training, Strength" OR "Weight-Lifting Strengthening Program") | 20. ("力量训练" OR "举重强化计划") | 20. ("Training, Strength" OR "Weight-Lifting Strengthening Program").mp. | 20. ("Training, Strength" OR "Weight-Lifting Strengthening Program").mp. | 20. ("Training, Strength" OR "Weight-Lifting Strengthening Program").mp. |
| 21. ("Strengthening Programs, Weight-Lifting" OR "Resistance Training") | 21. TS=("Strengthening Programs, Weight-Lifting" OR "Resistance Training") | 21. ("举重强化计划" OR "阻力训练") | 21. ("Strengthening Programs, Weight-Lifting" OR "Resistance Training").mp. | 21. ("Strengthening Programs, Weight-Lifting" OR "Resistance Training").mp. | 21. ("Strengthening Programs, Weight-Lifting" OR "Resistance Training").mp. |
| 22. ("Mind-Body Therapies" OR "Yoga") | 22. TS=("Mind-Body Therapies" OR "Yoga") | 22. ("身心疗法" OR "瑜伽") | 22. ("Mind-Body Therapies" OR "Yoga").mp. | 22. ("Mind-Body Therapies" OR "Yoga").mp. | 22. ("Mind-Body Therapies" OR "Yoga").mp. |
| 23. ("Tai Ji" OR "Laughter Therapy") | 23. TS=("Tai Ji" OR "Laughter Therapy") | 23. ("太极" OR "笑疗法") | 23. ("Tai Ji" OR "Laughter Therapy").mp. | 23. ("Tai Ji" OR "Laughter Therapy").mp. | 23. ("Tai Ji" OR "Laughter Therapy").mp. |
| 24. ("Qigong" OR "Acupuncture Treatment") | 24. TS=("Qigong" OR "Acupuncture Treatment") | 24. ("气功" OR "针灸治疗") | 24. ("Qigong" OR "Acupuncture Treatment").mp. | 24. ("Qigong" OR "Acupuncture Treatment").mp. | 24. ("Qigong" OR "Acupuncture Treatment").mp. |
| 25. ("Acupuncture Treatments" OR "Treatment, Acupuncture") | 25. TS=("Acupuncture Treatments" OR "Treatment, Acupuncture") | 25. ("针灸治疗" OR "针灸疗法") | 25. ("Acupuncture Treatments" OR "Treatment, Acupuncture").mp. | 25. ("Acupuncture Treatments" OR "Treatment, Acupuncture").mp. | 25. ("Acupuncture Treatments" OR "Treatment, Acupuncture").mp. |
| 26. ("Therapy, Acupuncture" OR "Pharmacoacupuncture Treatment") | 26. TS=("Therapy, Acupuncture" OR "Pharmacoacupuncture Treatment") | 26. ("针灸疗法" OR "药针治疗") | 26. ("Therapy, Acupuncture" OR "Pharmacoacupuncture Treatment").mp. | 26. ("Therapy, Acupuncture" OR "Pharmacoacupuncture Treatment").mp. | 26. ("Therapy, Acupuncture" OR "Pharmacoacupuncture Treatment").mp. |
| 27. ("Treatment, Pharmacoacupuncture" OR "Pharmacoacupuncture Therapy") | 27. TS=("Treatment, Pharmacoacupuncture" OR "Pharmacoacupuncture Therapy") | 27. ("药针治疗" OR "药针疗法") | 27. ("Treatment, Pharmacoacupuncture" OR "Pharmacoacupuncture Therapy").mp. | 27. ("Treatment, Pharmacoacupuncture" OR "Pharmacoacupuncture Therapy").mp. | 27. ("Treatment, Pharmacoacupuncture" OR "Pharmacoacupuncture Therapy").mp. |
| 28. ("Therapy, Pharmacoacupuncture" OR "Acupotomy") | 28. TS=("Therapy, Pharmacoacupuncture" OR "Acupotomy") | 28. ("药针疗法" OR "针刀") | 28. ("Therapy, Pharmacoacupuncture" OR "Acupotomy").mp. | 28. ("Therapy, Pharmacoacupuncture" OR "Acupotomy").mp. | 28. ("Therapy, Pharmacoacupuncture" OR "Acupotomy").mp. |
| 29. ("Acupotomies" OR "whole body vibration training") | 29. TS=("Acupotomies" OR "whole body vibration training") | 29. ("针刀" OR "全身振动训练") | 29. ("Acupotomies" OR "whole body vibration training").mp. | 29. ("Acupotomies" OR "whole body vibration training").mp. | 29. ("Acupotomies" OR "whole body vibration training").mp. |
| 30. ("Triglycerides" OR "Total cholesterol") | 30. TS=("Triglycerides" OR "Total cholesterol") | 30. ("甘油三酯" OR "总胆固醇") | 30. ("Triglycerides" OR "Total cholesterol").mp. | 30. ("Triglycerides" OR "Total cholesterol").mp. | 30. ("Triglycerides" OR "Total cholesterol").mp. |
| 31. ("HDL Lipoproteins" OR "High Density Lipoprotein") | 31. TS=("HDL Lipoproteins" OR "High Density Lipoprotein") | 31. ("高密度脂蛋白" OR "高密度脂蛋白") | 31. ("HDL Lipoproteins" OR "High Density Lipoprotein").mp. | 31. ("HDL Lipoproteins" OR "High Density Lipoprotein").mp. | 31. ("HDL Lipoproteins" OR "High Density Lipoprotein").mp. |
| 32. ("Density Lipoprotein, High" OR "Lipoprotein, High Density") | 32. TS=("Density Lipoprotein, High" OR "Lipoprotein, High Density") | 32. ("高密度脂蛋白" OR "高密度脂蛋白") | 32. ("Density Lipoprotein, High" OR "Lipoprotein, High Density").mp. | 32. ("Density Lipoprotein, High" OR "Lipoprotein, High Density").mp. | 32. ("Density Lipoprotein, High" OR "Lipoprotein, High Density").mp. |
| 33. ("High-Density Lipoprotein" OR "Lipoprotein, High-Density") | 33. TS=("High-Density Lipoprotein" OR "Lipoprotein, High-Density") | 33. ("高密度脂蛋白" OR "高密度脂蛋白") | 33. ("High-Density Lipoprotein" OR "Lipoprotein, High-Density").mp. | 33. ("High-Density Lipoprotein" OR "Lipoprotein, High-Density").mp. | 33. ("High-Density Lipoprotein" OR "Lipoprotein, High-Density").mp. |
| 34. ("alpha-Lipoprotein" OR "alpha Lipoprotein") | 34. TS=("alpha-Lipoprotein" OR "alpha Lipoprotein") | 34. ("α-脂蛋白" OR "α-脂蛋白") | 34. ("alpha-Lipoprotein" OR "alpha Lipoprotein").mp. | 34. ("alpha-Lipoprotein" OR "alpha Lipoprotein").mp. | 34. ("alpha-Lipoprotein" OR "alpha Lipoprotein").mp. |
| 35. ("alpha-Lipoproteins" OR "alpha Lipoproteins") | 35. TS=("alpha-Lipoproteins" OR "alpha Lipoproteins") | 35. ("α-脂蛋白" OR "α-脂蛋白") | 35. ("alpha-Lipoproteins" OR "alpha Lipoproteins").mp. | 35. ("alpha-Lipoproteins" OR "alpha Lipoproteins").mp. | 35. ("alpha-Lipoproteins" OR "alpha Lipoproteins").mp. |
| 36. ("Heavy Lipoproteins" OR "Lipoproteins, Heavy") | 36. TS=("Heavy Lipoproteins" OR "Lipoproteins, Heavy") | 36. ("重脂蛋白" OR "重脂蛋白") | 36. ("Heavy Lipoproteins" OR "Lipoproteins, Heavy").mp. | 36. ("Heavy Lipoproteins" OR "Lipoproteins, Heavy").mp. | 36. ("Heavy Lipoproteins" OR "Lipoproteins, Heavy").mp. |
| 37. ("High-Density Lipoproteins" OR "High Density Lipoproteins") | 37. TS=("High-Density Lipoproteins" OR "High Density Lipoproteins") | 37. ("高密度脂蛋白" OR "高密度脂蛋白") | 37. ("High-Density Lipoproteins" OR "High Density Lipoproteins").mp. | 37. ("High-Density Lipoproteins" OR "High Density Lipoproteins").mp. | 37. ("High-Density Lipoproteins" OR "High Density Lipoproteins").mp. |
| 38. ("Lipoproteins, High-Density" OR "alpha-1 Lipoprotein") | 38. TS=("Lipoproteins, High-Density" OR "alpha-1 Lipoprotein") | 38. ("高密度脂蛋白" OR "α-1脂蛋白") | 38. ("Lipoproteins, High-Density" OR "alpha-1 Lipoprotein").mp. | 38. ("Lipoproteins, High-Density" OR "alpha-1 Lipoprotein").mp. | 38. ("Lipoproteins, High-Density" OR "alpha-1 Lipoprotein").mp. |
| 39. ("LDL Lipoproteins" OR "beta-Lipoprotein") | 39. TS=("LDL Lipoproteins" OR "beta-Lipoprotein") | 39. ("低密度脂蛋白" OR "β-脂蛋白") | 39. ("LDL Lipoproteins" OR "beta-Lipoprotein").mp. | 39. ("LDL Lipoproteins" OR "beta-Lipoprotein").mp. | 39. ("LDL Lipoproteins" OR "beta-Lipoprotein").mp. |
| 40. ("beta Lipoprotein" OR "Low-Density Lipoprotein") | 40. TS=("beta Lipoprotein" OR "Low-Density Lipoprotein") | 40. ("β-脂蛋白" OR "低密度脂蛋白") | 40. ("beta Lipoprotein" OR "Low-Density Lipoprotein").mp. | 40. ("beta Lipoprotein" OR "Low-Density Lipoprotein").mp. | 40. ("beta Lipoprotein" OR "Low-Density Lipoprotein").mp. |
| 41. ("Lipoprotein, Low-Density" OR "Low Density Lipoprotein") | 41. TS=("Lipoprotein, Low-Density" OR "Low Density Lipoprotein") | 41. ("低密度脂蛋白" OR "低密度脂蛋白") | 41. ("Lipoprotein, Low-Density" OR "Low Density Lipoprotein").mp. | 41. ("Lipoprotein, Low-Density" OR "Low Density Lipoprotein").mp. | 41. ("Lipoprotein, Low-Density" OR "Low Density Lipoprotein").mp. |
| 42. ("beta-Lipoproteins" OR "beta Lipoproteins") | 42. TS=("beta-Lipoproteins" OR "beta Lipoproteins") | 42. ("β-脂蛋白" OR "β-脂蛋白") | 42. ("beta-Lipoproteins" OR "beta Lipoproteins").mp. | 42. ("beta-Lipoproteins" OR "beta Lipoproteins").mp. | 42. ("beta-Lipoproteins" OR "beta Lipoproteins").mp. |
| 43. ("Low-Density Lipoproteins" OR "Lipoproteins, Low-Density") | 43. TS=("Low-Density Lipoproteins" OR "Lipoproteins, Low-Density") | 43. ("低密度脂蛋白" OR "低密度脂蛋白") | 43. ("Low-Density Lipoproteins" OR "Lipoproteins, Low-Density").mp. | 43. ("Low-Density Lipoproteins" OR "Lipoproteins, Low-Density").mp. | 43. ("Low-Density Lipoproteins" OR "Lipoproteins, Low-Density").mp. |
| 44. ("Low Density Lipoproteins" OR "LDL-1") | 44. TS=("Low Density Lipoproteins" OR "LDL-1") | 44. ("低密度脂蛋白" OR "LDL-1") | 44. ("Low Density Lipoproteins" OR "LDL-1").mp. | 44. ("Low Density Lipoproteins" OR "LDL-1").mp. | 44. ("Low Density Lipoproteins" OR "LDL-1").mp. |
| 45. ("LDL(1)" OR "LDL1") | 45. TS=("LDL(1)" OR "LDL1") | 45. ("LDL(1)" OR "LDL1") | 45. ("LDL(1)" OR "LDL1").mp. | 45. ("LDL(1)" OR "LDL1").mp. | 45. ("LDL(1)" OR "LDL1").mp. |
| 46. ("Low-Density Lipoprotein 1" OR "Low Density Lipoprotein 1") | 46. TS=("Low-Density Lipoprotein 1" OR "Low Density Lipoprotein 1") | 46. ("低密度脂蛋白1" OR "低密度脂蛋白1") | 46. ("Low-Density Lipoprotein 1" OR "Low Density Lipoprotein 1").mp. | 46. ("Low-Density Lipoprotein 1" OR "Low Density Lipoprotein 1").mp. | 46. ("Low-Density Lipoprotein 1" OR "Low Density Lipoprotein 1").mp. |
| 47. ("LDL-2" OR "LDL(2)") | 47. TS=("LDL-2" OR "LDL(2)") | 47. ("LDL-2" OR "LDL(2)") | 47. ("LDL-2" OR "LDL(2)").mp. | 47. ("LDL-2" OR "LDL(2)").mp. | 47. ("LDL-2" OR "LDL(2)").mp. |
| 48. ("LDL2" OR "Low-Density Lipoprotein 2") | 48. TS=("LDL2" OR "Low-Density Lipoprotein 2") | 48. ("LDL2" OR "低密度脂蛋白2") | 48. ("LDL2" OR "Low-Density Lipoprotein 2").mp. | 48. ("LDL2" OR "Low-Density Lipoprotein 2").mp. | 48. ("LDL2" OR "Low-Density Lipoprotein 2").mp. |
| 49. ("Low Density Lipoprotein 2") | 49. TS=("Low Density Lipoprotein 2") | 49. ("低密度脂蛋白2") | 49. ("Low Density Lipoprotein 2").mp. | 49. ("Low Density Lipoprotein 2").mp. | 49. ("Low Density Lipoprotein 2").mp. |
| 50. ("Glucose, Blood" OR "Blood Sugar") | 50. TS=("Glucose, Blood" OR "Blood Sugar") | 50. ("血糖" OR "血糖") | 50. ("Glucose, Blood" OR "Blood Sugar").mp. | 50. ("Glucose, Blood" OR "Blood Sugar").mp. | 50. ("Glucose, Blood" OR "Blood Sugar").mp. |
| 51. ("Sugar, Blood" OR "Hemoglobin, Glycated") | 51. TS=("Sugar, Blood" OR "Hemoglobin, Glycated") | 51. ("血糖" OR "糖化血红蛋白") | 51. ("Sugar, Blood" OR "Hemoglobin, Glycated").mp. | 51. ("Sugar, Blood" OR "Hemoglobin, Glycated").mp. | 51. ("Sugar, Blood" OR "Hemoglobin, Glycated").mp. |
| 52. ("Glycohemoglobin" OR "Glycohemoglobins") | 52. TS=("Glycohemoglobin" OR "Glycohemoglobins") | 52. ("糖化血红蛋白" OR "糖化血红蛋白") | 52. ("Glycohemoglobin" OR "Glycohemoglobins").mp. | 52. ("Glycohemoglobin" OR "Glycohemoglobins").mp. | 52. ("Glycohemoglobin" OR "Glycohemoglobins").mp. |
| 53. ("Glycated Hemoglobins" OR "Hemoglobins, Glycated") | 53. TS=("Glycated Hemoglobins" OR "Hemoglobins, Glycated") | 53. ("糖化血红蛋白" OR "糖化血红蛋白") | 53. ("Glycated Hemoglobins" OR "Hemoglobins, Glycated").mp. | 53. ("Glycated Hemoglobins" OR "Hemoglobins, Glycated").mp. | 53. ("Glycated Hemoglobins" OR "Hemoglobins, Glycated").mp. |
| 54. ("Hemoglobin, Glycosylated" OR "Glycosylated Hemoglobin") | 54. TS=("Hemoglobin, Glycosylated" OR "Glycosylated Hemoglobin") | 54. ("糖化血红蛋白" OR "糖化血红蛋白") | 54. ("Hemoglobin, Glycosylated" OR "Glycosylated Hemoglobin").mp. | 54. ("Hemoglobin, Glycosylated" OR "Glycosylated Hemoglobin").mp. | 54. ("Hemoglobin, Glycosylated" OR "Glycosylated Hemoglobin").mp. |
| 55. ("Hb A1a-2" OR "Hemoglobin, Glycated A1a-2") | 55. TS=("Hb A1a-2" OR "Hemoglobin, Glycated A1a-2") | 55. ("Hb A1a-2" OR "糖化血红蛋白A1a-2") | 55. ("Hb A1a-2" OR "Hemoglobin, Glycated A1a-2").mp. | 55. ("Hb A1a-2" OR "Hemoglobin, Glycated A1a-2").mp. | 55. ("Hb A1a-2" OR "Hemoglobin, Glycated A1a-2").mp. |
| 56. ("A1a-2 Hemoglobin, Glycated" OR "Glycated A1a-2 Hemoglobin") | 56. TS=("A1a-2 Hemoglobin, Glycated" OR "Glycated A1a-2 Hemoglobin") | 56. ("糖化血红蛋白A1a-2" OR "糖化血红蛋白A1a-2") | 56. ("A1a-2 Hemoglobin, Glycated" OR "Glycated A1a-2 Hemoglobin").mp. | 56. ("A1a-2 Hemoglobin, Glycated" OR "Glycated A1a-2 Hemoglobin").mp. | 56. ("A1a-2 Hemoglobin, Glycated" OR "Glycated A1a-2 Hemoglobin").mp. |
| 57. ("Hemoglobin, Glycated A1a 2" OR "Glycated Hemoglobin A") | 57. TS=("Hemoglobin, Glycated A1a 2" OR "Glycated Hemoglobin A") | 57. ("糖化血红蛋白A1a 2" OR "糖化血红蛋白A") | 57. ("Hemoglobin, Glycated A1a 2" OR "Glycated Hemoglobin A").mp. | 57. ("Hemoglobin, Glycated A1a 2" OR "Glycated Hemoglobin A").mp. | 57. ("Hemoglobin, Glycated A1a 2" OR "Glycated Hemoglobin A").mp. |
| 58. ("Hemoglobin A, Glycated" OR "Glycohemoglobin A") | 58. TS=("Hemoglobin A, Glycated" OR "Glycohemoglobin A") | 58. ("糖化血红蛋白A" OR "糖化血红蛋白A") | 58. ("Hemoglobin A, Glycated" OR "Glycohemoglobin A").mp. | 58. ("Hemoglobin A, Glycated" OR "Glycohemoglobin A").mp. | 58. ("Hemoglobin A, Glycated" OR "Glycohemoglobin A").mp. |
| 59. ("Hb A1a+b" OR "Hb A1c") | 59. TS=("Hb A1a+b" OR "Hb A1c") | 59. ("Hb A1a+b" OR "Hb A1c") | 59. ("Hb A1a+b" OR "Hb A1c").mp. | 59. ("Hb A1a+b" OR "Hb A1c").mp. | 59. ("Hb A1a+b" OR "Hb A1c").mp. |
| 60. ("Hemoglobin A(1)" OR "Glycosylated Hemoglobin A") | 60. TS=("Hemoglobin A(1)" OR "Glycosylated Hemoglobin A") | 60. ("血红蛋白A(1)" OR "糖化血红蛋白A") | 60. ("Hemoglobin A(1)" OR "Glycosylated Hemoglobin A").mp. | 60. ("Hemoglobin A(1)" OR "Glycosylated Hemoglobin A").mp. | 60. ("Hemoglobin A(1)" OR "Glycosylated Hemoglobin A").mp. |
| 61. ("Hemoglobin A, Glycosylated" OR "Hb A1") | 61. TS=("Hemoglobin A, Glycosylated" OR "Hb A1") | 61. ("糖化血红蛋白A" OR "Hb A1") | 61. ("Hemoglobin A, Glycosylated" OR "Hb A1").mp. | 61. ("Hemoglobin A, Glycosylated" OR "Hb A1").mp. | 61. ("Hemoglobin A, Glycosylated" OR "Hb A1").mp. |
| 62. ("HbA1" OR "Hemoglobin, Glycosylated A1a-1") | 62. TS=("HbA1" OR "Hemoglobin, Glycosylated A1a-1") | 62. ("HbA1" OR "糖化血红蛋白A1a-1") | 62. ("HbA1" OR "Hemoglobin, Glycosylated A1a-1").mp. | 62. ("HbA1" OR "Hemoglobin, Glycosylated A1a-1").mp. | 62. ("HbA1" OR "Hemoglobin, Glycosylated A1a-1").mp. |
| 63. ("A1a-1 Hemoglobin, Glycosylated" OR "Glycosylated A1a-1 Hemoglobin") | 63. TS=("A1a-1 Hemoglobin, Glycosylated" OR "Glycosylated A1a-1 Hemoglobin") | 63. ("糖化血红蛋白A1a-1" OR "糖化血红蛋白A1a-1") | 63. ("A1a-1 Hemoglobin, Glycosylated" OR "Glycosylated A1a-1 Hemoglobin").mp. | 63. ("A1a-1 Hemoglobin, Glycosylated" OR "Glycosylated A1a-1 Hemoglobin").mp. | 63. ("A1a-1 Hemoglobin, Glycosylated" OR "Glycosylated A1a-1 Hemoglobin").mp. |
| 64. ("Hemoglobin, Glycosylated A1a 1" OR "Hb A1a-1") | 64. TS=("Hemoglobin, Glycosylated A1a 1" OR "Hb A1a-1") | 64. ("糖化血红蛋白A1a 1" OR "Hb A1a-1") | 64. ("Hemoglobin, Glycosylated A1a 1" OR "Hb A1a-1").mp. | 64. ("Hemoglobin, Glycosylated A1a 1" OR "Hb A1a-1").mp. | 64. ("Hemoglobin, Glycosylated A1a 1" OR "Hb A1a-1").mp. |
| 65. ("Hemoglobin, Glycated A1b" OR "A1b Hemoglobin, Glycated") | 65. TS=("Hemoglobin, Glycated A1b" OR "A1b Hemoglobin, Glycated") | 65. ("糖化血红蛋白A1b" OR "糖化血红蛋白A1b") | 65. ("Hemoglobin, Glycated A1b" OR "A1b Hemoglobin, Glycated").mp. | 65. ("Hemoglobin, Glycated A1b" OR "A1b Hemoglobin, Glycated").mp. | 65. ("Hemoglobin, Glycated A1b" OR "A1b Hemoglobin, Glycated").mp. |
| 66. ("Glycated A1b Hemoglobin" OR "Hemoglobin, Glycosylated A1b") | 66. TS=("Glycated A1b Hemoglobin" OR "Hemoglobin, Glycosylated A1b") | 66. ("糖化血红蛋白A1b" OR "糖化血红蛋白A1b") | 66. ("Glycated A1b Hemoglobin" OR "Hemoglobin, Glycosylated A1b").mp. | 66. ("Glycated A1b Hemoglobin" OR "Hemoglobin, Glycosylated A1b").mp. | 66. ("Glycated A1b Hemoglobin" OR "Hemoglobin, Glycosylated A1b").mp. |
| 67. ("A1b Hemoglobin, Glycosylated" OR "Glycosylated A1b Hemoglobin") | 67. TS=("A1b Hemoglobin, Glycosylated" OR "Glycosylated A1b Hemoglobin") | 67. ("糖化血红蛋白A1b" OR "糖化血红蛋白A1b") | 67. ("A1b Hemoglobin, Glycosylated" OR "Glycosylated A1b Hemoglobin").mp. | 67. ("A1b Hemoglobin, Glycosylated" OR "Glycosylated A1b Hemoglobin").mp. | 67. ("A1b Hemoglobin, Glycosylated" OR "Glycosylated A1b Hemoglobin").mp. |
| 68. ("Hb A1b" OR "Glycated Hemoglobin A1c") | 68. TS=("Hb A1b" OR "Glycated Hemoglobin A1c") | 68. ("Hb A1b" OR "糖化血红蛋白A1c") | 68. ("Hb A1b" OR "Glycated Hemoglobin A1c").mp. | 68. ("Hb A1b" OR "Glycated Hemoglobin A1c").mp. | 68. ("Hb A1b" OR "Glycated Hemoglobin A1c").mp. |
| 69. ("Hemoglobin A1c, Glycated" OR "Glycosylated Hemoglobin A1c") | 69. TS=("Hemoglobin A1c, Glycated" OR "Glycosylated Hemoglobin A1c") | 69. ("糖化血红蛋白A1c" OR "糖化血红蛋白A1c") | 69. ("Hemoglobin A1c, Glycated" OR "Glycosylated Hemoglobin A1c").mp. | 69. ("Hemoglobin A1c, Glycated" OR "Glycosylated Hemoglobin A1c").mp. | 69. ("Hemoglobin A1c, Glycated" OR "Glycosylated Hemoglobin A1c").mp. |
| 70. ("Hemoglobin A1c, Glycosylated" OR "Fructated Hemoglobins") | 70. TS=("Hemoglobin A1c, Glycosylated" OR "Fructated Hemoglobins") | 70. ("糖化血红蛋白A1c" OR "果糖化血红蛋白") | 70. ("Hemoglobin A1c, Glycosylated" OR "Fructated Hemoglobins").mp. | 70. ("Hemoglobin A1c, Glycosylated" OR "Fructated Hemoglobins").mp. | 70. ("Hemoglobin A1c, Glycosylated" OR "Fructated Hemoglobins").mp. |
| 71. ("Hemoglobins, Fructated" OR "Resistance, Insulin") | 71. TS=("Hemoglobins, Fructated" OR "Resistance, Insulin") | 71. ("果糖化血红蛋白" OR "胰岛素抵抗") | 71. ("Hemoglobins, Fructated" OR "Resistance, Insulin").mp. | 71. ("Hemoglobins, Fructated" OR "Resistance, Insulin").mp. | 71. ("Hemoglobins, Fructated" OR "Resistance, Insulin").mp. |
| 72. ("Insulin Sensitivity" OR "Sensitivity, Insulin") | 72. TS=("Insulin Sensitivity" OR "Sensitivity, Insulin") | 72. ("胰岛素敏感性" OR "胰岛素敏感性") | 72. ("Insulin Sensitivity" OR "Sensitivity, Insulin").mp. | 72. ("Insulin Sensitivity" OR "Sensitivity, Insulin").mp. | 72. ("Insulin Sensitivity" OR "Sensitivity, Insulin").mp. |
| 73. ("Interleukin 6" OR "IL-6") | 73. TS=("Interleukin 6" OR "IL-6") | 73. ("白介素6" OR "IL-6") | 73. ("Interleukin 6" OR "IL-6").mp. | 73. ("Interleukin 6" OR "IL-6").mp. | 73. ("Interleukin 6" OR "IL-6").mp. |
| 74. ("IL6" OR "Tumor Necrosis Factor alpha") | 74. TS=("IL6" OR "Tumor Necrosis Factor alpha") | 74. ("IL-6" OR "肿瘤坏死因子α") | 74. ("IL6" OR "Tumor Necrosis Factor alpha").mp. | 74. ("IL6" OR "Tumor Necrosis Factor alpha").mp. | 74. ("IL6" OR "Tumor Necrosis Factor alpha").mp. |
| 75. ("TNF-alpha" OR "Tumor Necrosis Factor") | 75. TS=("TNF-alpha" OR "Tumor Necrosis Factor") | 75. ("TNF-α" OR "肿瘤坏死因子") | 75. ("TNF-alpha" OR "Tumor Necrosis Factor").mp. | 75. ("TNF-alpha" OR "Tumor Necrosis Factor").mp. | 75. ("TNF-alpha" OR "Tumor Necrosis Factor").mp. |
| 76. ("TNF Superfamily, Member 2" OR "Tumor Necrosis Factor Ligand Superfamily Member 2") | 76. TS=("TNF Superfamily, Member 2" OR "Tumor Necrosis Factor Ligand Superfamily Member 2") | 76. ("TNF超家族成员2" OR "肿瘤坏死因子配体超家族成员2") | 76. ("TNF Superfamily, Member 2" OR "Tumor Necrosis Factor Ligand Superfamily Member 2").mp. | 76. ("TNF Superfamily, Member 2" OR "Tumor Necrosis Factor Ligand Superfamily Member 2").mp. | 76. ("TNF Superfamily, Member 2" OR "Tumor Necrosis Factor Ligand Superfamily Member 2").mp. |
| 77. ("TNFalpha" OR "Cachectin-Tumor Necrosis Factor") | 77. TS=("TNFalpha" OR "Cachectin-Tumor Necrosis Factor") | 77. ("TNFalpha" OR "恶病质-肿瘤坏死因子") | 77. ("TNFalpha" OR "Cachectin-Tumor Necrosis Factor").mp. | 77. ("TNFalpha" OR "Cachectin-Tumor Necrosis Factor").mp. | 77. ("TNFalpha" OR "Cachectin-Tumor Necrosis Factor").mp. |
| 78. ("Cachectin Tumor Necrosis Factor" OR "Cachectin") | 78. TS=("Cachectin Tumor Necrosis Factor" OR "Cachectin") | 78. ("恶病质肿瘤坏死因子" OR "恶病质") | 78. ("Cachectin Tumor Necrosis Factor" OR "Cachectin").mp. | 78. ("Cachectin Tumor Necrosis Factor" OR "Cachectin").mp. | 78. ("Cachectin Tumor Necrosis Factor" OR "Cachectin").mp. |
