# Supplementary material for: Comparative effectiveness of various combined interventions for type 2 diabetes and obesity: a systematic review and network meta-analysis
Source: Front Endocrinol (Lausanne). 2025 Aug 6;16:1462104. doi: 10.3389/fendo.2025.1462104 (PMC12365605; doi:10.3389/fendo.2025.1462104)
Supplement: Supplementary file 5 [file Table1.docx]

| Study | No. | Age | Sex | BMI | Person | Duration(weeks) | Type | Freq. | Volume & Intensity | Exercise methods | Outcome |
| --- | --- | --- | --- | --- | --- | --- | --- | --- | --- | --- | --- |
| Gram 2010 | 1 | 62±10 | ♂♀ | 31.4±4.3 | OB&T2DM | 48 | 6 | 1～2 | The Nordic walking group received personalized training, walking at moderate intensity for at least 30 minutes per session over four months, followed by an interview on their physical activity status. | Nordic Walking | ①③④⑤ |
|  |  | 59±10 | ♂♀ | 32.4±4.1 | OB&T2DM | 48 | 3 |  | The personalized training included strength and aerobics, tailored to test results and goals, using various equipment. Intensity varied per individual, with sessions lasting at least 30 minutes at medium to high intensity (Borg scale 13-14). | Power bikes, rowing machines, stair steppers, strength training equipment | ①③④⑤ |
| Chin 2023 | 2 | 60.5±8.6 | ♂♀ | 27.5±3.2 | OW&T2DM | 12 | 6 | 7 | 7 days a week, 3 times a day, 10 minutes each time, 100 steps/minute each time | Brisk walking | ②③④⑤⑦⑧ |
| Hoseini 2022 | 3 | 47.13±3.12 | ♂ | 29.3±1.43 | OW&T2DM | 8 | 6 | 3 | The personalized training began with 20 minutes at 60% HRmax, increasing to 40 minutes at 75% HRmax after eight weeks, including warm-up and cool-down. Intensity was controlled by HRmax, perceived exertion, and pulse palpation, supervised by an exercise physiologist. | Not explicitly mentioned | ②③④⑤ |
| Kadoglou 2010 | 4 | 56.83±6.76 | ♂♀ | 31.69±4.15 | OB&T2DM | 16 | 6 | 4 | 4 days a week, 30-60 minutes each time | Go fast | ①②③④⑤⑥⑦⑧ |
| Kadoglou 2012 | 5 | 58.4±5.7 | ♂♀ | 32.1±3.77 | OB&T2DM | 12 | 6 | 4 | Aerobic exercise 4 times a week: 10 minutes warm-up, 30 minutes activity, 5 minutes cool-down. Increase intensity to 60-75% of HRmax for the first 4 weeks, 60 minutes each time. | Treadmill, bicycle, calisthenics | ①②③④⑤⑥⑦ |
| Karstoft 2017 | 6 | 65±2 | ♂♀ | BF%:38.3±1.8 | OB&T2DM | 2 | 6 | 5 | Each training session lasted 60 minutes, alternating between low-intensity (54% VO2peak) and high-intensity (89% VO2peak) walking. The training was performed on a treadmill, with oxygen consumption and heart rate regulation monitored. A standardized diet was followed, and blood sugar was monitored. | treadmill | ②③④⑤⑥⑦⑩ |
| Sabag 2020 | 7 | 56.9±2.1 | ♂♀ | 37.5±1.6 | LF & T2DM | 12 | 6 | 3 | The training consists of 19 minutes of cycling: 4 minutes of high intensity (VO2peak 90%), 10 minutes of warm-up (50%), and 5 minutes of cool-down (50%). The high intensity is increased from 1 point to 4 points in the first 4 weeks. It is supervised by a certified expert and heart rate, RPE, and blood pressure are measured regularly. | bike | ①②③④⑤⑥⑦ |
| Kader 2015 | 8 | 43.62±6.17 | ♂♀ | 31.65±5.53 | OB&T2DM | 12 | 6 | 3 | The training consisted of a 5-minute warm-up, 10-30 minutes of treadmill aerobic exercise (heart rate 60-70%), and 10 minutes of cool-down, 3 times a week for 12 weeks, supervised by a physical therapist. | Treadmill | ①⑨⑩ |
| Silva 2012 | 9 | 50.7±9.2 | ♂♀ | 29±6 | OB&T2DM | 6 | 6 | 4 | Training intensity: 80% of maximum heart rate, 4 times a week, 50 minutes each time, including 5 minutes of warm-up, 40 minutes of aerobic exercise (10 minutes of gradual increase, 30 minutes of maintenance), 5 minutes of cool-down. The whole process was supervised by researchers, and the intensity was monitored using a Polar M62 heart rate monitor. | Walking, running | ①②③④⑤⑥ |
| Winding 2018 | 10 | 54±6 | ♂♀ | 28.1±3.5 | OW&T2DM | 11 | 6 | 3 | Each training session lasts 20 minutes, including 10 1-minute high-intensity intervals (95% peak load), followed by 1-minute active recovery (20% peak load). Total training time per week is 75 minutes. | bike | ①②③④⑤⑥⑦⑧ |
| Cassidy 2016 | 11 | 61±9 | ♂♀ | 31±5 | LF & T2DM | 12 | 6 | 3 | A single training session consisted of a 5-minute warm-up (RPE 9-13), followed by five intervals at >80 rpm (RPE 16-17), with the interval time increasing from 2 minutes to 3 minutes and 50 seconds, a 3-minute recovery period after each interval (including 90 seconds of passive recovery, 60 seconds of upper limb exercise and 30 seconds of preparation time), and finally a 3-minute recovery ride. | bike | ②③⑥⑦⑧ |
| Gholami 2018 | 12 | 53±6.4 | ♂ | 28.7±5.6 | T2DM&OW&DPN | 12 | 6 | 3 | Walk or run for 20-45 minutes 3 times a week, including 10-15 minutes warm-up and 5-10 minutes cool-down, maintain a heart rate of 50-70% heart rate reserve, use a Polar PE3000 heart rate monitor to ensure safety, and test blood sugar before training. | Walking, running | ①⑥⑦ |
| Jorge 2011 | 13 | 52.09±8.71 | ♂♀ | 29.29±2.19 | OB&T2DM | 12 | 6 | 3 | Training content: Cycling, with heart rate lactate threshold as the intensity standard, 3 times a week, 60 minutes each time. | bike | ①②③④⑥⑦⑧⑨⑩ |
|  |  | 54.1±8.93 | ♂♀ | 30.89±4.09 | OB&T2DM | 12 | 2 | 3 | Training content: 7 large muscle group resistance training, including leg press, bench press, etc. 3 times a week, 60 minutes each time, muscle strength is measured using the single maximum repetition method. | Resistance training equipment | ①②③④⑥⑦⑧⑨⑩ |
|  |  | 57.90±8.06 | ♂♀ | 31.23±3.88 | OB&T2DM | 12 | 3 | 3 | Training content: Combination of aerobic and resistance training, half the amount of each, including cycling and 7 resistance training. 3 times a week, 60 minutes each time. | The content and resistance are the same as aerobics, but the training volume is halved | ①②③④⑥⑦⑧⑨⑩ |
| Krause 2014 | 1 4 | 52.8±7.2 | ♂ | >30 | OB&T2DM | 16 | 6 | 3 | The training intensity was ventilatory threshold heart rate (55-65% VO_2max_), 3 times a week, 30 minutes each time, for a total of 16 weeks. Outdoor walking, equipped with Polar RS400 heart rate monitor. Return to the laboratory for evaluation and adjustment of heart rate range in the 8th and 16th weeks. | No outdoor | ②③④⑤⑥⑦⑧⑨⑩ |
| Pena 2023 | 1 5 | 13.4±1.4 | ♂♀ | 32.1±3.7 | OB&T2DM | twenty four | 6 | 3 | A single 60-minute aerobic exercise (basketball, football, heart rate <150), a 75-minute nutrition and health education (including behavior change, balanced diet). Guided by YMCA coaches, ensure at least 180 minutes of moderate to high intensity exercise per week. | Aerobic activities such as basketball and football | ①⑨⑩ |
| Ploydang 2023 | 1 6 | 68.9±3.7 | ♂♀ | 24.2±3.6 | OW&T2DM | 12 | 6 | 3 | Exercise in a pool at 34°C–36°C using Nordic walking poles three times a week for 12 weeks. 40 minutes per session, including warm-up and cool-down. 40%–50% heart rate reserve for the first 6 weeks and 50%–60% for the second 6 weeks. |  | ①②③④⑤⑥⑦⑧⑨⑩ |
| Riahy 2024 | 1 7 | 43.9±2.5 | ♂ | 30.0±1.4 | OB&T2DM | 12 | 6 | 3 | Each session consisted of 4 sets of 4 minutes of high intensity exercise (85-95% HRmax), with 3 minutes of rest between sets (50-60% HRmax), walking or running on a treadmill, with a 10-minute warm-up and a 5-minute cool-down. |  | ①⑦⑧ |
| Wang 2023 | 18 | 65.73±3.99 | ♂♀ | 24.03±2.94 | T2DM | 48 | 6 | 3 | Every Wednesday, 60 minutes of training, including 5 minutes of warm-up, 50 minutes of aerobic dance (gradually enhanced) and 5 minutes of cool-down. Supervised by professional coaches, accompanied by music. Use the Karvonen formula to adjust the intensity to moderate. In case of weather or accidents, make-up training on Saturday morning. Diabetes self-management education course once a month. | Aerobic dance training | ①②③④⑤⑥⑦⑧ |
| Baldi 2003 | 19 | 50.1±1.3 | ♂ | 36.4±3.1 | OB&T2DM | 12 | 6 | 3 | Three times a week, 60 minutes of resistance training each time, including exercises such as leg press. 10 reps per set, gradually increase the weight until muscle fatigue. Initial load 40%, increase 5-10% every two weeks to 60%. Professional supervision and adjustment to ensure safety and effectiveness. | Resistance training equipment | ⑥⑦ |
| Bhati 2023 | 2 0 | 52.8±6.82 | ♂♀ | 28.4±3.35 | OW&T2DM&CAN | 12 | 2 | 3 | The intervention group performed resistance training for 12 weeks, 3 times a week, 60 minutes each time. The intensity increased from 65% 1RM to 75%. The content covered 10 major muscle group training, 8-12 times per set, 2-3 sets, and rest for 2-3 minutes. The movements included leg press, abdominal curl, etc. There was a 10-minute bicycle warm-up and low-load stretching before and after the training. | Resistance training equipment | ⑥⑦ |
| Brooks 2007 | twenty one | 66±2 | ♂♀ | 30.9±1.1 | OB&T2DM | 16 | 2 | 3 | Three times a week for 16 weeks at the HNRCA. Each session consisted of 35 minutes of strength training, 3 sets of 8 repetitions using five pneumatic machines. Warm up and cool down for 5 minutes before and after the session. Intensity was 60-80% of 1RM for the first 8 weeks and 70-80% for the second 8 weeks. Postprandial blood glucose was monitored before and after the session. | Resistance training equipment | ⑥⑧ |
| China 2022 | twenty two | 67.6±7.7 | ♂♀ | 24.3±3.4 | SA&T2DM | 12 | 2 | 3 | Training exercises: arm curl, shoulder press, hip abduction, step, tiptoe. Initially use a 0.5 kg sandbag, RPE level 13, 8-15 times/movement. As time goes by, increase the number of times to 20 times, and then increase the sandbag to 1 kg. Exercise every Wednesday, 30 minutes each time, including warm-up and cool-down. |  | ②④⑤⑦ |
| Hsieh 2018 | twenty three | 70.6±4.2 | ♂♀ | 25.6±2.6 | OW&T2DM | 12 | 2 | 3 | 3 times a week, 8 resistance exercises each time, 8-12 reps per set. Including chest press, shoulder press, etc. Initial intensity 40%-50% 1-RM or Borg 12-13 points, 75% 1-RM or Borg 14-16 points in the 12th week. Personalized progress, 60-90 seconds interval. | Resistance training equipment | ②③④⑤⑥⑦ |
| Kadoglou 2012 | twenty four | 61.5±5.4 | ♂♀ | 32.74±4.05 | OB&T2DM | 12 | 2 | 3 | Training every Wednesday, 45-60 minutes. Including 10 minutes of warm-up/cool-down. The main training is eight resistance exercises, such as leg press, chest press, etc. 6-8 times per set, 2-3 sets, intensity is 60-80% of 1-RM. Adjust the training intensity individually, rest for 3 minutes between each set, and rest for 1 minute between exercises. | Resistance training equipment | ①②③④⑤⑥⑦⑧ |
| Li 2024 | 2 5 | 36.6±13.16 | ♂♀ | 29.98±5.77 | OW&T2DM&DPN | 12 | 2 | 5 | Use elastic bands to train, including squats and flexion and extension. Women 15-20 times/movement, men 20-25 times/movement. 3 sets each time, 2-3 minutes interval. 5 times a week, 40 minutes each time. Target heart rate: 60-80% of maximum heart rate. | Portable elastic band | ①②③④⑤⑥⑦⑧ |
|  |  | 37.01±13.02 | ♂♀ | 29.93±6.01 | OW&T2DM&DPN | 12 | 6 | 5 | Warm up for 5 minutes, jog for 30 minutes, and cool down for 5 minutes. Do this 5 times a week, 40 minutes each time. | jogging | ①②③④⑤⑥⑦⑧ |
| Ozairi 2023 | 2 6 | 59.45±8.55 | ♂♀ | 30.56±5.15 | OB&T2DM | 32 | 2 | 3 | Participants performed home resistance training three times per week, which included squats, presses, calf pull-ups, low-rows with resistance bands, lunges, lateral raises with resistance bands, and planks. | elastic band | ①②③④⑤⑦ |
| Plotnikoff 2010 | 2 7 | 55±12 | ♂♀ | 35±8 | OB&T2DM | 16 | 2 | 3 | Participants used Parabody CM3 machines and personalized dumbbells to perform resistance training three times a week for 16 weeks. This included warm-up, core exercises (squats, rowing, etc.), and auxiliary exercises (lunges, pull-downs, etc.). The load increased from 50-60% 1RM in the first week to 80% 1RM in the 16th week to optimize physical fitness. | Parabody CM3 Cable Motion Gym and dumbbells | ①②④⑤⑥⑦ |
| Botton 2018 | 28 | 70.6±6.7 | ♂♀ | 28.2±3.6 | OW&T2DM | 12 | 2 | 3 | Training includes functional exercises (such as squats) and traditional resistance (such as presses). First 4 weeks: functional 10 times/60 seconds, traditional 12 times/15RM/60 seconds. 5-8 weeks as before. 9-12 weeks: functional increase to 15 times/90 seconds, traditional decrease to 10 times/12RM/90 seconds. | Deadweight | ②③④⑤⑥⑦ |
| Byrkjeland 2016 | 29 | 63.5±8.0 | ♂♀ | 29.2±4.0 | OW&T2DM&CAD | 48 | 3 | 3 | Participants participated in comprehensive training for 12 months, with 2 group exercises and 1 home training per week, totaling 150 points. Two-thirds aerobic, one-third resistance, intensity based on Borg RPE, including high-intensity interval training. | Group exercise classes | ②③④⑤ |
| Dieli 2018 | 3 0 | 52.8±10.6 | ♀ | 33.1±5.7 | MetS&OB | 16 | 3 | 3 | Combine aerobic and resistance training, 3 sessions per week: 80 minutes/2 days + 50 minutes/1 day, for 16 weeks, medium to high intensity. Includes aerobic training such as treadmill and rowing machine, and resistance training such as leg press and chest press. Incremental aerobic time and resistance weight. Polar monitors heart rate, and trainers record attendance and exercise time. | Treadmills, bicycles, resistance training equipment | ②③④⑤⑥⑦ |
| Cindy 2010 | 3 1 | 57±7 | ♂♀ | 27.4±4.7 | OW&T2DM | 8 | 2 | 2-3 | The training consists of 9 resistance exercises, targeting the legs, glutes, etc., using machines and free weights. Initial intensity is 65% 1RM, and then 70% after 4 weeks. 50 points per exercise, 10 repetitions per exercise, 3 rounds in total. 2-3 times a week for 8 weeks, a total of 18 exercises. Supervised by a physiotherapist at the Physiotherapy Department of Singapore Hospital, wearing a heart rate monitor. | Use machines and free weights for training. | ①②③④⑤ |
|  |  | 59±7 | ♂♀ | 27.5±4.7 | OW&T2DM | 8 | 6 | 2-3 | Training included treadmill, elliptical machine, and bicycle, with target heart rate increased from 65% to 70% (after 4 weeks). 50 minutes each: 20 minutes on treadmill, 20 minutes on elliptical machine, and 10 minutes on bicycle. 2-3 times a week for 8 weeks (18 times in total). Supervised by a physiotherapist at the Physiotherapy Department of Singapore Hospital, wearing a heart rate monitor. | Treadmill, elliptical machine | ①②③④⑤ |
| Yuan 2018 | 3 2 | 59.0±5.6 | ♂♀ | 26.3±3.1 | OW&T2DM | 96 | 3 | 3 | Combined aerobic and resistance training, three times a week, 60 minutes per session (half aerobic and half resistance), supervised by researchers at the research center. | Elastic band, aerobics | ①②③④⑤⑥⑦ |
|  |  | 58.7±5.5 | ♂♀ | 26.2±3.4 | OW&T2DM | 96 | 2 | 3 | Resistance training covered major muscle groups, using elastic bands, three times a week, 60 minutes per session, intensity 60-80% 1RM, monthly testing, at a research center, supervised by research staff. | elastic band | ①②③④⑤⑥⑦ |
|  |  | 59.1±5.3 | ♂♀ | 26.4±3.2 | OW&T2DM | 96 | 6 | 3 | Aerobic dance training, 3 times a week, 60 minutes per session (including warm-up and stretching), intensity 60-70% HRmax, supervised by researchers at the research center. | Aerobic dance training | ①②③④⑤⑥⑦ |
| Donges 2010 | 3 3 | Adult (no specific age mentioned) | ♂♀ | 27.8±3.9 | OW | 10 | 2 | 3 | 3 exercises per week for 10 weeks. Covering chest, shoulders, back, legs, etc. Initial intensity 70% 10RM, increase every 2 weeks to 75% 10RM. Duration 30-50 minutes, including warm-up and stretching. 8-10 times per set, 2-3 sets in total. | Resistance training equipment | ①②③④⑤⑥⑦⑨ |
|  |  | Adult (no specific age mentioned) | ♂♀ | 30.0±5.5 | OW | 10 | 6 | 3 | 3 sessions per week for 10 weeks. Content: Cycling on a Monark 828E bicycle. Intensity: Start at 70% of maximum heart rate and increase to 75% every 2 weeks. Duration: 30-50 minutes, including warm-up and stretching. Monitor heart rate, resistance, RPE and ride time. | bike | ①②③④⑤⑥⑦⑨ |
| Cauza 2005 | 3 4 | 56.4±1.1 | ♂♀ | 31.3±0.9 | OB&T2DM | 16 | 2 | 3 | 3 training sessions per week, 10 minutes warm-up. Content: Full body strength training. In the early stage, learn the technique and prevent soreness. In the middle and late stages, increase muscle mass. After 3 weeks, do 10-15 reps per set until fatigue, and gradually increase to 6 sets. Exercises include bench press, shoulder press, pull-down, biceps curl, triceps extension, sit-ups, leg press, etc. | Resistance training equipment | ①③④⑤⑥⑦⑧ |
|  |  | 57.9±1.4 | ♂♀ | 33.9±1.3 | OB&T2DM | 16 | 6 | 3 | Three non-continuous training sessions per week, 15 minutes each at the beginning, increasing by 5 minutes every 4 weeks, and reaching 90 minutes/week in the last 4 weeks (excluding warm-up and cool-down). Aerobic training on a bicycle ergometer, with an intensity controlled at 60% of maximum oxygen uptake, and continuous heart rate monitoring. | Power bike | ①③④⑤⑥⑦⑧ |
| Larose 2012 | 3 5 | 53.5±7.3 | ♂♀ | 33.7±6.1 | OB&T2DM | 26 | 3 | 3 | Aerobic training: 3 times a week, 70-75% of maximum heart rate, 25-45 minutes increments, treadmill or bicycle, heart rate monitoring and load adjustment. Resistance training: 3 times a week, 3 sets x 8 times, 14 muscle group movements. More than 8 times to increase weight 2.3-4.5kg. Each time is about 45 minutes, and the actual training is 15-20 minutes. |  | ①②④⑤⑦ |
|  |  | 54.8±7.2 | ♂♀ | 32.6±5.6 | OB&T2DM | 26 | 2 | 3 | The resistance training content was the same as that of the combined training group, including the same training frequency, number of sets, repetitions, and progression methods. |  | ①②④⑤⑦ |
| Li 2022 | 3 6 | 65.15±5 | ♂♀ | 24.27±5.76 | T2DM | twenty four | 6 | 3 | Exercise intervention: 6 months of aerobic dance training, three times a week, each session including warm-up, dance and cool-down. The intensity was 60%-70% of the maximum heart rate, and a treadmill test was performed in the first two weeks to determine the maximum heart rate. | Aerobic dance training | ①③④⑤⑥⑦⑧ |
| Mitranun 2014 | 3 7 | 61.2±2.8 | ♂♀ | 29.6±0.5 | OW&T2DM | 12 | 6 | 3 | 12 weeks of treadmill training, divided into three phases: Week 1-2: 50% VO2peak, 20 minutes training, total 30 minutes, oxygen consumption 33.6±1.8L. Week 3-6: 50% to 80% VO2peak alternating, 4 high intensity, total 30 minutes, oxygen consumption 36.2±1.5L. Week 7-12: 60% to 85% VO2peak alternating, 6 high intensity, total 40 minutes, oxygen consumption 53.7±2.0L. | treadmill | ①②③④⑤⑥ |
| Muller 2018 | 38 | 68.9±7.8 | ♂♀ | 29.1±3.1 | OW&T2DM | 12 | 6 | 3 | Training every Wednesday, 60 minutes each time, including warm-up, resistance (upper limbs, lower limbs), and cool-down. The 12 weeks are divided into three stages: 2 sets/12/15RM in weeks 1-4; 3 sets/12/15RM in weeks 5-8; 3 sets/10/12RM in weeks 9-12. Exercises include leg press, knee flexion and extension, biceps curl, triceps extension, pull-down, shoulder press, bench press, and abdominal curl. | Resistance training equipment, bodyweight | ①②③④⑤⑥⑦⑩ |
| Martins 2010 | 39 | 76.2±7.4 | ♂♀ | 30.1±4.7 | OW&T2DM | 32 | 6 | 3 | Three times a week, 45 minutes each time (including warm-up and cool-down). Intensity increased from 40-50% HRR to 71-85% HRR in weeks 13-16. Heart rate was monitored using Polar S-810i. The main training content was aerobic exercise. | Walking and other aerobic exercise | ①②③④⑤ |
|  |  | 73.2±6.5 | ♂♀ | 30.8±5.3 | OW&T2DM | 32 | 2 | 3 | Moderate-intensity gymnastics and elastic band training were performed three times a week for 45 minutes, with the intensity and content of the training gradually increasing to cover multiple movements such as chair stands, dorsiflexion, hip joint movements, arm presses, etc., for a period of 16 weeks. | Gymnastics, elastic band |  |
| Yuan 2020 | 4 0 | 60.93±5.71 | ♂♀ | 24.69±2.78 | Pre-T2DM | twenty four | 6 | 3 | The six-month training session is 3 times a week, 60 minutes each time, covering warm-up, aerobic dance and stretching. The dance intensity is maintained at 60-70% of the maximum heart rate. The heart rate is monitored in real time, and the training plan will be adjusted if it exceeds the limit. | Aerobic dance training | ①②③④⑤⑥⑦ |
|  |  | 59.91±5.92 | ♂♀ | 24.81±3.19 | Pre-T2DM | twenty four | 2 | 3 | 6-month training plan, 3 times a week, 50 minutes each time. Use elastic bands to perform 13 resistance exercises, 1-2 times a week at the beginning, 50% 1RM intensity, and then increase to 3 times/week, 10-15 times each time, 60% 1RM intensity. Adjust the resistance during training according to the 1RM test results. | elastic band |  |
| Annibalini 2017 | 4 1 | 57±9.1 | ♂ | 28.3±1.5 | OW&T2DM | 16 | 3 | 3 | Training three times a week, including aerobic (treadmill walking, heart rate reserve 40%-65%, time 30-60 minutes) and resistance (leg press and other equipment, 2-4 sets/12-20 times, load 40%-60% of 1RM). Training is carried out at 2 pm, starting 2 hours after meals. | Treadmills, resistance training equipment | ①③④⑤⑥⑦⑨⑩ |
| Bassi 2016 | 4 2 | 49.5±6.1 | ♂♀ | 30.1±5.4 | OB&T2DM | 12 | 3 | 3 | Three times a week, 70 minutes each time, including warm-up, alternating aerobic and resistance training, and cool-down. Aerobic intensity is 60-70% of VO2 max, for 30 minutes; resistance intensity is 60-80% of 1-RM, three sets/10-12 times, targeting the main muscle groups of the upper and lower limbs. | Resistance training equipment, treadmills, stationary bikes, elliptical machines | ①③⑧ |
| Kadoglou 2013 | 4 3 | 56.1±5.3 | ♂♀ | 32.89±3.26 | OB&T2DM | twenty four | 2 | 4 | Four times a week, resistance training, including 8 exercises, 2-3 times per set, 8-10 repetitions each time. First, preparatory test to determine 1-RM, intensity set to 60%-80% 1-RM. Each time for 60 minutes, including 10 minutes of gymnastics warm-up, increase the load to the target in the first 4 weeks and then maintain. | Resistance training equipment | ①③④⑤⑥⑦⑧ |
|  |  | 58.3±5.4 | ♂♀ | 31.55±3.11 | OB&T2DM | twenty four | 6 | 4 | Exercise four times a week, 60 minutes each time: 10 minutes warm-up, 45 minutes aerobic exercise (running, cycling, gymnastics, etc.), 5 minutes cool-down. Increase the intensity to 60%-75% of the maximum heart rate in the first 4 weeks, and then keep it constant. | Treadmill, bicycle, walking, aerobics | ①③④⑤⑥⑦⑧ |
|  |  | 57.9±6.5 | ♂♀ | 31.91±2.93 | OB&T2DM | twenty four | 3 | 4 | Four training sessions per week: one aerobic exercise, one resistance training, and two combined training sessions (including warm-up, half aerobic/half resistance, and cool-down). The intensity is increased gradually to the target in the first four weeks and then kept constant. | As mentioned before | ①③④⑤⑥⑦⑧ |
| Motahari 2023 | 4 4 | 43.9±2.5 | ♂ | 29.6±1.7 | OW&T2DM | 12 | 3 | 3 | Aerobic training: 10 HIITs, 1 minute of high intensity (75-95% of maximum heart rate), 1 minute of rest (40-60%). Resistance training: six exercises, including leg press and bench press, targeting upper and lower limbs. Intensity increased from 40-45% 1RM to 70-80% 1RM, and the number of repetitions decreased from 15-18 to 8-10. | Running, resistance training equipment | ①⑦⑧ |
| Saeidi 2021 | 4 5 | 40-60 | ♂ | 28.51±0.84 | OW&T2DM | 12 | 3 | 3 | Aerobic training is gradually increased, from 10 minutes and VO2peak 60% in the first two weeks to 30 minutes and VO2peak 70% in weeks 9-12. Resistance training includes 8 upper and lower body movements, with an intensity of 60-65% of 1-RM in weeks 1-4 and 70% in weeks 5-12. Each training session: 5-10 minutes warm-up, 45 minutes resistance (1-RM 60-70%), and 30 minutes aerobic (VO2peak 60-70%). The program aims to improve cardiopulmonary function and muscle strength. | Treadmills, resistance training equipment | ①②③④⑤⑥⑨⑩ |
| Silveira-Rodrigues 2021 | 4 6 | 63.9±7.7 | ♂♀ | 28.2±3.6 | OW&T2DM | 8 | 3 | 3 | 8-week plan, training every Wednesday, 2-4 pm. Aerobic: treadmill, load increases from 20 minutes (6 minutes of walking 100%) in weeks 1-2 to 25 minutes (105%-110%) in weeks 7-8. Resistance: 6 exercises, such as reverse grip pull-downs and leg presses, 2-3 sets/time, 8-15 reps/set, rest 60 seconds, and load increases. | Treadmills, resistance training equipment | ②③④⑤⑥⑧ |
| Aminilari 2017 | 4 7 | 45-50 | ♀ | 29.11±1.92 | OW&T2DM | 12 | 2 | 3 | The training is divided into three phases: warm-up (20 minutes of stretching and jogging), main training (three sets of eight times, 50%-55% 1RM, including leg stretching, etc.), and cool-down (running, stretching). Three times a week for 12 weeks, increasing the time by 5 minutes and the intensity by 5% every two weeks. | Resistance training equipment | ①⑥⑧ |
|  |  | 45-50 | ♀ | 30.03±5.48 | OW&T2DM | 12 | 6 | 3 | The training consists of three phases: warm-up with 20 minutes of stretching and jogging, main training with 25 minutes of cycle ergometer (heart rate 50%-55%), and cool-down with running and stretching. The training is done every Wednesday for 12 weeks, increasing the time by 5 minutes and the intensity by 5% every two weeks. | jogging | ①⑥⑧ |
|  |  | 45-50 | ♀ | 29.01±2.57 | OW&T2DM | 12 | 3 | 3 | The training plan includes warm-up, main exercise (aerobic + resistance, half the duration and intensity) and cool-down. Exercise every Wednesday for 12 weeks, increasing the duration by 5 minutes and the intensity by 5% every two weeks. | Resistance training equipment, jogging | ①⑥⑧ |
| Balducci 2010 | 48 | Around 60 years old | ♂♀ | 31.7±4.5 | OB&T2DM | 48 | 3 | 2 | Mixed training twice a week, 150 minutes/week, including aerobic (treadmill, stair stepper, etc.) and resistance (chest press, pull-ups, etc.) training. Intensity is adjusted based on V̇O2max and 1RM. Structured exercise counseling is provided to achieve the recommended amount of physical activity. | Treadmill, stair trainer, elliptical machine, arm or bicycle trainer, resistance training equipment | ①②③④⑤⑥⑦⑧ |
| Bantalebi 2019 | 49 | 55.36±5.94 | ♀ | 29.27±3 | OB&T2DM | 10 | 6 | 3 | Spinning: 5-minute 25-watt warm-up, 4 30-second all-out sprints + 2-minute 50-watt recovery. Complete 3 intervals for the first time, increasing power by 10% each time. | Stationary bike | ①⑥⑦⑧⑨ |
|  |  | 54.14±5.43 | ♀ | 28.68±4.34 | OB&T2DM | 10 | 3 | 3 | Aerobic training: 5 minutes of low heart rate warm-up, increasing from 50% MHR for 20 minutes to 70% MHR for 30 minutes, 10 minutes of cool-down. Resistance training: 1 set of 15 RM in the first two weeks, 2-3 sets of 10-12 RM in the last eight weeks, including 5 items such as double leg press. | Stationary bicycles, resistance training equipment | ①⑥⑦⑧⑨ |
| Leehey 2016 | 5 0 | 65.4±8.7 | ♂ | 36.2±4.8 | OB&T2DM | 52 | 3 | 3 | Interval aerobic training using a treadmill, etc., with individualized prescriptions. Three 90-minute supervised training sessions per week, including 60 minutes of aerobics and 20-30 minutes of lower limb resistance. Home training three times a week for 60 minutes or six times for 30 minutes, with telephone contact and encouragement to see the trainer monthly. | Treadmills, elliptical machines, bicycles, resistance bands, resistance training equipment | ①②③④⑤⑦ |
| Vinetti 2015 | 5 1 | 60.56±5.94 | ♂ | 29.65±4.08 | OB&T2DM | 48 | 3 | 3 | Aerobic training: Mechanical bicycle, individualized heart rate setting, slightly lower than HRGET in the first two months, gradually approaching HRVCP from the third month, interval training, time increased to 35 minutes. Resistance training: upper and lower limbs, chest and back core, 40-50 minutes, from 3 sets of 8 times to 3 sets of 12-15 times. Flexibility training: static stretching before and after resistance training. | Power bikes, elastic bands, resistance training equipment | ①②③④⑥⑦ |
| Johansen 2017 | 5 2 | 53.6±10.1 | ♂♀ | 31.4±3.9 | OB&T2DM | 48 | 3 | 5-6 | Aerobic training 5-6 times per week, 30-60 minutes. Combine 2-3 of these with resistance training. Supervised training for the first 4 months, then tapered. Encourage leisure time activity (at least 10,000 steps per day), monitored with Polar V800. |  | ①②③④⑤⑥⑦ |
| Loimaala 2003 | 5 3 | 53.6±6.2 | ♂ | 29.3±3.7 | OW&T2DM | 48 | 3 | 4 | The subjects will participate in a 12-month comprehensive intervention for type 2 diabetes, including conventional treatment and aerobic and resistance training twice a week. Aerobic training improves cardiopulmonary function, and resistance training exercises large muscle groups. Professional supervision and equipment monitoring ensure that goals are achieved and overall health is improved. | Treadmills, resistance training equipment | ①⑦ |
| Naylor 2016 | 5 4 | 17.3±0.8 | ♂♀ | 36.1±3.9 | OB&T2DM | 12 | 3 | 3 | Three one-hour sessions per week, including aerobic (65-85% HRmax) and resistance (55-70% MVC) training, supplemented by stretching and core strengthening exercises, all supervised by an Advanced Exercise Physiologist (AEP). | Treadmills, stair machines, elliptical machines, bicycle trainers, resistance training equipment | ①⑦ |
| Okada 2010 | 5 5 | 61.9±8.6 | ♂♀ | 25.7±3.2 | T2DM | 12 | 3 | 3-5 | The 3-month exercise intervention, 3-5 times a week, supervised by a physical therapist, includes 10 minutes of warm-up, 40 minutes of aerobic exercise (dance + cycling), 20 minutes of resistance training and 5 minutes of relaxation, aiming to improve health and sports performance. | Aerobics, stationary bicycles, resistance training equipment | ①②③④⑤⑥⑦ |
| Domínguez-Muñoz 2020 | 5 6 | 61.7±8.5 | ♂♀ | 30.37±5.28 | OW&T2DM | 8 | 7 | 3 | Use Galileo 900 vibration platform, train every Wednesday. Stand, knees bent 45 degrees, eyes level, back straight, barefoot. Vibration frequency and time increase: 1 week 8x30 seconds/12.5Hz; 2 weeks 5x60 seconds/13.5Hz; 3 weeks 6x60 seconds/14.5Hz; 4 weeks 7x60 seconds/15.5Hz; 5-6 weeks 8-9x60 seconds/16.5-17.5Hz; 7-8 weeks 9x60 seconds/18.5Hz. Rest 30 seconds between sets, supervised by a physical therapist throughout the process. | Vibration table | ①③④⑤⑥⑦ |
| Lee 2013 | 5 7 | 76.31±4.78 | ♂♀ | 25.29±3.22 | T2DM | 6 | 7 | 3 | The subjects were treated with a Galileo 2000 vibration platform for 3 minutes, 3 times a week for 6 weeks, with a frequency range of 15 Hz to 30 Hz, with an initial amplitude of 2 mm and eventually 1-3 mm. | Vibration table | ⑦ |
| Manimmanakorn 2017 | 58 | 60.9±11.2 | ♂♀ | 25.7±2.4 | OW&T2DM | 12 | 7 | 3 | 12 weeks of Fitvibe Excel vibration training, every Wednesday. Two sets of 6-minute squats, each set of 6 positions, rest for 20 seconds. The load increased from 30Hz, 2mm (peak acceleration: 1.43g) in week 1 to 40Hz, 4mm (7.34g) in week 5, and maintained until week 12. | Vibration table | ①⑥⑦ |
| Michels 2021 | 59 | 59.45±7.1 | ♂♀ | 31.05±4.96 | OW&T2DM | 12 | 7 | 3 | The subjects in the intervention group used a vibration platform every day, with a fixed frequency of 28Hz, a maximum intensity of 0.6g, and a support of 150kg. The subjects were required to stand on both feet with their knees bent at 30 degrees and use it for 20-30 minutes a day. |  | ①②③④⑤⑥⑦ |
| Pozo-Cruz 2014 | 6 0 | 71.6±8.54 | ♂♀ | 30.61±6.8 | OB&T2DM | 12 | 7 | 3 | The intervention group received standard care plus 12 weeks of WBV training, with training every Wednesday and one day off. Globus Phyisio Wave 700 was used, vertical + horizontal vibration. Content: isometric squats for 30 seconds + 8 exercises (such as lunges), vibration frequency and amplitude increased: 1-4 weeks 12Hz/4mm, 30 seconds per movement/30 seconds rest; 5-6 weeks 14Hz, 45 seconds/movement; 7-12 weeks 16Hz, 60 seconds/movement. Non-fasting blood sugar was measured on the first day of intensity increase to ensure safety and effectiveness. |  | ②③④⑤ |
| Ma 2022 | 6 1 | 59.18±3.93 | ♂♀ | 24-28 | Pre-T2DM | 48 | 4 | 3 | According to the "Ba Duan Jin" standard, training is carried out at 7:30-8:30 in the evening, 2-3 sets each time, each set lasting 15-20 minutes. Before and after training, warm up for 10 minutes and stretch and relax for 5-10 minutes. | Ba Duan Jin Mind and Body Exercise | ②③④⑤⑥⑦ |
|  |  | 59.81±4.54 | ♂♀ | 24-28 | Pre-T2DM | 48 | 6 | 3 | The research team and coaches developed an aerobic exercise program based on the guidelines of the American Sports Medical Association and the prescriptions of Luo et al. Participants performed moderate-intensity rhythmic exercise and square dance, wirelessly monitored their heart rate every 15 seconds, and maintained a moderate intensity (heart rate reserve 40-60%, RPE 12-13). | Aerobics | ②③④⑤⑥⑦ |
| Duan Junhong 2012 | 6 2 | 47±7 | ♂♀ | 27.45±2.41 | OW&T2DM | 8 | 4 | 3 | Professional instructors conduct 20 minutes of Ba Duan Jin mind-body exercise | Ba Duan Jin Mind and Body Exercise | ①③④⑥ |
|  |  | 45±9 | ♂♀ | 27.34±2.67 | OW&T2DM | 8 | 6 | 3 | 30 minutes of jogging, brisk walking, etc. | Jogging, brisk walking | ①③④⑥ |
| Guan Yuxiang 2012 | 6 3 | 59.2±8.8 | ♂♀ | 25.1±2.7 | OW&T2DM | 16 | 4 | 7 | 1 hour of Ba Duan Jin mind-body exercise each time | Ba Duan Jin Mind and Body Exercise | ②⑥⑦ |
| Li Qingwen 2016 | 6 4 | 60±3 | ♂♀ | 26.53±1.46 | OW&T2DM | 12 | 4 | 5 | Ba Duan Jin, 45-50 minutes each time, 2 sets in a row, 5 minutes in between, including 5 minutes each for warm-up and post-exercise cool-down, 5 times a week, guided and completed by professional teachers, for 12 consecutive weeks | Ba Duan Jin | ⑥⑦ |
| Luo Fang 2021 | 6 5 | 66.54±10.32 | ♂♀ | 22.36±2.47 | T2DM & HP | 12 | 4 | 3 | Ba Duan Jin training was performed 3 times a week, 40 minutes each time, including 5 minutes of warm-up, 30 minutes of Ba Duan Jin, and 5 minutes of finishing exercise, for 12 consecutive weeks. The training intensity was controlled, and the score of 11 to 13 points on the Borg subjective fatigue score table was appropriate. The patient's heart rate was controlled at 100 to 120 beats/min, and the maximum heart rate was (220-age) × 60% as the upper limit of exercise intensity. | Ba Duan Jin Mind and Body Exercise | ⑥⑦⑨⑩ |
| Ma Yufang 2021 | 6 6 | 60.4±8.6 | ♂♀ | 26.06±3.96 | OW&T2DM | 12 | 4 | 5 | On the basis of conventional treatment (including diabetes education, diet control, hypoglycemic and nerve nutrition therapy), exercise once a day, each time for more than 30 minutes, at least 5 times a week, follow up once a week, provide movement correction guidance to patients and record weekly exercise diaries, and follow up for 12 weeks | Ba Duan Jin Mind and Body Exercise | ②③④⑤⑥⑦ |
| Pan Huashan 2008 | 6 7 | 47±7 | ♂♀ | 27.54±2.81 | OW&T2DM | twenty four | 4 | 5 | The exercise program is Ba Duan Jin (a new fitness exercise issued by the State General Administration of Sport in 2003), with an exercise frequency of 5 days a week, once in the morning and once in the evening, 2 times each time, with a 2-minute rest between each time. Including the warm-up exercises at the beginning and the finishing exercises at the end, one exercise lasts about 45 minutes. | Ba Duan Jin Mind and Body Exercise | ①③④⑥⑦ |
| Wang Yaoguang 2007 | 68 | 57.8±7.5 | ♂♀ | NA | T2DM | twenty four | 4 | 7 | Exercise once a day for 60 minutes each time for 6 months. The intensity of exercise is set according to the "Jungmann" formula. The target heart rate for people over 60 years old is 170-age, and for those under 60 years old is 180-age. The target heart rate must be maintained for more than 5 minutes each time. Adjust the movement posture according to the actual situation to control the intensity and amount of exercise. |  | ②③④⑦ |
| Easy Civilization 2019 | 69 | 53.54±8.21 | ♂♀ | 27.32±3.59 | OW&T2DM | 13 | 6 | >5 | Maintain the original basic treatment (diet control, drug treatment), walk for 30 minutes 1 hour after meals every day, with a walking speed of 80-100 steps/min, 3 times/d, exercise for more than 5 days a week, and treat for a total of 12 weeks. | Brisk walking | ②③④⑤⑥⑦⑧ |
| Zhou Libo 2011 | 7 0 | 67.4±9.23 | ♂♀ | 31.1±2.18 | OB&T2DM | 12 | 4 | 2 | The Ba Duan Jin intervention group was taught Ba Duan Jin twice a week (Wednesday and Saturday), with exercises demonstrated and each subject was given a media CD. They practiced according to the standard exercises and were guided by a doctor, who corrected incorrect movements. They were required to exercise for half an hour every day (performing two full sets of movements, or repeatedly performing several of the movements), without any restrictions on the venue, for three consecutive months. | Ba Duan Jin Mind and Body Exercise | ①②③④⑤⑥⑦ |
|  |  | 68.13±10.64 | ♂♀ | 30.07±3.29 | OB&T2DM | 12 | 6 | 3-5 | Basic exercise intervention exercise items include aerobic metabolic exercises such as walking, jogging, and climbing stairs. The heart rate after exercise = 170 - age (years old), at least 3 to 5 days a week, with no venue restrictions, for three consecutive months. | Walking, jogging, climbing stairs | ①②③④⑤⑥⑦ |
| Chen 2023 | 7 1 | 67.56±4.99 | ♂♀ | 24.32±3.03 | T2DM | twenty four | 4 | 3 | Intervention: Participation in 24-posture simplified Tai Chi training. Training duration: 60 minutes per session, 3 times per week for 24 weeks. Supervision: Training was performed in a supervised setting. Follow-up exercise: Participants were encouraged to continue exercising after completing 24 weeks of supervised training until the 36-week follow-up assessment. | Tai Chi | ⑥⑦ |
|  |  | 67.46±4.73 | ♂♀ | 23.86±2.9 | T2DM | twenty four | 6 | 3 | Intervention: Participation in brisk walking training. Duration of training: 60 minutes per session, 3 times per week for 24 weeks. Supervision: Training was performed in a supervised setting. Follow-up exercise: Participants were encouraged to continue exercising after completing 24 weeks of supervised training until the 36-week follow-up assessment. | Brisk walking | ①②③④⑤⑥⑦ |
| Bao Qinwen 2016 | 7 2 | 70.4±6.9 | ♂♀ | 25.5±3.1 | T2DM | twenty four | 4 | 2 | The experimental group received Tai Chi therapy in addition to conventional treatment. The treatment period was 6 months. The main items included: Tai Chi, Tai Chi sword, Tai Chi fan and Tai Chi soft ball. The exercise intensity was controlled at 50% to 70% of the maximum heart rate. The exercise frequency was 2 times a week, and each exercise lasted 2 hours. | Tai Chi, Tai Chi sword, Tai Chi fan and Tai Chi soft ball | ①②③④⑤⑥⑦ |
| Zhang 2008 | 7 3 | 57.4±6.2 | ♀ | NA | T2DM | 14 | 4 | 5 | Tai Chi training: 1 hour of exercise per day, 5 days per week, for 14 weeks. Training content: 24 Tai Chi movements, including 10 minutes of warm-up, with the main training content conducted under the guidance of a Tai Chi instructor. | Tai Chi | ②③④⑤⑥ |
| Wade Liang 2012 | 7 4 | 56.0±7.2 | ♂♀ | 25.83±2.06 | OW&T2DM | 12 | 4 | 6 | The experimental group performed a 5-minute warm-up exercise from 15:00 to 16:00 every day, then practiced 36 Tai Chi soft ball exercises for 40 minutes with music, and then relaxed for 15 minutes, for a total of 12 weeks. | Tai Chi Soft Ball | ①②④⑤⑥⑦ |
| Chen 2010 | 7 5 | 57.4±5.8 | ♂♀ | 33.2±4.1 | OB&T2DM | 12 | 6 | 3 | Intervention: Aerobic exercise classes, including 20 minutes of warm-up, 30 minutes of aerobic dance designed by the Ministry of Education of Taiwan for middle-aged and elderly people, and 10 minutes of cool-down. The dance includes steps such as walking and touching steps. | Aerobic dance training | ①②③④⑥⑦⑧ |
|  |  | 59.1±6.2 | ♂♀ | 33.5±4.7 | OB&T2DM | 12 | 4 | 3 | Intervention: Tai Chi practice, learning the simplified version of Chen style 13 postures. The course includes 20 minutes of warm-up, 30 minutes of Tai Chi gymnastics, and 10 minutes of breathing cool-down. Home practice is also required, and DVDs are distributed to assist. | Tai Chi Gymnastics | ①②③④⑥⑦⑧ |
| Sukhee 2012 | 7 6 | 66.05±6.42 | ♂♀ | NA | T2DM&DPN | 12 | 4 | 2 | Intervention: The intervention group participated in the TCD program, which included 5 minutes of warm-up, 5 minutes of Qigong, 40 minutes of Tai Chi, 5 minutes of Qigong, and 5 minutes of cool-down, twice a week for 1 hour each time for 12 weeks. | Tai Chi | ⑥⑦ |
| Youngwanichsetha 2013 | 7 7 | 35.00±5.63 | ♀ | 26.77±3.25 | OW&T2DM | 12 | 4 | 3 | The intervention group performed 50 minutes of Tai Chi Qigong exercises three times a week, including 15 minutes of warm-up, 30 minutes of Lin Housheng style Tai Chi Qigong, and 5 minutes of cool-down exercises. | Tai Chi | ①⑥⑦ |
| Lam 2008 | 78 | 63.2±8.6 | ♂♀ | 32.4±6.7 | OB&T2DM | 12 | 4 | 2 | Tai Chi classes were held twice a week for 1 hour each time for 3 months; then classes were continued once a week for another 3 months. | Tai Chi, Tai Chi sword, Tai Chi fan and Tai Chi soft ball | ②③⑦⑧ |
| Kamat 2023 | 79 | 52.53±6.37 | ♂♀ | 28.21±2.64 | OW&T2DM | 12 | 2 | 2 | 2 training sessions per week for 12 weeks. 45 minutes each session, including warm-up, gymnastics, and cool-down. Contents increase in increments: 5 reps/3 sets of bridge poses in the first 3 weeks; 10 reps/3 sets of push-ups in 4-6 weeks; 10 reps/5 sets of high leg raises in 7-9 weeks; 10 reps/5 sets of side lunges in the last 3 weeks. | Calisthenics | ⑦ |
|  |  | 48.27±7.34 | ♂♀ | 30.2±3.02 | OW&T2DM | 12 | 4 | 2 | The Pilates group trained twice a week for 12 weeks. The first 3 weeks were Pilates 100, 5 times/3 sets; 4-6 weeks added swan dives, 10-20 seconds/3 sets; 7-9 weeks added cat-camel, 10 times/5 sets + 30 seconds for 3 additional sets; the last 3 weeks added single leg stretches, 10 times/5 sets + 10 times/3 sets for 10 additional sets. | Pilates training equipment | ⑦ |
| Gouveia 2021 | 8 0 | 59.1±7.4 | ♂♀ | 27.13±4.76 | OW&T2DM | 8 | 4 | 2 | The intervention group underwent 8 weeks of Pilates training, twice a week, for about 1 hour each time, covering three parts: stretching, core strengthening, and relaxation. The training used equipment such as Cadillac. The initial full-body stretching lasted for 30 seconds each, followed by strengthening the core muscles in stages according to the Souza protocol, gradually increasing the difficulty and frequency, and finally ending with relaxation exercises. | Pilates Equipment | ②③④⑥⑦ |
| Keerthi 2017 | 8 1 | 37.28±6.24 | ♂♀ | 27.65±5.71 | OW&T2DM | 12 | 4 | 3 | Yoga sessions are held three times a week for 45 minutes each time for 12 weeks. The yoga module includes relaxation exercises, sun salutations, various yoga poses, pranayama, meditation and relaxation. | Yoga Practice | ⑥⑧ |
| Rodríguez-Reyes 2022 | 8 2 | 60.0±6.1 | ♂♀ | 28.9±6.1 | OW&T2DM | 12 | 7 | 3 | The experimental group participated in whole body vibration (WBV) training three times a week for 12 weeks in addition to the comprehensive diabetes care of the CAIPaDi protocol. The specific WBV training regimen during the intervention period included strength training, stretching exercises, massage, and relaxation exercises. | Whole body vibration training platform | ①②④⑤⑥⑦ |
| Danasegaran 2021 | 8 3 | 51.95±6.17 | ♂ | 27.28±6.75 | OW&T2DM | 12 | 4 | none | Participants received a structured yoga regimen that included asana and pranayama exercises, performed under the supervision of a certified yoga instructor 5 days a week (Monday to Friday) and practiced at home on the remaining 2 days. They also received regular oral antidiabetic medication (OAD) | Yoga Practice | ①②④⑤⑥⑧ |
| Go wri 2022 | 8 4 | 45～58 | ♂♀ | 25～29.5 | T2DM | 16 | 4 | 4 | The intervention group received an integrated yoga therapy that included yoga postures, breathing techniques (such as moon breathing), and meditation. Classes were held twice a week, and participants were required to practice at home twice a week to maintain continuity and regularity. | Yoga Practice | ①②③④⑤⑥⑦⑧ |
| Hirosaki 2023 | 8 5 | 71.8±6.4 | ♂♀ | 23.4±3.2 | T2DM | 12 | 4 | 8 | The intervention group received standard treatment plus laughter yoga. The sessions were held once a week for the first 4 weeks and once every 2 weeks for the next 8 weeks, for a total of 8 sessions. The course consisted of a 30-minute lecture and 60-minute laughter yoga, with the same content and instruction by a certified trainer. | Laughter Yoga | ①⑦ |
| Kaur 2021 | 8 6 | 47.77±9.59 | ♂♀ | 28.59±5.75 | OW&T2DM | 12 | 4 | 7 | Interventions: The intervention group implemented a three-month diabetes yoga program (DYP) including prayer, yoga poses, breathing, and meditation, with daily training for 60 minutes, once a day. | Yoga Practice | ①②③④⑤⑥⑦ |
| kumpatla 2015 | 8 7 | 41.0±8.7 | ♂♀ | 27.2±4.1 | OW&T2DM | 12 | 4 | 7 | Intervention measures: The intervention group practiced yoga for 30 minutes a day, including Vakrasana and other asanas, each lasting 10 seconds and repeated 3-5 times, aiming to strengthen the spine, promote digestion, and lower blood sugar. ​​ | Yoga Practice | ①②③④⑤⑥⑦ |
| Patil 2019 | 88 | 25.57±7.36 | ♂♀ | 23.19±2.52 | Pre-T2DM | 8 | 4 | 5 | Yoga intervention: The intervention group practiced yoga for 1 hour every morning, 5 days a week, for 8 weeks. The content included relaxation, sun worship, asanas, relaxation techniques, breathing and meditation. | Yoga Practice | ⑥⑧ |
| Saberipour 2020 | 89 | 49.91±9.42 | ♂ | 28.06±3.31 | OW&T2DM | 8 | 4 | 3 | 60 minutes three times a week for 8 weeks. Includes warm-up, body and breathing exercises, relaxation and meditation. | Yoga | ①②③④⑤⑥ |
|  |  | 49.91±9.42 | ♂ | 28.06±3.31 | OW&T2DM | 8 | 6 | 3 | Brisk walking, three times a week, 60 minutes each time, for 8 weeks. | Brisk walking | ①②③④⑤⑥ |
| Sharma 2020 | 9 0 | 58.46±7.31 | ♂♀ | 28.14±3 | OW&T2DM | twenty four | 4 | 5 | Intervention: Yoga for 40 minutes at least five times a week for six months, including prayer, yoga postures, breathing exercises and meditation to strengthen the balance of mind and body. | Yoga | ①②③④⑤⑥ |
| Hegde 2020 | 9 1 | 57.10±5.99 | ♂♀ | 26.4±0.7 | OW&T2DM | 12 | 4 | 3-6 | Yoga exercises include Asana, Pranayama and Savasana, covering a variety of postures and breathing methods, 75-90 minutes each time, 6 days a week. Practice guides and video materials are also provided. | Yoga | ①⑥⑦ |
| Viswanathan 2021 | 9 2 | 50.8±8.3 | ♂♀ | 28.1±4.5 | OW&T2DM | 12 | 4 | 5 | Intervention: Yoga training consisted of 30 minutes of asanas and 20 minutes of static relaxation, such as abdominal breathing. | Yoga Practice | ①②③④⑤⑥⑦ |
| Sharma 2023 | 9 3 | 52.9±7.9 | ♂♀ | 27.8±3.1 | OW&T2DM | twenty four | 4 | 5 | Yoga practice: 40 minutes a day, 5 days a week, for 24 weeks. Including relaxation, asanas (forward bends, etc.), breathing exercises and meditation. Asanas are held for 30 seconds, breathing exercises are 20 seconds each round, and meditation includes AUM chanting. | Yoga | ①②③④⑤⑥⑦⑨ |
| Mohammadi 2022 | 9 4 | ≥50 | ♂ | 28.8±2.1 | OW&T2DM | 12 | 2 | 3 | The group performed resistance training on Mondays, Wednesdays and Saturdays, including eight exercises such as leg press and chest press, with increasing intensity and frequency for 12 weeks. | Resistance training equipment | ①②③④⑤⑥⑦ |
|  |  | ≥50 | ♂ | 29.2±2.4 | OW&T2DM | 12 | 6 | 3 | The group performed aerobic training on Tuesdays, Thursdays and Sundays, including warm-up, treadmill training and cool-down. The intensity increased from 50% of heart rate reserve in the first week to 70% in the 12th week for 12 weeks. | Treadmill, bicycle | ①②③④⑤⑥⑦ |
| Arora 2009 | 9 5 | 49.6±5.2 | ♂♀ | 26.99±4.1 | OW&T2DM | 8 | 2 | 2 | Warm up: 5 minutes of static cycling. Main training: seven muscle groups resistance, using dumbbells and other equipment, 10 times per set x 3 sets, increasing the intensity from 60% 1RM to 100% 1RM. Cool down: 5 minutes of static cycling. Monitoring: blood pressure and heart rate, diet and water content, and prevention of hypoglycemia. | Resistance training equipment | ①②③④⑦ |
|  |  | 52.2±9.3 | ♂♀ | 26.23±3.2 | OW&T2DM | 8 | 6 | 3 | Aim for 30 minutes of walking exercise every day, three times a week. | walk | ①②③④⑦ |
| Ku 2010 | 9 6 | 57.8±8.1 | ♀ | 27.4±2.8 | OW&T2DM | 12 | 2 | 5 | Intervention: Elastic band resistance training five times a week, 15-20 repetitions per set, three sets in total, covering 10 exercises to strengthen whole body muscles. | elastic band | ①⑥⑦ |
|  |  | 55.7±6.2 | ♀ | 27.1±2.3 | OW&T2DM | 12 | 6 | 5 | Moderate intensity (3.6-5.2 metabolic equivalents, METs) walking for 60 minutes five times a week. | walk | ①⑥⑦ |
| Kwon 2011 | 97 | 55.5±8.6 | ♀ | 26.7±2.6 | OW&T2DM | 12 | 6 | 5 | Intervention: Five 60-minute walks per week at an intensity of 4-6 METs, weekly visits to the hospital for the first 4 weeks and biweekly visits for the next 8 weeks. | walk | ②③④⑤⑦ |
|  |  | 56.3±6.1 | ♀ | 27.4±2.1 | OW&T2DM | 12 | 2 | 3 | Intervention: Resistance training with Theraband, lowest resistance for the first two weeks, increasing to 40-50% within 12 weeks. 3 sets of 10-15 reps, 3 times per week, including warm-up and cool-down, total duration 60 minutes. | elastic band | ②③④⑤⑦ |
| Li 2024 | 98 | 52.65±8.13 | ♂♀ | 27.71±2.61 | OW&T2DM | 12 | 3 | 2 | Intervention: HIIT + RT twice a week, including 4 minutes of high-intensity intervals, 5 minutes of warm-up/cool-down, and 4 types of resistance training. Cycling bikes were used for HIIT and multi-functional machines were used for RT. Heart rate was monitored to ensure compliance. | Resistance training equipment | ①②④⑤⑥ |
| Lucotti 2011 | 99 | 58.1±9.9 | ♂♀ | 38.8±4.5 | OB&T2DM | 3 | 6 | 5 | Aerobic training: Twice a day, 30 minutes each, including rowing machine and bicycle, at an intensity of 70% of age-predicted maximum heart rate. | Rowing machine, bicycle | ①②③④⑦⑩ |
|  |  | 61.5±11.5 | ♂♀ | 39.9±7.3 | OB&T2DM | 3 | 3 | 5 | Mixed training: twice a day, 45 minutes each, including aerobic (rowing machine + bicycle) and resistance (9 items, half for upper and half for lower limbs, load 40-50% 1RM, 10 times/set for upper limbs, 20 times/set for lower limbs). | Rowing machine, bicycle, resistance training equipment, bodyweight | ①②③④⑦⑩ |
| Magalhães 2022 | 100 | 56.7±8.3 | ♂♀ | 30.1±5.7 | OB&T2DM | 52 | 3 | 3 | Phase 1: 1-4 weeks, moderate intensity cycling for 15-25 minutes. Phase 2: 5-8 weeks, high intensity cycling alternating with low intensity, gradually increasing the intensity. Phase 3: 9-52 weeks, high intensity cycling alternating with low intensity rest. Includes energy expenditure goals, updated regularly. Full body resistance training after each aerobic session, 8 exercises, 10-12 RM per set. | Power bikes, resistance training equipment | ②③④⑤⑨⑩ |
| Martins 2018 | 101 | 64.3±6.7 | ♀ | 64.3±6.7 | OW&T2DM | 12 | 2 | 3 | Training three times a week, including 10 sets of high intensity (step up and down + squats) alternating with low intensity, gradually increasing the number of high intensity sets and reducing the intervals of low intensity. 4 sets of high intensity in the first week, 2 sets per week, fixed 10 sets from the 4th week, and the interval is shortened to 1 minute. The coach adjusts the intensity to ensure that the target is met. | Resistance training equipment, bodyweight | ⑥⑦⑧⑨ |
|  |  | 65.0±6.3 | ♀ | 65.0±6.3 | OW&T2DM | 12 | 3 | 3 | Training three times a week, 60 minutes/time, including 30 minutes of walking and three resistance training. Walking is maintained at a moderate intensity, and the number and time of resistance training are gradually increased. In the first week, walk for 15 minutes + 1 set of resistance, and from the fourth week onwards, walk for 30 minutes + 3 sets of resistance. | Resistance training equipment, bodyweight, walking | ⑥⑦⑧⑨ |
| Bacchi 2013 | 102 | 55.6±2.0 | ♂♀ | 30.5±1.0 | OW&T2DM | 16 | 6 | 3 | Participants trained at the college's fitness center three times a week for 60 minutes each time, maintaining a heart rate of 60%-65% of their maximum heart rate (Karvonen formula). They used their own aerobic equipment and used a heart rate monitor to ensure consistent intensity. | Treadmills, stationary bikes, elliptical trainers | ①②③④⑤⑦ |
|  |  | 56.0±1.9 | ♂♀ | 28.8±1.1 | OW&T2DM | 16 | 2 | 3 | Participants trained at the Academy Fitness Center three times a week, covering 9 major muscle group exercises, using weight machines and free weights. After learning, they performed 10 reps x 3 sets at an intensity of 70%-80% 1RM, with 1 minute rest between sets. | Resistance training equipment | ①②③④⑤⑦ |
| Bacchi 2012 | 103 | 57.2±1.6 | ♂♀ | 29.5±1.1 | OW&T2DM | 16 | 6 | 3 | Training equipment: Cardiovascular training equipment was used. Training intensity: After a learning phase, the workload was gradually increased to 60-65% of the reserve heart rate, estimated using the Karvonen formula, and normalized using a heart rate monitor (Polar S810i). | Power bike | ①②③④⑤⑥⑦ |
|  |  | 55.6±1.7 | ♂♀ | 29.2±1 | OW&T2DM | 16 | 2 | 3 | Content: Weight training machine + free weights, training nine muscle groups, alternating upper and lower limbs and core. Intensity: After learning, 10 times per set x 3 sets, the load gradually increases from 30-50% 1RM to 70-80% 1RM. | Resistance training equipment | ①②③④⑤⑥⑦ |
| Sun 2023 | 10 4 | 50.1±7.3 | ♂♀ | 26.1±4.8 | OW&T2DM | 12 | 6 | 2-3 | Participate in supervised one-hour progressive endurance cycling sessions at 65-80% of maximum heart rate. Perform 2 to 3 times per week for 12 weeks. Track heart rate using a limit monitor and record compliance for each session. During the session, the coach will adjust the exercise load according to the prescribed intensity. | bike | ①②③④⑤⑥⑦⑧ |
| Sukala 2012 | 10 5 | 48±6 | ♂♀ | 42.7±12.1 | OB&T2DM | 16 | 2 | 3 | Includes strength training for all 8 major muscle groups, three times a week, 40-60 minutes each time. | Resistance training equipment | ①②③④⑤⑦⑧ |
|  |  | 51±4 | ♂♀ | 45.0±6.5 | OB&T2DM | 16 | 6 | 3 | Aerobic training: Using a bicycle ergometer, three times a week, 40-60 minutes each time, gradually increasing the intensity to 65-85% of heart rate reserve. | Power bike | ①②③④⑤⑦⑧ |
| Sparks 2013 | 10 6 | 54.2±6.0 | ♂♀ | 33.4±5.8 | OB&T2DM | 36 | 6 | 150min/week | 150 minutes of moderate intensity aerobic exercise per week, consuming 10-12 kcal/kg, intensity 50-80% VO2 peak, including warm-up and cool-down, using a treadmill to measure VO2 peak. | Running, cycling | ①⑦ |
|  |  | 60.4±7.3 | ♂♀ | 33.9±5.2 | OB&T2DM | 36 | 2 | 3 | Resistance training 3 times a week, 2 sets of 4 exercises for upper limbs (bench press, etc.), 3 sets of 3 exercises for legs (leg press, etc.), 2 sets of abdominal and back stretches. 10-12 times per set, and weight gain after completing 12 times in a row. | Resistance training equipment, bodyweight | ①⑦ |
|  |  | 54.1±6.2 | ♂♀ | 37.1±6.8 | OB&T2DM | 36 | 3 | 2 | Perform resistance training twice a week and moderate aerobic training. Each resistance training session includes one set of each of the 9 exercises listed above, with 10-12 repetitions per set. | Resistance training equipment, bodyweight, running, cycling | ①⑦ |
| Moayedi 2023 | 1 07 | 51.64±3.4 | ♀ | 28.95±2.5 | OW&T2DM | 8 | 3 | 3 | Aerobic: 3 times a week for 30-50 minutes, intensity VO_2max_ 50%-70%. Resistance: 3 times a week for 45 minutes, 8 stations, 8-12 times each, covering all muscle groups. | Resistance training equipment, bicycle, running | ③④⑤⑦⑨ |
| Litongxin202 4​ | 1 08 | 56±10.93 | ♂♀ | 27.75±3.46 | OW&T2DM | 12 | 2 | 4 | Under the supervision of medical staff and the guidance of professional sports personnel, exercise the main joints on the comprehensive exercise machine, including dumbbell, barbell push-ups and elastic bands. Repeat each movement 8 to 10 times, each lasting 1 second, with an interval of 2 minutes between each set, for a total of 3 sets. Monitor heart rate and control exercise intensity within the target heart rate range. | Resistance training equipment | ①②③④⑤⑥ |
|  |  | 56±10.93 | ♂♀ | 26.64±3.86 | OW&T2DM | 12 | 6 | 4 | The exercise items are running or cycling, the exercise intensity is 50% to 60% VO_2max_, and the time is 20 minutes to 50 minutes. Professionals provide guidance throughout the exercise process and monitor the heart rate in real time to control it within the target heart rate range. | treadmill | ①②③④⑤⑥ |
| Olioso 2019 | 1 09 | 57.3±6.75 | ♂♀ | 29.9±3.97 | OW&T2DM | 16 | 6 | 3 | Training: Cardiovascular equipment. Intensity: Increase the load after learning, target heart rate 60-65% reserve heart rate, estimated by Karvonen formula. | Power bike | ①②③④⑤⑥⑦ |
|  |  | 54.2±7.73 | ♂♀ | 29.5±5.56 | OW&T2DM | 16 | 2 | 3 | Weight machines + free weights, 9 exercises covering the main muscle groups, alternating between upper and lower limbs and core. Intensity: 30-50% 1RM during the learning period, then increase to 70-80% 1RM, 10 times per set x 3. | Resistance training equipment, bodyweight | ①②③④⑤⑥⑦ |
| Oliveira 2012 | 11 0 | 52.09±8.71 | ♂♀ | 29.3±2.09 | OW&T2DM | 12 | 6 | 3 | Intervention: Cycling training at a heart rate corresponding to lactate threshold. Training was 20 minutes in the first week and increased by 10 minutes each week until reaching 50 minutes. | bike | ②③④⑤⑥⑦ |
|  |  | 54.1±8.94 | ♂♀ | 31.29±4.08 | OW&T2DM | 12 | 2 | 3 | Whole body large muscle group resistance training, 7 exercises (leg press, etc.). 2 sets x 10 reps, 50% 1RM, 2 minutes rest in the first two weeks. 4 sets x 8-12 reps in weeks 3-12, load adjusted to failure. | Resistance training equipment | ②③④⑤⑥⑦ |
|  |  | 57.9±9.82 | ♂♀ | 31.24±3.88 | OW&T2DM | 12 | 3 | 3 | Combine aerobic and resistance training. For the first two weeks, do 5 minutes of cycling and 1 lap of resistance training (2 sets of 15 reps of each strength exercise), followed by another 5 minutes of cycling. Increase the cycling time each week thereafter. | Bicycles, resistance training equipment | ②③④⑤⑥⑦ |
| Ranasinghe 2021 | 11 1 | 52.0±9.8 | ♂♀ | 26.8±4.4 | OW&T2DM | 12 | 6 | 2 | Interventions: The aerobic training program consisted of alternating brisk walking on a treadmill, stepping up and down on a stepper, and stationary cycling. The intensity of each exercise session was 60-75% of maximum heart rate (HRmax), and heart rate was measured using a Polar heart rate monitor. Each exercise session lasted 75 minutes, twice a week for 12 weeks. | Treadmills, stair machines, stationary bikes | ①②③④⑤⑥⑦⑧ |
|  |  | 49.0±9.2 | ♂♀ | 26.9±4.7 | OW&T2DM | 12 | 2 | 2 | 7 major muscle groups resistance training, 8 times x 3 sets, 50% RM, increasing intensity. Bodyweight + free weight + machine circuit, intensity + 5% every two weeks. | Resistance training equipment, bodyweight | ①②③④⑤⑥⑦⑧ |
| Rezaeeshirazi 2022 | 11 2 | 21.07±2.4 | ♂ | 32.00±1.29 | OB&T2DM | 8 | 6 | 4 | Interventions: Aerobic training on a treadmill, starting at 65% of maximum aerobic power and increasing in intensity by 5% each week until reaching 90% intensity by week 8. Each training session lasted 45 minutes. | treadmill | ①⑥⑧ |
|  |  | 21.29±1.9 | ♂ | 32.64±1.69 | OB&T2DM | 8 | 2 | 4 | Progressive circuit resistance training, 7 exercises for upper and lower body. 2 sets x 15 reps, 50% 1RM in the first 3 weeks; 3 sets x 15 reps, 60% 1RM in the 4th to 6th weeks; 3 sets x 8 reps, 70% 1RM in the last two weeks. 1 minute recovery between each set, 30 minutes in total. | Resistance training equipment | ①⑥⑧ |
| AHMED 2019 | 11 3 | 45.26±3.03 | ♀ | 27.76±1.22 | OW&T2DM | 12 | 7 | 3 | Whole body vibration exercise, Fit vibe Excel, including squats and other postures. 3 times a week, 12 minutes each time (36 minutes in total). Vibration frequency increased from 30Hz/2mm to 40Hz/4mm. | Whole body vibration training platform | ⑧ |
|  |  | 45.66±3.15 | ♀ | 27.67±1.05 | OW&T2DM | 12 | 6 | 3 | Aerobic exercise (cycling), including warm-up and cool-down, cycling for 20-30 minutes, 3 times a week, 30-40 minutes each time. | Stationary bike | ⑧ |
| Sawangwong 2023 | 11 4 | 49.31±13.01 | ♂♀ | 26.06±2.5 | OW&pre-T2DM | 12 | 4 | 3 | Participate in Thailand RD exercise, on-site training for 2 days, 60 minutes per day. After 12 weeks of online intervention, 3 times a week, 60 minutes each time, a total of 36 times, video recording for coach supervision. RD includes slow stretching + deep breathing, divided into three stages: warm-up, main training, and cool-down, each 5/50/5 minutes. | Thai Sports | ①⑥⑦⑨ |
| Firouzjaei 2016 | 1 15 | 42.2±6.3 | ♂♀ | 27.6±2.5 | OW&T2DM | 3 | 5 | 3-4 | The patient received metformin, electroacupuncture, and auricular acupuncture. Electroacupuncture was performed at 9 acupoints, including Zhongwan, at a frequency of 15Hz/10mA for 30 minutes using a Hwato SDZ-II machine. Auricular acupuncture was performed at 6 acupoints, including Sanjiao and hunger points. | Acupuncture, conventional medicine | ①②④⑤⑥⑧⑨⑩ |
| Li 2023 | 1 16 | 53.33±6.02 | ♂♀ | 23.32±1.7 | Pre-T2DM | 12 | 5 | 2 | Acupuncture treatment: On the basis of the above lifestyle intervention, acupuncture treatment was performed twice a week for 50 minutes each time, using acupoints such as Da Cha, Huo Zhu, Huo Lian, Huo Chuan and Zu San Li. | Acupuncture | ①②③④⑤⑥⑦ |
| Chen Yuanxiu 2013 | 1 17 | 54±6 | ♂♀ | 27.45±1.83 | OW&T2DM | 12 | 5 | 2-3 | There are two groups of acupoints, and the needling methods are different. Back-shu points are punctured obliquely for tonification, and cupping is performed without retaining the needle; other points are tonified and drained flatly, with the needle retained for 30 minutes and the needle moved for 10 minutes. 2-3 times a week, 10 times for a course of treatment, with an interval of 3-5 days, for a total of 3 courses of treatment. Combined with conventional drug treatment. | Acupuncture | ①②④⑤⑥⑧ |
| Ding Xun 2013 | 1 18 | 52±8.2 | ♂♀ | 27.4±3.2 | OW&T2DM | 9 | 5 | 3-4 | Acupuncture treatment uses 29/30 gauge needles, selects acupoints such as the Taiyang meridian, and adopts the method of equal tonification and equal drainage. The basic prescription includes acupoints such as the Pishu acupoint. Acupoints are added according to the symptoms: add Tianshu acupoints for gastrointestinal heat; add Fenglong acupoints for spleen deficiency and dampness; add Taichong acupoints for liver depression; add Guanyuan acupoints for kidney deficiency. Needle every other day, retain the needle for 30 minutes, and move the needle once every 10 minutes. A course of treatment is 10 times, with an interval of 3 days, for a total of 3 courses. | acupuncture | ①⑥⑦⑧ |
| Li Yonghua 2013 | 1 19 | 50.11±11.13 | ♂♀ | 29.45±3.45 | OW&T2DM | 8 | 5 | 7 | The treatment group received acupuncture in addition to the control group. The acupoints selected included 14 acupoints such as Zhongwan. The patient lay on his back and pierced the acupoints with an Andy brand needle. The abdomen was pierced 35-65mm according to the thickness of the fat, and the lifting and inserting drainage method was used. The needles were retained for 30 minutes, once a day, and the course of treatment was 2 months. | acupuncture | ①⑥ |
| Tang Xianyu 2009 | 12 0 | 58.6±5.7 | ♂♀ | 30.2±3.82 | OW&T2DM | 8 | 5 | 3-7 | Acupuncture points such as Zhongwan are selected, and additional points are added according to the symptoms. Use Huatuo brand needles, connect to the electroacupuncture instrument, leave the needles in for 30 minutes, once a day for the first 6 days, and once every other day thereafter, for a course of 2 months. | acupuncture | ①②③⑥ |
| Xu Jing 2011 | 12 1 | 41.64±12.4 | ♂♀ | 32.85±3.91 | OW&T2DM | 12 | 5 | 7 | Acupuncture points: upper limbs, shoulder joints, lower limbs, hip joints, abdomen, etc., all on both sides. After disinfection, use a 2-inch stainless steel needle, do not lift or insert the needle into the abdomen, leave the needle in for 30 minutes, once a day, 30 days for one course of treatment, rest for 1 day between courses, a total of 3 courses. | acupuncture | ①⑥ |
| Yang Yuan 2015 | 12 2 | 47±8 | ♂♀ | 29.67±3.26 | OW&T2DM | 3 | 5 | 3 | While maintaining the original drug treatment, abdominal acupuncture therapy was added. Ten acupoints including Zhongwan on both sides were selected. The patient lay on his back, and according to the standard positioning, the needle was pierced straight after disinfection, avoiding the pores and blood vessels, and gently twisted. The needle was retained for 30 minutes and irradiated with TDP, and the treatment was carried out every other day, 3 times a week, for a total of 3 weeks. | acupuncture | ①②③④⑤⑥⑧ |
| Zhou Pingnan | 12 3 | 40.6±9.7 | ♂♀ | 29.2±1.1 | OW&T2DM | 12 | 5 | 7 | In addition to basic treatment, acupuncture treatment is added. Body acupuncture takes Dazhui and other acupoints, punctures Zhongwan and other acupoints while lying on the back, punctures Geshu and other acupoints while lying on the stomach, routine puncture, twisting and flat tonification and flat drainage for 2 minutes, retaining the needle for 30 minutes, and moving the needle 1-2 times during the period. Once a day, 20 times a course of treatment, a total of 3 courses of treatment, and rest for 10 days between courses. | acupuncture | ①⑥⑦⑧ |
| Chen Suhua 2023 | 124 | 51.53±3.25 | ♂♀ | 32.89±3.26 | OB&T2DM | 4 | 2 | 4 | Four supervised resistance training sessions per week, including 8 types, 8-10 repetitions per set, intensity 60-80% 1RM, 60 minutes per session including 10 minutes of preparation. | Resistance training equipment | ①②③④⑤⑥⑦⑧ |
|  |  | 52.3±3.42 | ♂♀ | 31.55±3.11 | OB&T2DM | 4 | 6 | 4 | Intervention: Supervised aerobic training 4 times per week at an intensity of 60-75% of maximum heart rate, 60 minutes per session including warm-up, aerobics, and cool-down. | Running, cycling, aerobics |  |
